# Three-dimensional magnetotelluric modeling of Vulcano Island (Eolie, Italy) and its implications for understanding recent volcanic unrest

**DOI:** 10.1038/s41598-023-43828-x

**Published:** 2023-09-30

**Authors:** Maria Giulia Di Giuseppe, Roberto Isaia, Antonio Troiano

**Affiliations:** INGV-Sezione di Napoli, Naples, Osservatorio Vesuviano Italy

**Keywords:** Natural hazards, Geophysics, Volcanology

## Abstract

This paper presents the results of an original short-period magnetotelluric survey performed on Vulcano Island (Italy). The obtained three-dimensional resistivity model details structures up to 2.5 km depth, hitherto unexplored. The La Fossa caldera area corresponds to a moderate resistive anomaly, which extends down to the resolved depth and likely represents a “conduit-like” structure along which magmatic fluids stall and ascend. Other resistive anomalies characterize volcanic edifices, craters, volcanic conduits, and/or eruptive fissures. In addition, the shallower hydrothermal system is detected as a conductive anomaly. Sharp resistivity contrasts generally characterize caldera faults. A main N‒S alignment characterizes the island sector, where considerable amounts of deep subsurface fluids accumulate and mix with the ascending magmas related to the most recent volcanic dynamics. The volcanological interpretation of such findings significantly contributes to understanding the geophysical and geochemical anomalies detected in the last year, which involved the Vulcano shallow hydrothermal system, highlighting the potential for possible hydrothermal/phreatic eruptive events.

## Introduction

The island of Vulcano is the southernmost subaerially exposed volcanic edifice of the Aeolian archipelago. Vulcano has been responsible for many eruptions throughout history, including at least three phreatic eruptions^1–11^. Considering the tourism value offered by volcano-related phenomena (e.g., hot mud flows and fumaroles) and the high exposure of the local inhabited areas to volcanic activity, the volcanic risks on the island are quite high, even for small eruption events. The most recent eruption on the island occurred between 1888 and 1890 AD, and since then, Vulcano's hydrothermal system has been the site of different volcanic unrest^12–18^. In 2021, the routinely monitored geochemical and geophysical parameters showed significant changes, suggesting a potential increase in magmatic fluids within the shallow geothermal system^17,19^. Such fluctuations involved changes to the isotopic composition of the volcanic gases (a well-known magmatic tracer), temperature changes at both the crater and in groundwater in the fumaroles, the opening of new fumarolic vents, and changes to the rate of soil gas emissions observed at both the crater rim and the foot of the cone^20^.

Moreover, GPS data revealed an expansion of the crater zone (confirmed by InSAR data and geodetic surveys), which was traced to a pressurized source at approximately 350 m below sea level (b.s.l.)^20^. Very long period (VLP) seismicity, centered northward of the Vulcano crater (named the ‘La Fossa’ cone) at an average depth of 750 m b.s.l., has been associated with plausible fluid migration^19^. The subsequent rise in the Vulcano alert level, elevated from green to yellow, has increased scientific attention in the region.

Geophysical investigations concerning the island and the volcano-tectonic characterization of its structures, including the relation between the volcano and the geothermal system, represent one of the primary research topics. In this context, a few papers investigated the Vulcano structural setting in the first 1.5 km depth^21–25^. However, part of the island structure remains poorly constrained, particularly beneath the La Fossa caldera.

To fill such a gap, the present study provides the first three-dimensional electrical resistivity model of the La Fossa caldera subsurface structure. Mapping the local structures through electrical resistivity presents an relevant task, considering that geophysical investigations based on such a parameter have already been shown to be adequate for studying active volcanic environments^26–41^. Through the study of this physical parameter, which is highly sensitive to temperature, porosity, permeability, and the presence of fluids^42,43^, it is possible to evaluate the degree of fluid permeation in buried rock volumes and to reconstruct the subsurface structural characteristics (e.g., faults, fractures, and lithology) that coexist in similar contexts.

Vulcano structures have been resolved down to a depth of 2.5 km through a short-period magnetotelluric (MT) survey. This survey provides original information regarding the main structure of the caldera and the related subsurface fluid circulation. The 3D model highlighted caldera faults, detected as sharp resistivity contrasts, and volcanic edifices, craters, eruptive fissures, and conduit-like volcanic structures presumably operating as preferential pathways for magmatic ascending fluids, at the same time evidencing the main volcano tectonic alignments. The volcanological interpretation of such findings provides relevant clues about the causes of the abovementioned geophysical and geochemical anomalies characterizing the ongoing unrest crises.

## Volcanological framework

**Figure 1:**
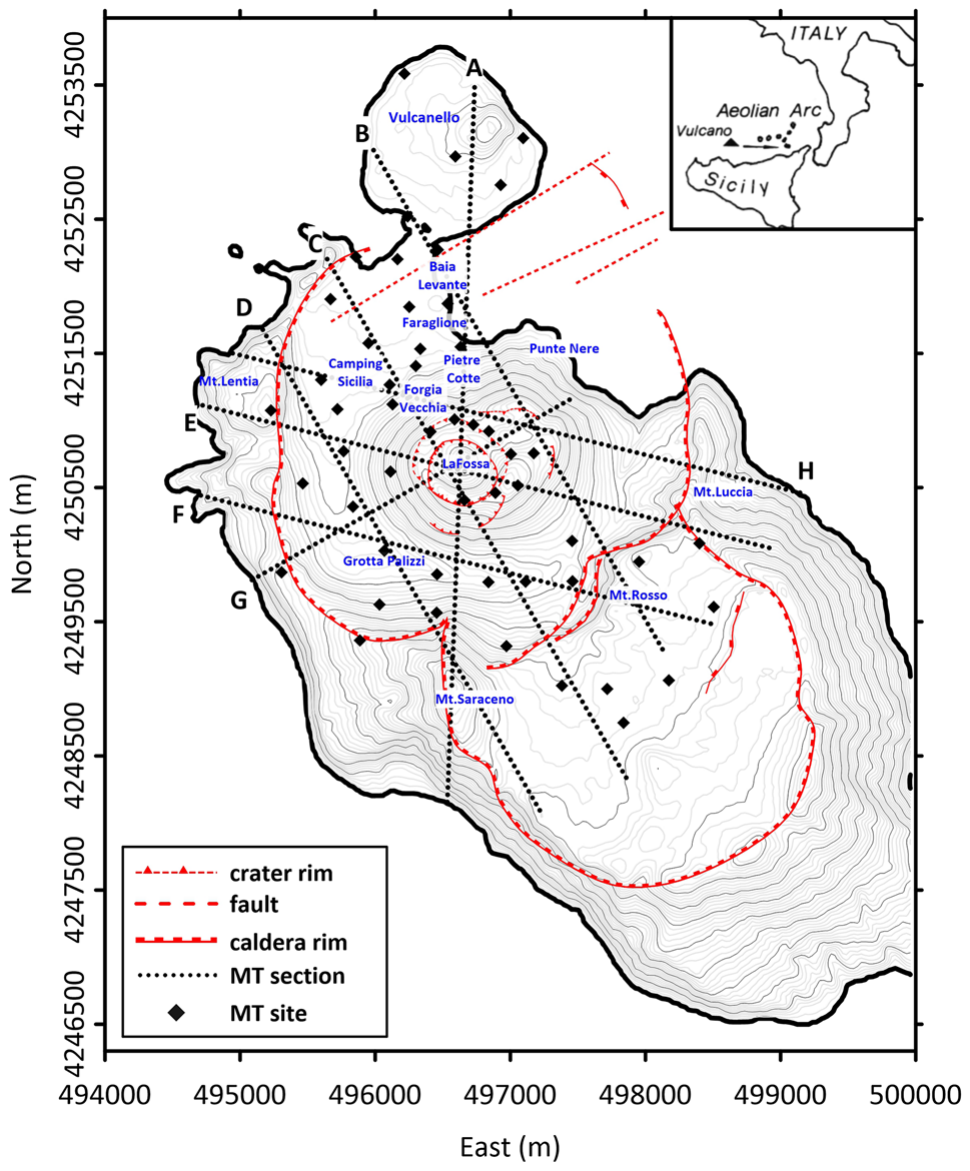
Sketch map of Vulcano. The locations of the main morpho-structural characteristics of the island are evidenced, together with the locations of the 51 MT soundings carried out and the traces of the 2D resistivity sections extracted from the 3D model (see the discussion in the text). Symbols are described in the legend. The figure was created using Surfer 22 commercial software (https://www.goldensoftware.com/products/surfer).

The island of Vulcano (Fig. 1) is the exposed summit of an active NW–SE-elongated composite volcano located in the southernmost sector of the Aeolian archipelago^44^. It is nearly connected with the nearby Lipari complex, from which it is separated only by a shallow water saddle at depths of approximately 50 m b.s.l.^2^. Volcanism spread across eight eruptive epochs separated by volcano-tectonic events, producing two intersecting multistage calderas^2^ and major quiescent intervals. Vulcano rocks range from basalt to rhyolite and show variable alkali contents that roughly increase over time, according to the structural and volcanological evolution of the volcanic system. The locations of the volcanic centers on Vulcano and Lipari Islands have been largely controlled by normal faults (mainly striking NNW‒SSE and N‒S) that were particularly dominant over the last approximately 55 ka and by N‒S structures during the most recent periods of volcanic activity (8 ka and < 2 ka)^45^. This last volcanic period produced eruptions ranging from Strombolian and effusive types to Vulcanian eruptive cycles. In addition, it included a few significant phreatomagmatic eruptions (e.g.^46^ and references therein), with somewhat simultaneous activity located within La Fossa Caldera and the Vulcanello vent (e.g.,^6^). The large phreatic explosions from the La Fossa crater that involved the hydrothermal system produced extensive eruption-like phenomena, including convective columns, ballistic ejection, and pyroclastic density currents, and have been reconstructed by stratigraphic records (such as the Breccia di Commenda event)^4,5,11,46^.

After the last eruption (1888–1890) at the La Fossa crater, a continuous gas discharge developed in the two main fumarolic fields (one on the active crater rim and the other near the Baia di Levante). In addition, widespread diffuse degassing through the soil, both on the crater rim and at a few sites on the caldera floor, also occurred (Grotta dei Palizzi; Camping Sicilia and Baia di Levante; Fig. 1). Unrest phases, including increased fumarole temperature, enlarged fumarolic areas, changes in gas composition, ground deformation, and seismicity, have been recorded and monitored since 1988^47^.

The ongoing unrest showed a sharp increase in degassing activity, both from the crater fumaroles and through the soil, including a drastic increase in the magmatic component of the fumarole composition and the temperature. The monitoring system has recorded local seismicity (VLP events) and ground deformation phenomena centered below the northern sector of the La Fossa crater since August–September 2021^19^. The recorded anomalies are still evident, with the most significant increase in hydrothermal activity observed in Baia di Levante, where abrupt outgassing episodes have led the municipality to prohibit access to portions of the beach.

## The 3D electrical resistivity model

**Figure 2:**
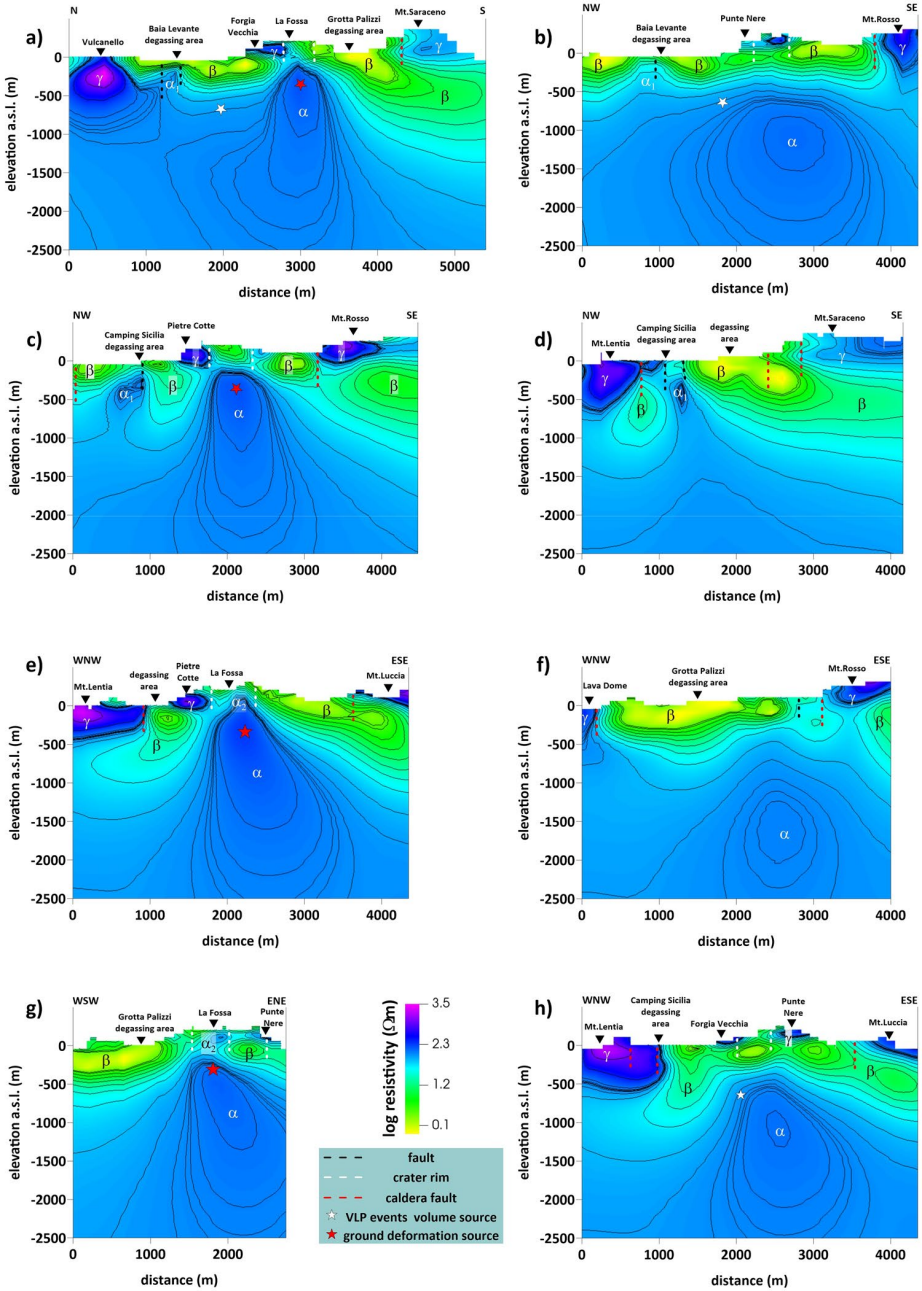
(**a**–**h**), resistivity sections related to the profiles shown with black dashed lines on the Vulcano sketch map of Fig. 1. The contour of electrical resistivity as a function of depth is represented. The adopted color scale is shown; symbols are represented in the legend; letters are explained in the text. The figure was created using the Visit 3.1.2 open-source software (https://wci.llnl.gov/simulation/computer-codes/visit) produced by the Lawrence Livermore National Laboratory (USA) and post-processed using Surfer 22 commercial software (https://www.goldensoftware.com/products/surfer).

Figure 1 shows the locations of the 51 sites where fieldwork and MT data collection were conducted (see Method section). The resulting 3D image of the central-northern sector of Vulcano allowed for the identification of the subsurface electrical resistivity patterns down to a depth of 2.5 km b.s.l. Several resistivity anomalies appeared in the 3D model concerning the volcano's different structures or physical conditions, which can be seen by examining the several 2D slices sketched in Fig. 2, which refer to the traces along the profiles shown in Fig. 1 (dashed black lines). All the resistivity sections presented in Fig. 2 intersect and clearly show the La Fossa caldera border structures, except for the submerged portion outside the area of investigation.

These sections revealed many relevant resistivity anomalies, labeled with Greek letters in Fig. 2. The most significant among these features is the resistivity anomaly on the order of hundreds of Ω m, labeled with α in the sections, whose location closely corresponds with La Fossa crater. This feature has a cylindrical symmetry at the top section and increases in size from approximately 500 m b.s.l. Figure 2a shows that the top of the α anomaly rises to approximately 200–300 m below the La Fossa crater and extends to 600 m to the north and 1000 m to the south of the crater. On the other hand, west of the crater (Fig. 2h), the top of the α structure lies at approximately 1000 m, whereas to the east, it lies at approximately 600 m depth. Finally, this anomaly presents a small apophysis (labeled α_1_), evident in several sections (Fig. 2a–d). The apical part of structure α also appears to be connected to a further, more superficial anomaly of similar resistivity value, indicated by α_2_. The location of this feature corresponds with the uppermost part of the La Fossa cone, primarily evident in Fig. 2e,g.

A low-resistivity layer, denoted as β in the sections of Fig. 2, is detected around structure α and is characterized by an average value of a few tens of Ω m. This anomaly surrounds the top of the α structure and is generally confined above approximately 500 m b.s.l. to the east of the crater, although it extends to a depth of approximately 1000 m toward the west.

Different resistive anomalies of several hundred Ω m (denoted by γ) interrupt the horizontal continuity of conductive layer β. However, these resistivity anomalies appear superficial and are confined to the first 500 m b.s.l., except those localized near Vulcanello (Fig. 2a).

## Discussion/results interpretation

The geometry of the resistivity anomalies sufficiently highlights the different volcano-tectonic lineaments that have characterized the volcanic evolution of the island. Furthermore, these results provide valuable insight into the interaction between the lithostratigraphic setting, fluid circulation, and the current dynamics recorded on the island.

**Figure 3:**
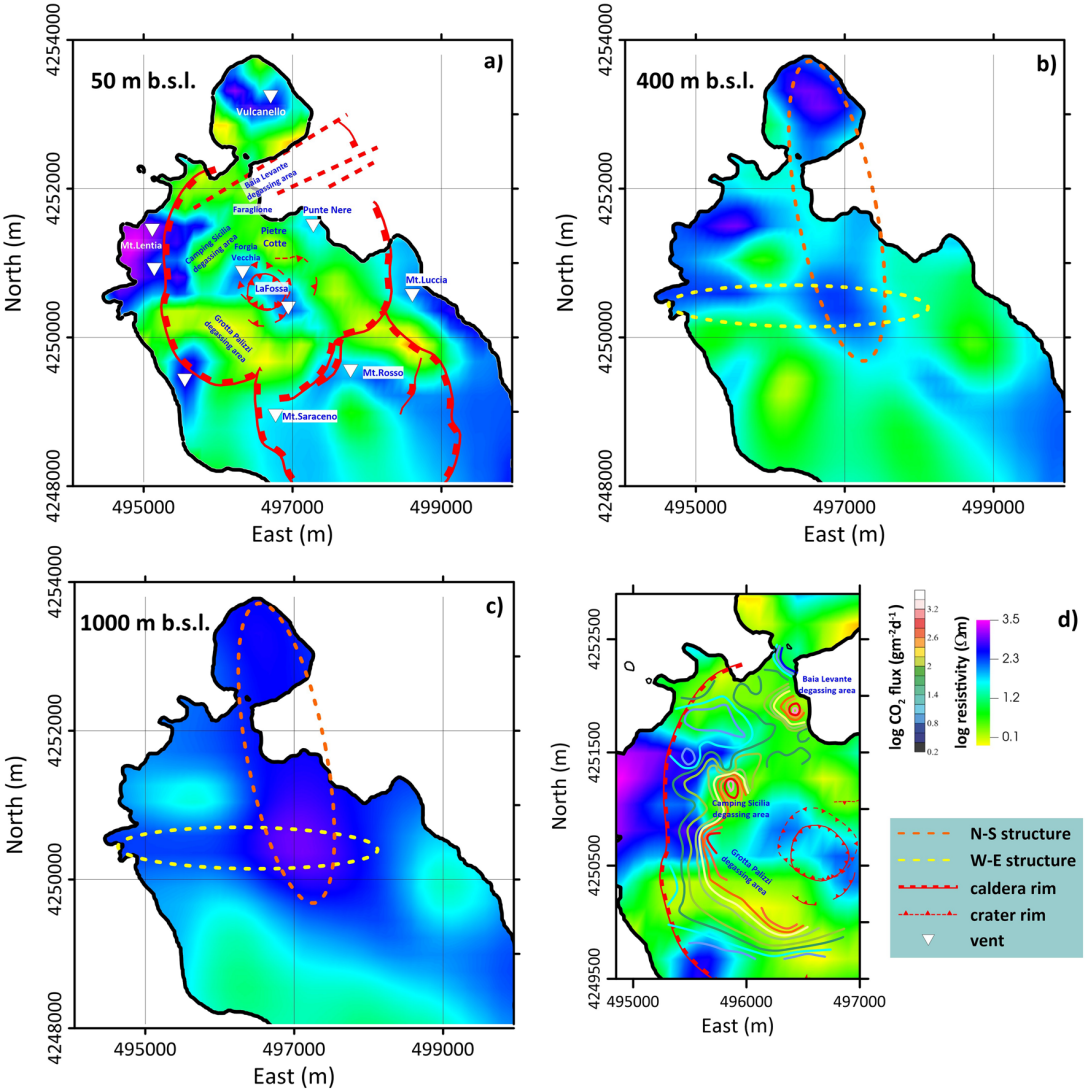
Maps of electrical resistivity related to elevations z = 50 m, z = 400 m, z = 1000 m b.s.l. The map shows: (**a**) Maps of electrical resistivity related to elevations z = 50 m, extracted by the 3D resistivity model. Also shown are the main volcano-tectonic structures extrapolated from De Astis et al.^48^, the eruptive centers inferred from the resistivity anomalies and the main degassing areas; (**b**) and (**c**) maps related to elevations z = 400 m and z = 1000 m b.s.l., respectively. The yellow and orange dashed lines enclose the main trending structures highlighted by the 3D resistivity model. (**d**) Curves extracted from the spatial distribution of CO_2_ fluxes emitted to the ground^19^ superimposed on a map (z = 50 m b.s.l.) extracted from the 3D electrical resistivity model. The figure was created using the Visit 3.1.2 open-source software (https://wci.llnl.gov/simulation/computer-codes/visit) produced by the Lawrence Livermore National Laboratory (USA) and post-processed using Surfer 22 commercial software (https://www.goldensoftware.com/products/surfer).

Resistivity contrasts generally characterized the structures interpreted as caldera faults (Fig. 2; red dotted lines) at the base of the outcropping escarpments identified as the exposed slopes of caldera collapse. Such resistivity contrasts remain evident with geologic structural data regardless of the age of the reconstructed volcano-tectonic collapses^2^. In particular, the correlation of fault scarp (indicated with red dashed lines in Fig. 2) and resistivity contrast seems more evident in the southern sector, likely related to probable reactivations of the older collapse phase. In the northwestern sector, the resistivity contrast seems more internal than at the morphological edge. The calderic borders are also evident in the resistivity maps (Fig. 3), where the N‒S-aligned high-resistivity anomalies are associated with the western La Fossa caldera structure (phase V6^2^).

Moreover, the resistivity sections show that the highest resistivity bodies (named γ in Fig. 2) detected along the surface mainly correspond to a series of volcanic edifices, craters, volcanic conduits, and/or eruptive fissures. The presence of highly resistive bodies in the La Fossa cone is consistent with the results of previous shallow electrical investigations^24,25^. In addition to the domes from the Monte Lentia Formation (Fig. 2d,e, h), the anomalies associated with Monte Saraceno (Fig. 2a,d), Monte Rosso (Fig. 2b,c,f), Monte Luccia (Fig. 2e,h), and Vulcanello (Fig. 2a) are all located along the edges of the caldera. La Fossa, Forgia Vecchia, and Vulcanello's crater areas are aligned within the caldera along a N‒S direction (Fig. 2a). A further resistivity anomaly is detected in the NE sector of the crater summit area (Fig. 2e,g) and likely relates to the N-NE-trending structure. Other resistive bodies are associated with the Punte Nere (Fig. 2b,g,h) and Pietre Cotte (Fig. 2c,e) lavas.

Resistivity maps in the first 50 m b.s.l. (Fig. 3a) also allowed for a suitable identification of the previously described high-resistivity bodies. Below 400 m, two resistivity anomalies that trend approximately E‒W and N‒S are evident, the latter remaining visible to depths greater than 1,000 m b.s.l. (Fig. 3b, c). The E‒W structure corresponds to the alignment of the Lentia-La Fossa eruptive centers (Fig. 3b; yellow dotted line), whereas the N‒S structure (Fig. 3b,c; orange dotted line) describes the alignment of the most recent historical volcanic vents on the island (Vulcanello, Faraglione, La Forgia Vecchia, and La Fossa) as well as the most active presently outgassing structures.

The literature strongly supports the primary alignment found by MT investigations along the N‒S direction. Several authors (e.g.^44,48^) hypothesize that the position of surface magmatic reservoirs responsible for volcanism at Vulcano could be controlled by N‒S secondary faults that play a predominant role in magma upwelling in most superficial layers (< 0.5 km depth)^22^. Ruch et al.^45^ suggested that recent volcano tectonics in the islands of Lipari and Vulcano acted exclusively along a N‒S-directed magmatic corridor that was originally 2 km wide but thinned to 1 km over the last 2 ka. This interpretation concerns a constant E‒W-directed extensional regime and agrees with the geochemical and petrological data, which support the existence of a single stable subsurface magmatic source oriented in a N‒S direction^49^.

Concerning the anomalies described in the previous section, it is worth to note that the general structure of high-temperature active volcano-geothermal systems is associated to zones where the thermal fluid circulation significantly alter the physical properties or the reservoir rocks. The preferential rise of deep fluids through volcanic conduits favours permeability due to volcano-tectonic fracturation and determines high porosity due to the acid leaching process^50^. These processes affect the electrical response of the volcano-geothermal system. The presence of superheated or supercritical fluids flowing in the porous rock volume originates an increasing of the electrical resistivity compared to a liquid-saturated condition^51^.

Moreover, beneath the water table, extremely conductive clay alteration layers (as smectite) are observed in correspondence of temperatures below 220–240 °C, whereas at higher temperatures alteration is mainly in form of minerals as chlorite and illite, which show a higher resistivity signature^42,52^.

Keeping this in mind, we hypothesize that the relatively resistive α structure, centered beneath the La Fossa crater, could be the signature of the presence of a conduit-like structure permeated by deeply stored magma-derived fluids, upwelling toward the crater summit. Coherently with the proposed interpretation, the apical part of this resistive α-body coincides with the location and depth of the recently recorded ground deformation source at Vulcano^20^ (identified with the red star in Fig. 2a,c,e,g). The conductive β anomaly, which generally encompasses the resistive α anomaly, can be on turn interpreted as a the trace of the shallower volcano-geothermal system in which hot saline water and altered rocks coexist^31,34,40,42,53,54^. In particular, we suggest that the top part of the β anomaly, consistent with the typical bell-shaped smectite clay cap, could represent a low-permeability structure below which fluid is distributed^40^. Such interpretation is consistent with the reconstruction of the Vulcano island proposed in Fulignati et al.^50^, particularly for what concerns the peculiar shape of the β anomalies, generally confined to the upper 500 m, which deepens only in the western intracalderic sector to approximately 1000 m b.s.l. This shaping could be related to the difference in the fluid temperature distribution between the the western and the easter sector, as reconstructed in Fulignati et al.^50^ through borehole observations, in combination with the influence of the main volcano-tectonic lineaments, in particular in the area closer to the marginal caldera faults (Fig. 2a,c,d,e,h), which correspond to most fracturing zones where mixing is favored between rising gases and seawater.

Observations made during the drilling of the Vulcano Porto deep well^55^ detected a transition at a depth of approximately 600 m b.s.l. between a shallower sector that consists of more permeable rocks with a low geothermal gradient and convective regime and a more profound sector characterized by more impermeable lavas with a high geothermal gradient, high temperatures, and a conductive regime. This transition is consistent with the depth of the conductive-resistive interface revealed by the 3D resistivity model (Fig. 2). Moreover, the conductive-resistive separation horizon detected in the N‒E sector of La Fossa by the resistivity 3D model matches the source volume location of the VLP seismic event^19^ modeled at an average depth of 750 m b.s.l. (white star in Fig. 2a,b,h).

In addition, in particular, the anomalous Grotta dei Palizzi zone and that of Camping Sicilia are located in the sector affected by the resistivity contrasts that highlight the La Fossa caldera marginal faults. Additionally, the Baia di Levante area is located along the N‒S structure and probably intersects with NE‒SW faults (Figs. 2a–d, 3a).

Figure 3d shows the correlation between the areal distribution map of surface CO_2_ fluxes^19^ and the electrical anomalies detected by the resistivity images. The 50 m b.s.l. resistivity maps show that CO_2_ flow is confined to the inner edge of the higher resistivity bodies, confirming that the maximum expression of ground/soil degassing occurred along the main structures already highlighted in the sections of Fig. 2. As further relief, the areas of maximum degassing anomaly detected during the ongoing unrest crises, such as Grotta dei Palizzi, Camping Sicilia, and Baia di Levante, match very well with the more surficial resistivity anomalies α_2_, highlighting that they occur in fluid rising zones along faults or fault intersections.

## Concluding remarks and implications

The Vulcano 3D resistivity model describes all the island's main volcanic structures in depth and clarifies their geometries, highlighting the relationship with the most significant volcano-tectonic alignments. In addition, these features present evidence of the eruptive history and the present state of the volcano.

**Figure 4:**
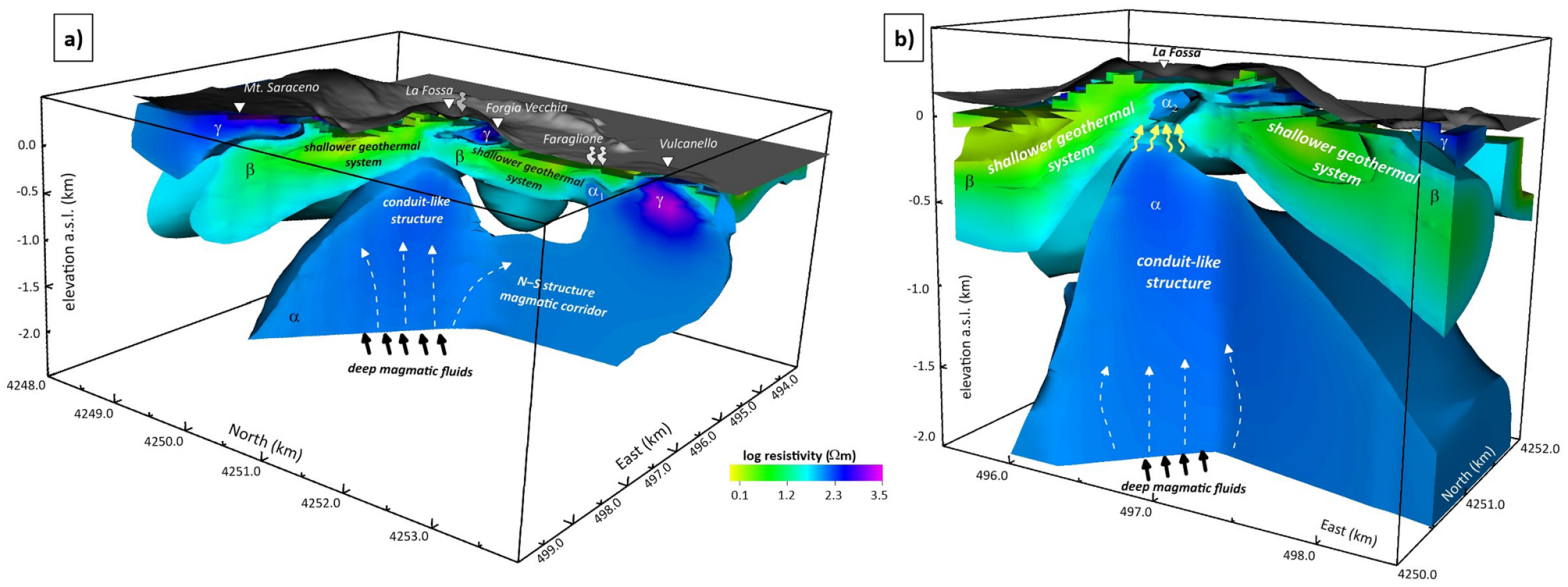
Sketch model of the La Fossa caldera deducted by the 3D resistivity model. Interpretative elements are also superimposed on the model. In particular, (**a**) shows a visualization (view from the east) showing the depth trend of the most resistive bodies aligned in the N‒S direction; (**b**) shows the detected anomalies along an E‒W alignment (view from the south). The common logarithmic scale represents the electrical resistivity. The figure was created using the Visit 3.1.2 open-source software (https://wci.llnl.gov/simulation/computer-codes/visit) produced by the Lawrence Livermore National Laboratory (USA) and post-processed using Surfer 22 commercial software (https://www.goldensoftware.com/products/surfer).

The main characteristics of the 3D resistivity model, which are summarized in Fig. 4, give rise to a few relevant considerations:

- Below the La Fossa main crater, the 3D model detected the presence of a relatively highly resistive body (the α anomaly), extending to a depth of more than 2.5 km (e.g., below the maximum depth investigated by the present survey). The α anomaly likely represents a conduit-like structure that acts as a preferential pathway for deep fluids of magmatic origin to move toward the surface.
- In the crater zone, the top of this conduit corresponds to the location indicated by ground deformation data modeling as the source of the recent 2021 volcanic unrest, which involves the Vulcano hydrothermal system.
- In the northeastern sector, the interface between the α and β anomalies overlaps with the source of the VLP seismic events recorded during the recent crisis, which is likely located at the transition between the magmatic fluid rising channel (imaged by the α anomaly) and the shallower Vulcano geothermal system (imaged by the β anomaly).
- A series of resistivity anomalies, indicated with γ, align mainly along the N‒S direction and overlap with the La Forgia Vecchia and Baia di Levante degassing structure of the N‒NE crater sector. Such structures likely represent the island region where most deep-rising fluids accumulate and mix with the magmas related to the most recent volcanic activity.
- Areas of maximum degassing areas detected during the ongoing unrest crises, such as Grotta Palizzi, Camping Sicilia, and Baia di Levante, match very well with the more surficial resistivity anomalies α_2,_ highlighting that they occur in fluid rising zones along faults or fault intersections.

Furthermore, we recall the direct coupling between the top of the α anomaly and the apical α_2_ anomaly, which appear to have comparable resistivity. Many studies have been performed concerning MT investigations within active volcanic systems worldwide^34,51,56^. In many of these cases, the studies underlined the possible presence of hydrothermally altered rock volumes acting as 'clay caps' at the top of volcanic conduits (e.g.^50^), whose formation has been promoted by an intense circulation of hot fluids. Clays and hydrothermally altered rocks have very high conductivity signatures under wet conditions. However, they could present a different signature if placed in a dry environment. Regarding the α-α_2_ coupling detected in the La Fossa crater case, which consists of two similarly resistive structures, we suggest the presence of a drier rock volume because of the thermodynamic effects caused by deep-originated hot fluid input in the shallower part of the La Fossa hydrothermal system^19,57,58^. Such fluid ingression altered the pressure and temperature of the hydrothermal system in the surrounding rock volume, possibly breaking up the upper cap rock through new or reactivated faults and fractures. The consequent increase in the fluid pressure, changes in fluid circulation, and possible water vaporization altered the fluid circulation in the shallow system, including the water table, and led to an increase in diffuse soil degassing and fumaroles vent, as detected by the monitoring system.

The hot fluid input sourced below La Fossa crater as a possible cause of the shallower hydrothermal system reactivation could also be responsible for the delay recorded few months later between the peak of anomalous degassing activity at the crater and Grotta Palizzi and the peak in the Levante Bay areas^17,58^.

At present, the development of electrical surveys to complement electromagnetic investigations could be aimed at detail with excellent resolution (on the order of meters to tens of meters) of the summit part of the areas of most significant gas emission to help the characterization of the operating processes and their evolution^35,59^.

## Methods

### Magnetotelluric dataset

Magnetotelluric (MT) is a broadband passive geophysical methodology that reconstructs the electrical resistivity spatial distribution by analyzing the synchronous fluctuations of the electric and magnetic fields naturally induced in the subsurface by external sources. MT is a geophysical method that does not require any form of an artificial source: the time variations of the earth’s electric (E) and magnetic (H) fields at a site are recorded simultaneously over a wide range of frequencies. The variations are analyzed to obtain their spectra, and apparent resistivity and phases as a function of frequency are computed from the spectra^58^. In such a way, it is possible to estimate the four-component impedance tensor Z, which relates E and H following the relationship: $$\left( {\begin{array}{*{20}c} {E_{x} } \\ {E_{y} } \\ \end{array} } \right) = \left( {\begin{array}{*{20}c} {Z_{xx} } & {Z_{xy} } \\ {Z_{yx} } & {Z_{yy} } \\ \end{array} } \right)\left( {\begin{array}{*{20}c} {H_{x} } \\ {H_{y} } \\ \end{array} } \right)$$.

The Z tensor depends on the signal frequency and the physical properties of the medium. Its four components, usually separated into diagonal (Z_xx_ and Z_yy_) and nondiagonal (Z_xy_ and Z_yx_) modes, contain information on the electrical structure of the subsurface related to the behavior of the curves of apparent resistivity (ρ_ij_) and phase (φ), defined as $\rho_{ij} = \frac{1}{\omega \mu }\left| {Z_{ij} } \right|^{2} ;$ $\varphi_{ij} = arctan\left( {\frac{{Im\left( {Z_{ij} } \right)}}{{Re\left( {Z_{ij} } \right)}}} \right)$, where μ and *ω* indicate the magnetic permeability and the frequency, respectively. A vast amount of literature describes the basic principles of the MT and its practical aspects. We cite^60–62^, among others.

Figure 1 shows the locations of the 51 sites where fieldwork and MT data collection were performed. Measurements were performed using a Stratagem EH4 instrument produced by Geometrics, which was equipped with additional low-frequency magnetometers and electric dipoles to acquire signals in the MT frequency band. The apparent resistivity and phase curves were estimated in the [10^−1^–10^3^] Hz frequency band. The commercial software MT-Corrector produced by Zond permitted the removal of nonsmooth responses, approximating the frequency dependence of the impedance tensor Z by a smoothing spline.

### Magnetotelluric data inversion

Preliminary analyses of the acquired data focused on data dimensionality, which was estimated through the study of the phase tensor^63^ and via the analysis of the influence of the Vulcano ground-level topography and seafloor bathymetry on the MT dataset^64^. The supplementary materials (SM; Figs. S1, S2) have provided details about such issues.

The 3D code “Modular system for Electromagnetic inversion” (ModEM)^65–67^ has been applied to perform the data inversion, which is currently one of the most applied codes for such purposes. The input data consisted of the four Z tensor modes, reduced to 8 logarithmically spaced frequencies per decade ranging from 1000 to 0.25 Hz. A 5% and 10% fixed floor was considered concerning the error on the nondiagonal and diagonal modes, respectively. The model mesh consisted of a core of 50x50x55 cells with 100 m lateral dimensions. In addition, the mesh model enlarged through 14 padding cells in the E‒W direction and 16 in the N‒S direction, whose lateral dimensions progressively increased by a factor of 1.3. Layer thickness started from 10 m, increasing to 60 m in correspondence with the sea level, up to 250 m at 3 km depth, and up to 12 km at the bottom of the model (set to 100 km). The high-resolution bathymetry of the Vulcano coastline was included as a priori information and kept fixed during the inversion. A resistivity value of 0.3 Ω m was used for the seawater.

The starting model consisted of a half-space of assigned resistivity. To select the most profitable resistivity value for such a half-space, different trial inversions were launched, and the inversion code seems to converge toward models that are quite similar in most cases. Eventually, a 100 Ω m resistivity value was selected, corresponding to the best final data fitting. Model regularization employed a smoothing parameter of 0.2 for the horizontal and vertical directions.

The ModEM inversion code took 73 interactions to converge at a 1.98 value for the normalized root mean square (nRMS: see Fig. S3 of the SM). A value of this metric close to 1 is commonly interpreted as a data fit within the range of observational error, that is, in the case of this study, the error floors, which somewhat reduces the statistical significance^29^. The observation fit can also be deduced by comparing the measured data and predicted curves presented in the SM (S4). The consistency of the main features of the preferred inversion model was questioned by performing a series of tests to analyze every significant structure separately using the model perturbation method^64^. The results of the resolution tests are summarized in the SM (Fig. S5).

### Supplementary Information

Supplementary Information.

Supplementary Figure S1.

Supplementary Figure S2.

Supplementary Figure S3.

Supplementary Figure S4.

Supplementary Figure S5.

Supplementary Figure S6.

## Data Availability

All data generated or analyzed during this study are included in this published article [and its supplementary information files]. Following the project data policies, the data will be available and registered in the INGV database “Geoelectric tomographic maps”, INGV Data Management Office (2020). INGV Open Data Registry, the metadata catalog of the Istituto Nazionale di Geofisica e Vulcanologia. Istituto Nazionale di Geofisica e Vulcanologia (INGV). https://doi.org/10.13127/data-registry.
