# Supplementary material for: Three-dimensional magnetotelluric modeling of Vulcano Island (Eolie, Italy) and its implications for understanding recent volcanic unrest
Source: Sci Rep. 2023 Sep 30;13:16458. doi: 10.1038/s41598-023-43828-x (PMC10543375; doi:10.1038/s41598-023-43828-x)
Supplement: Supplementary file 1 — Supplementary Information. [file 41598_2023_43828_MOESM1_ESM.docx]

*Scientific Reports*

Supporting Information for

**Three-dimensional magnetotelluric modeling of Vulcano Island (Eolie, Italy) and its implications for understanding recent volcanic unrest**

M. G. Di Giuseppe^(1)^, R. Isaia^(1,*)^, A. Troiano^(1)^

1 Istituto Nazionale di Geofisica e Vulcanologia, Sezione di Napoli ‘Osservatorio Vesuviano’ – Italy.

**Contents of the supplementary materials.**

This Supporting Information file contains details on the MT prospection presented in the paper.

As the first step after estimating the MT responses, dimensional analysis of the dataset has been performed to investigate the Z tensor properties, which reflect the symmetries of the subsoil structures and particularly their privileged alignments. For such an analysis, the phase tensor (PT), introduced by ^59^, has been derived directly from the Z tensor through the relationship PT=Re(Z)^-1^·Im(Z). The PT is insusceptible to galvanic distortions and can be represented graphically as an ellipse with the principal axes (Φ_max_ and Φ_min_) showing the major-minor axes of the tensor. The polarization ellipses behavior, estimated through the MTpy code ^64,65^, has been reported for a few selected periods in Fig. S1. Such ellipses can be employed to estimate which parts of the impedance tensor could be considered 1D, 2D, or 3D once the ellipticity (λ) and, in the case of nonsymmetric, the skew-angle (β) has been defined as a function of Φ_max_ and Φ_min_ ^66^. A threshold value has been considered for the skew angle β (5 degrees). Such a PT analysis confirms the 3D character of the central sector of Vulcano Island.

The second step analyzes the influence of ground-level topography and seafloor bathymetry on the MT dataset. A sea layer around the survey area severely affects the observed MT responses due to the sharp electrical contrast between seawater and land. The skin depth of the performed MT survey can reach up to a few kilometers, so the sea likely has a substantial influence on observed MT data when the separation distance from the coast is smaller than the skin depth of the frequency of interest ^60^, which is likely the case of the presented survey. Additionally, the undulating topographic features modify the current flow pattern, thus affecting the electrical and magnetic field components to different degrees; in observation sites in this vicinity, the MT response function may become distorted ^67^. A forward modeling study permitted the evaluation of the frequencies most subject to the influence of these two effects and whether these could disturb the diagonal and nondiagonal modes of the **Z** tensor in the same manner. Such forward modeling reconstructed the MT response of a theoretical model of the Vulcano island, which considers the presence of the actual ground-level topography and seafloor bathymetry over an ideal homogeneous half-space of 100 Ωm resistivity. The forward modeling, realized by adopting the ModEM 3D code^59^ and the same model mesh adopted for the inversion procedure (described in the main text), permitted the reconstruction of the corresponding synthetic behavior of the apparent resistivity ρ (log10(Ωm)) and phase Φ (degrees). The simulation evaluates how much the MT responses of the tested model remain substantially unaffected by topography- and bathymetry-related distortion with respect to the one that should characterize the ideal homogeneous half-space (e. g. ρ=100 Ωm and Φ=45°). The results are summarized in Fig. S2, where apparent resistivity and phase maps have been reproduced for all four modes of the Z tensor in correspondence with four different periods. A close look at those maps indicates that the apparent resistivity curves remain substantially unaffected by topography- and bathymetry-related distortion, with minor Zxy mode distortions at about 1 Hz. On the other hand, phases seem to manifest a most severe perturbation, mainly for the diagonal modes. These results have been considered during the inversion phase when the choice of error floors imposed on the data has been made.

As a metric for the fit of the modeled to the observed data, the commonly used normalized root mean square has been adopted, which is defined as $nRMS=\sqrt{\left( N_{d}-1 \right)^{-1}\sum_{j=1}^{N_{d}} \left( \frac{d_{obs,j}-d_{calc,j}}{\sigma_{j}} \right)^{2}}$, where *d_obs,j_* and *d_calc,j_* are observed, and calculated data, respectively, and *σ_j_* represents the errors for all *N_d_* data points ^29^.

A value of this metric close to 1 is commonly interpreted as a data fit within the range of observational error, that is, in the case of this study, the error floors, which somewhat reduces the statistical significance. Figure S3 shows a map of the final nRMS, which achieved a value of about 1.98. The fit to most observations can also be deduced from the fit of the data curves presented in Figure S4.

The resolution of the detected features was questioned by performing a series of tests to separately analyze the α and the β structures, which have been discussed in the text.

First, we adopted the MTpy code to evaluate the penetration depths of the electromagnetic waves at a 3 s period, using the Niblett-Bostick transform ^64^ and references therein), which map is reported in Figure S5. In the second step, we perform synthetic tests on the data sensitivity of these two model parts. Due to the complexity of 3D, the model perturbation method has been used for this. The resistivity for the α and the β anomalies in the preferred inversion model has been modified, decreasing and increasing its value in a range uniformly covering several magnitude orders. Then, many forward modelling runs were launched, one for each resistivity value tested, and the difference between the new nRMS and the one related to the inversion preferred model (about 1.98; see Fig. S3) was checked. If the difference is significant, data are sensitive enough to that part of the model. After this check, it is possible to know if these two parts of the model are well-constrained by the data. The test results are summarized in Figure S5.

**Supplementary Figure captions**

Fig. S1. Maps of the Phase Tensor ellipses. The inner color of the ellipses indicates the value of the Skew angle β (in degrees). The panels refer to 0.001s, 0.01 s, 0.1 s and 1 s, respectively.

Fig. S2. Results of the forward model study aimed to reconstruct the effects of the ground-level topography and seafloor bathymetry on the MT dataset. The actual coastline and undulating topography of the Vulcano island have been superimposed over a 100 Ωm homogeneous half-space. The corresponding synthetic apparent resistivity [log10(Ωm)] and phase [deg] maps (related to each one of the four Z tensor modes) have been evaluated using the ModEM code and subsequently represented for periods of 0.001 s, 0.01 s, 0.1 s, and 1 s, respectively.

Fig. S3. Map of the nRMS misfit values. a) total nRMS on all the elements of the Z tensor. A final value of 1.98 was achieved. b) nRMS maps for each one of the four Z tensor modes.

Fig. S4. Comparison between the apparent resistivity [log10(Ωm)] and phase [deg] curves related to the measured and predicted data.

Fig. S5. Map of the penetration depth of the electromagnetic waves of 1 s period, obtained using the Niblett-Bostick transform.

Fig. S6. Results of resolution tests on the α and the β resistivity anomalies detected by the preferred inversion model. The panel reports the nRMS changes (on the y-axis) vs. resistivity imposed in the α and the β regions of the preferred inversion model (on the x-axis).

**References**

64. Kirkby, A., Zhang, F., Peacock, J., Hassan, R. & Duan, J. The MTPy software package for magnetotelluric data analysis and visualisation. *JOSS* **4**, 1358 (2019).

65. Krieger, L. & Peacock, J. R. MTpy: A Python toolbox for magnetotellurics. *Computers and Geosciences* (2014) doi:10.1016/j.cageo.2014.07.013.

66. Booker, J. R. The Magnetotelluric Phase Tensor: A Critical Review. *Surveys in Geophysics* **35**, 7–40 (2014).

67. Kumar, D., Singh, A. & Israil, M. Necessity of Terrain Correction in Magnetotelluric Data Recorded from Garhwal Himalayan Region, India. *Geosciences* **11**, 482 (2021).
