# Supplementary figures and images for: Three-dimensional magnetotelluric modeling of Vulcano Island (Eolie, Italy) and its implications for understanding recent volcanic unrest
Source: Sci Rep. 2023 Sep 30;13:16458. doi: 10.1038/s41598-023-43828-x (PMC10543375; doi:10.1038/s41598-023-43828-x)

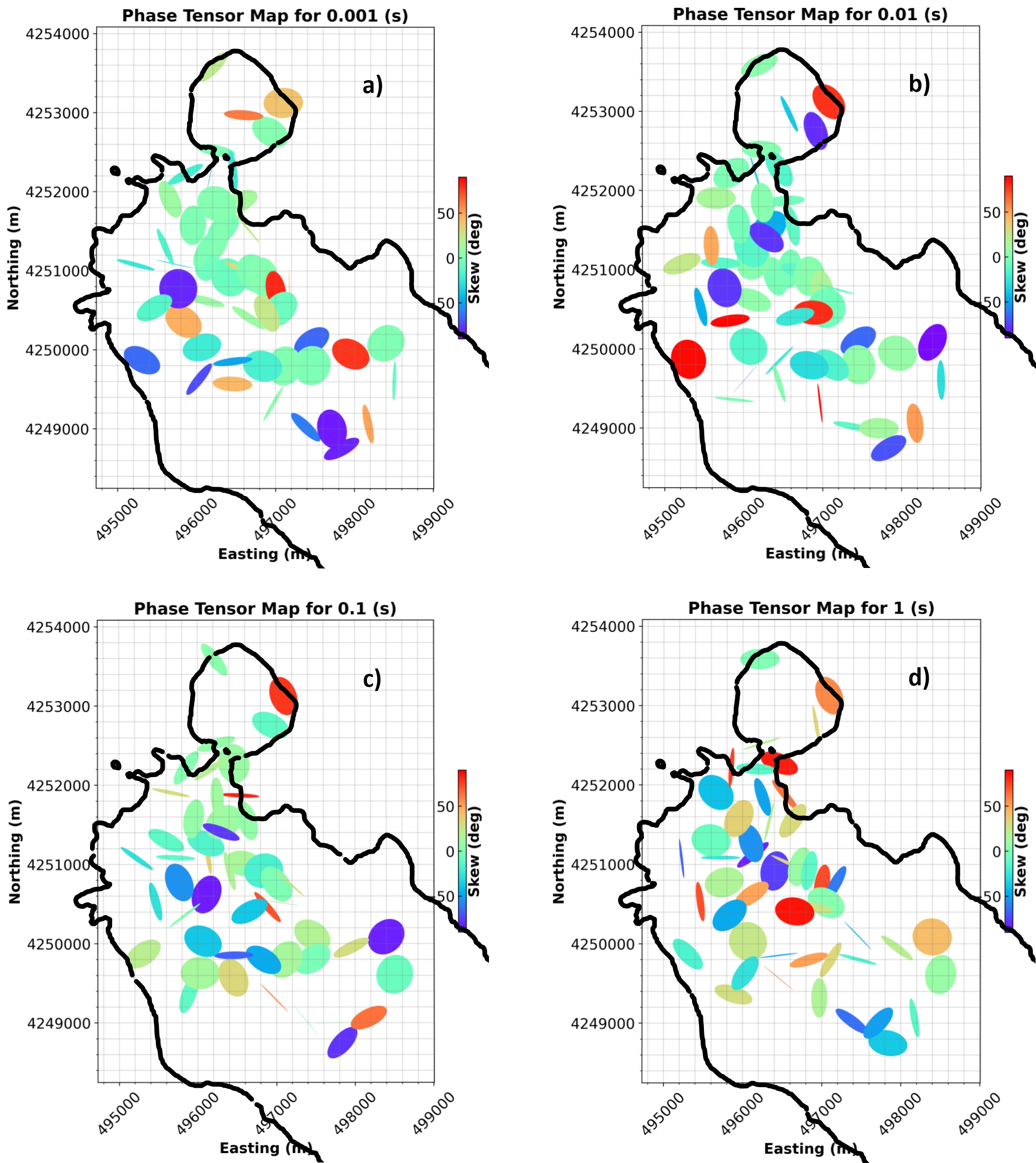

Supplement: Supplementary file 2 — Supplementary Figure S1. [file 41598_2023_43828_MOESM2_ESM.jpg]

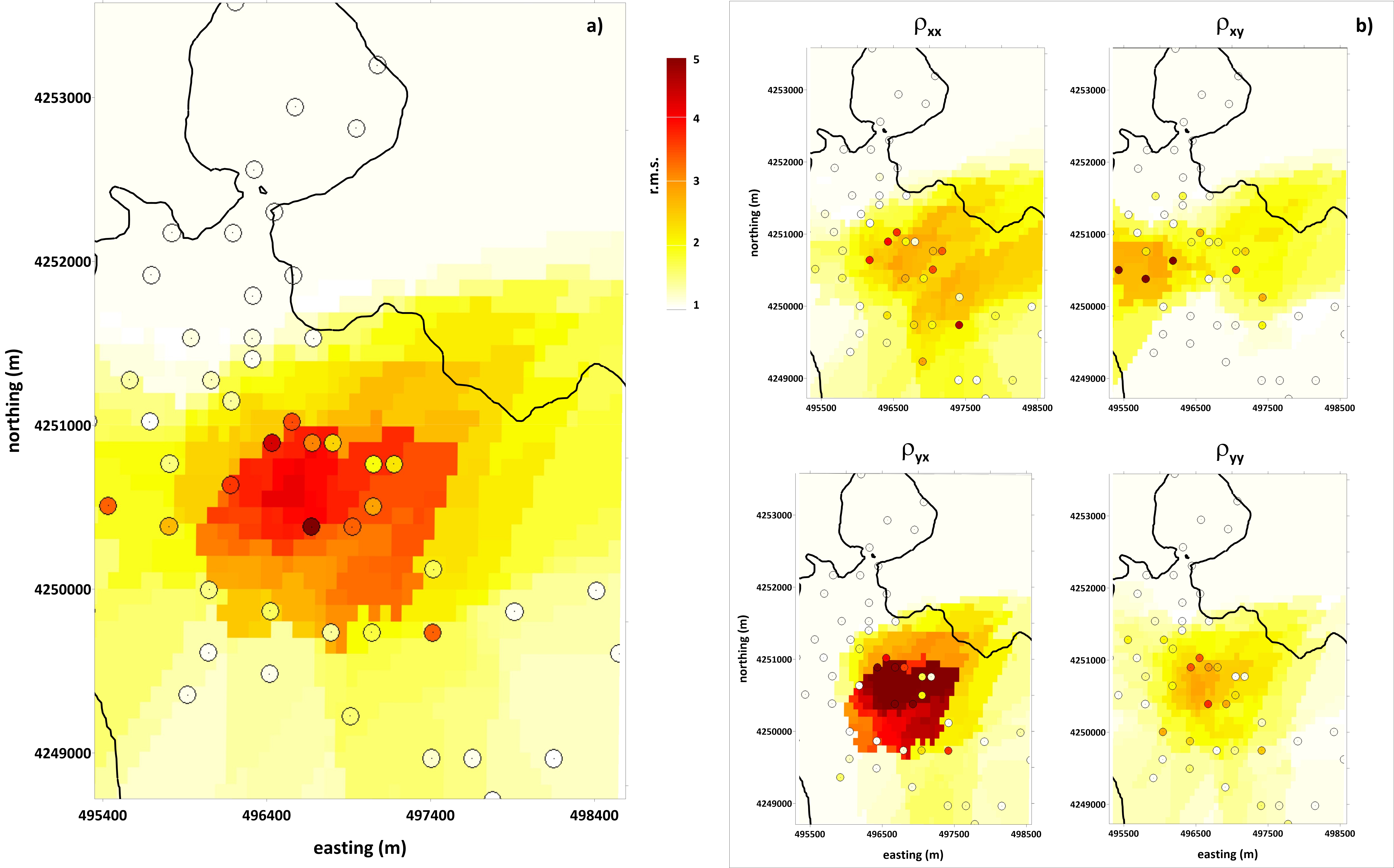

Supplement: Supplementary file 4 — Supplementary Figure S3. [file 41598_2023_43828_MOESM4_ESM.jpg]

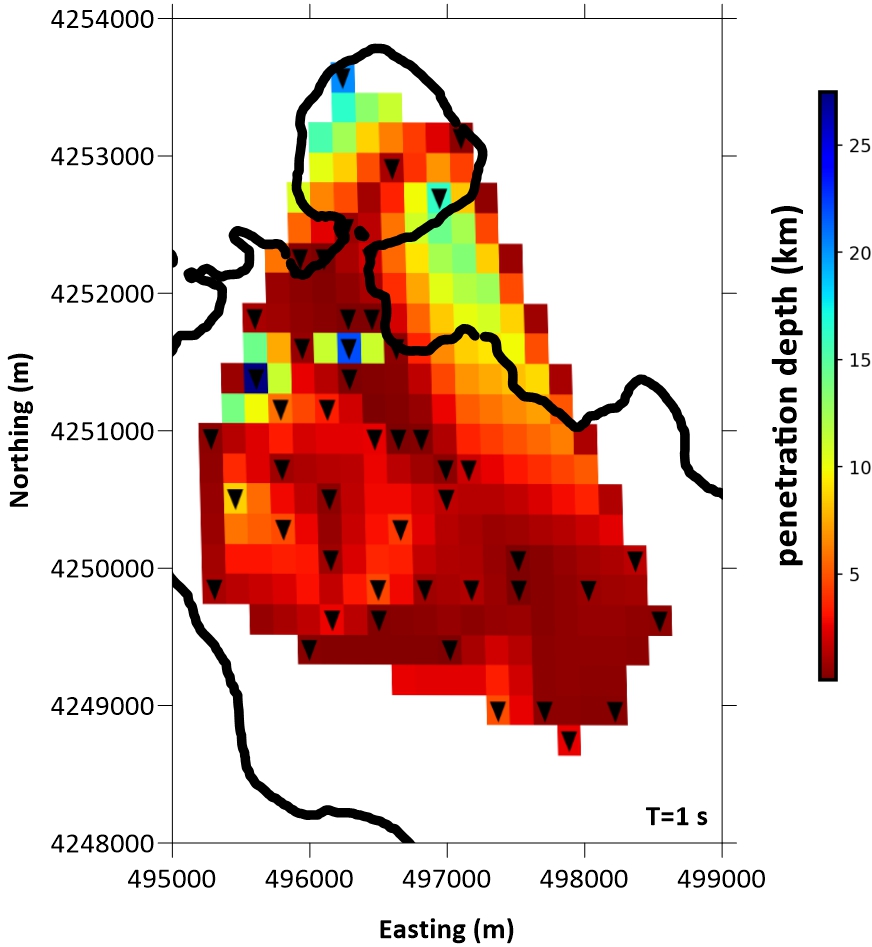

Supplement: Supplementary file 6 — Supplementary Figure S5. [file 41598_2023_43828_MOESM6_ESM.jpg]

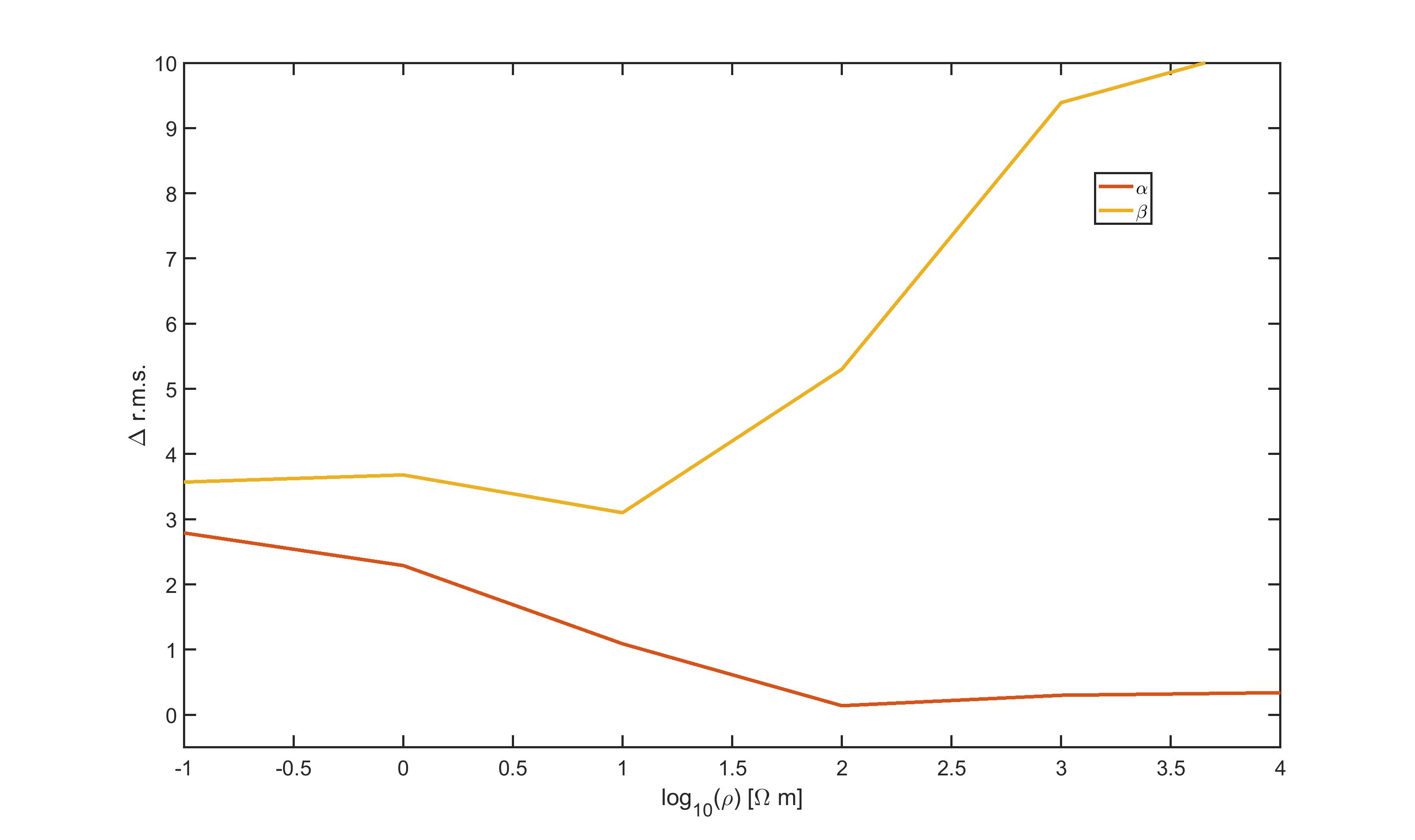

Supplement: Supplementary file 7 — Supplementary Figure S6. [file 41598_2023_43828_MOESM7_ESM.jpg]
