# Supplementary material for: Three-dimensional magnetotelluric modeling of Vulcano Island (Eolie, Italy) and its implications for understanding recent volcanic unrest
Source: Sci Rep. 2023 Sep 30;13:16458. doi: 10.1038/s41598-023-43828-x (PMC10543375; doi:10.1038/s41598-023-43828-x)
Supplement: Supplementary file 5 — Supplementary Figure S4. [file 41598_2023_43828_MOESM5_ESM.pdf]

Site: vulc01

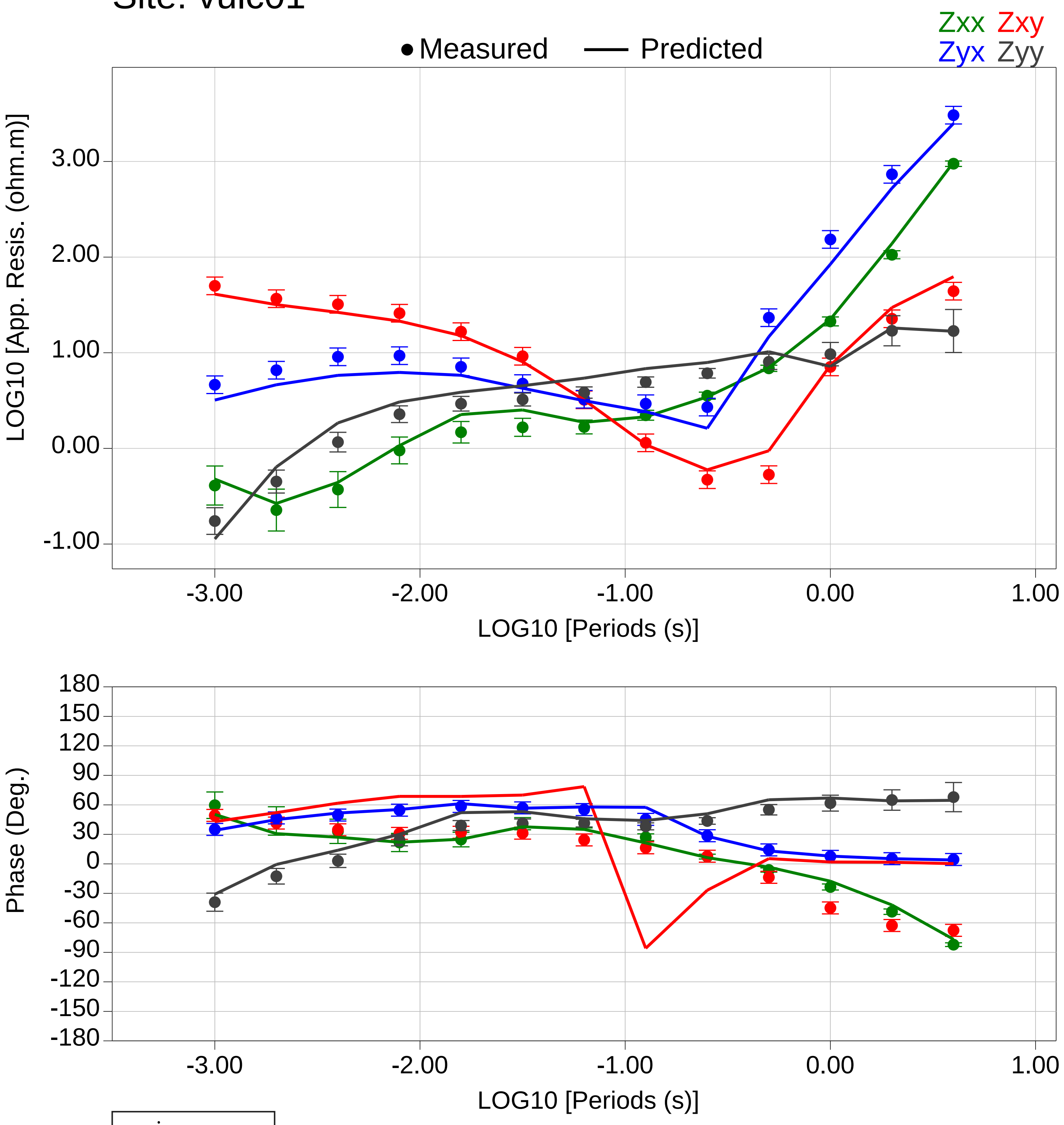

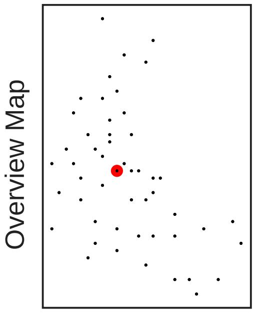

Overall RMS (Z+Tz)= 2.13
Total Z RMS = 2.13
Zxx RMS = 0.95
Zxy RMS = 3.88
Zyx RMS = 0.81
Zyy RMS = 1.26

Site: vulc02

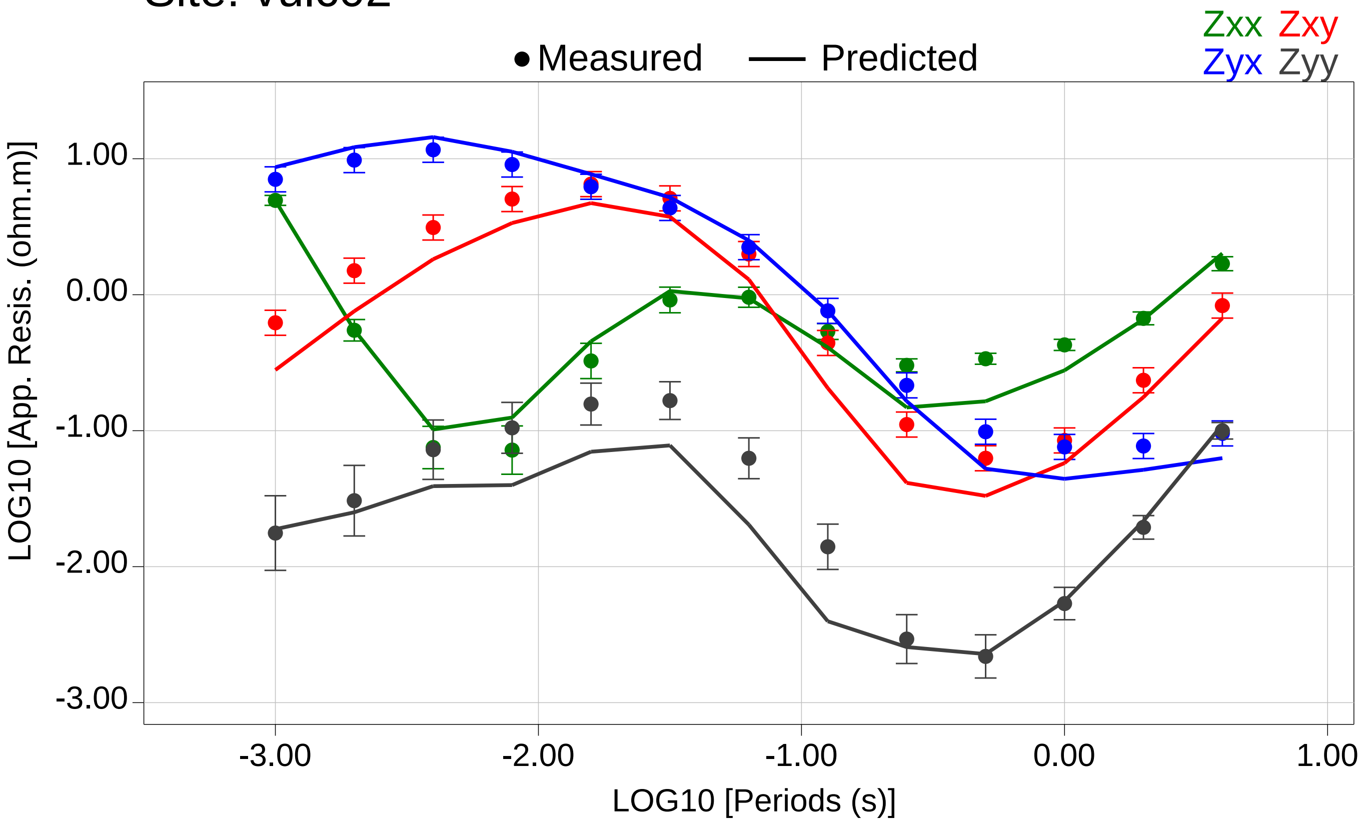

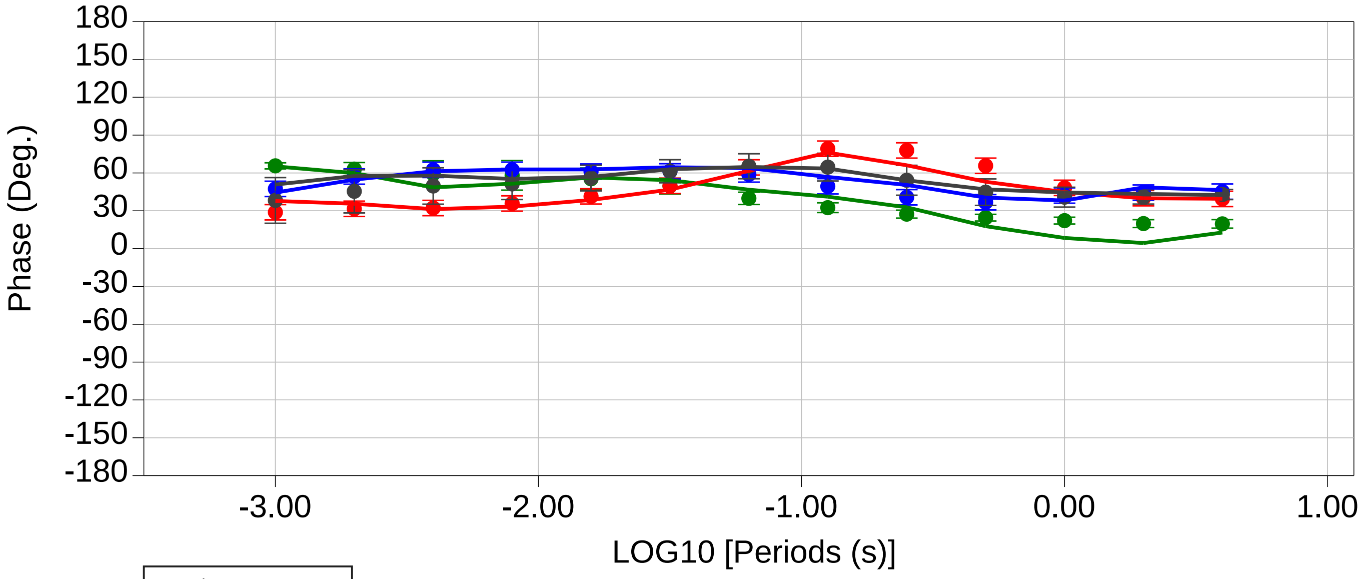

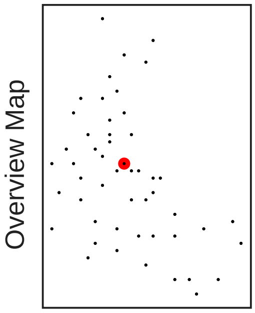

Overall RMS (Z+Tz)= 1.20
Total Z RMS = 1.20
Zxx RMS = 1.78
Zxy RMS = 1.20
Zyx RMS = 0.79
Zyy RMS = 0.72

Site: vulc03

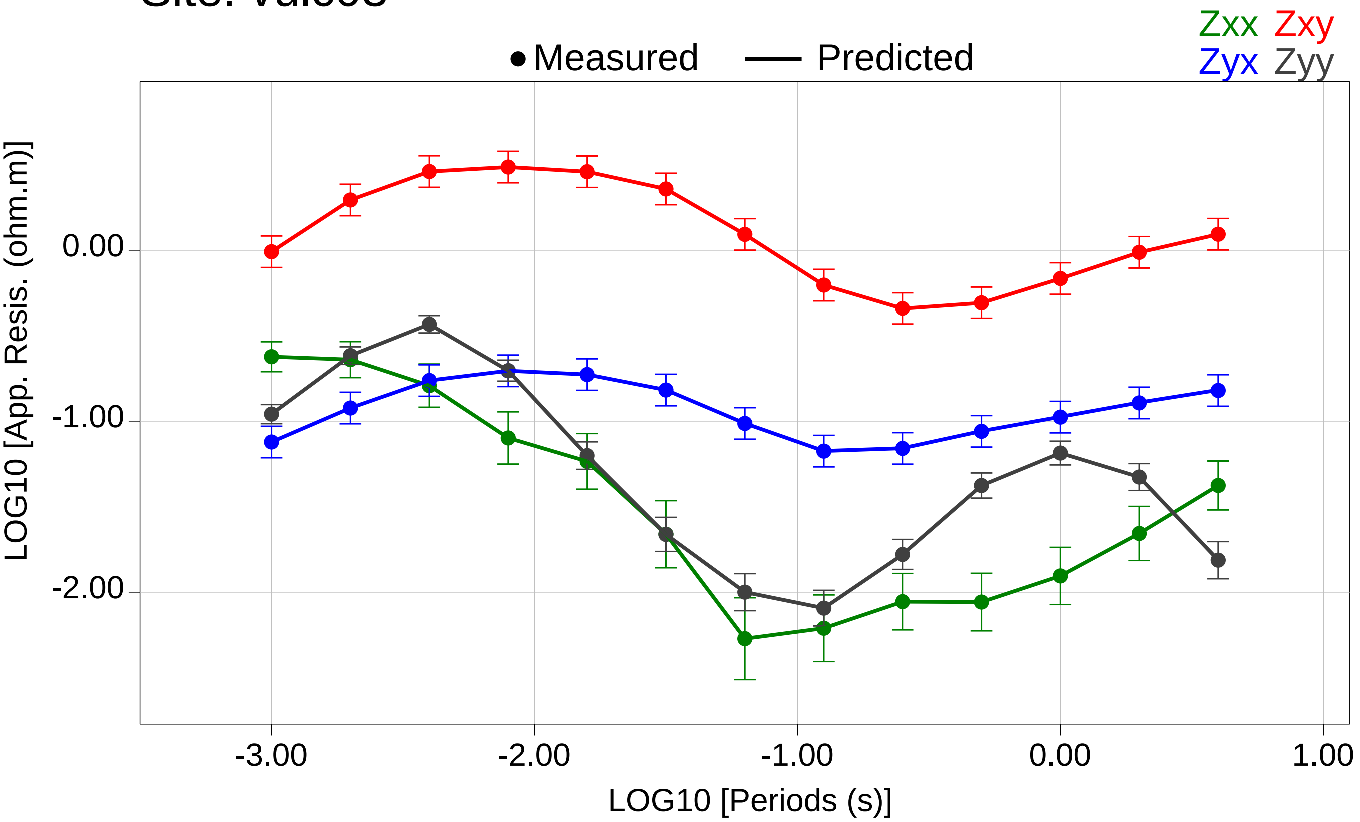

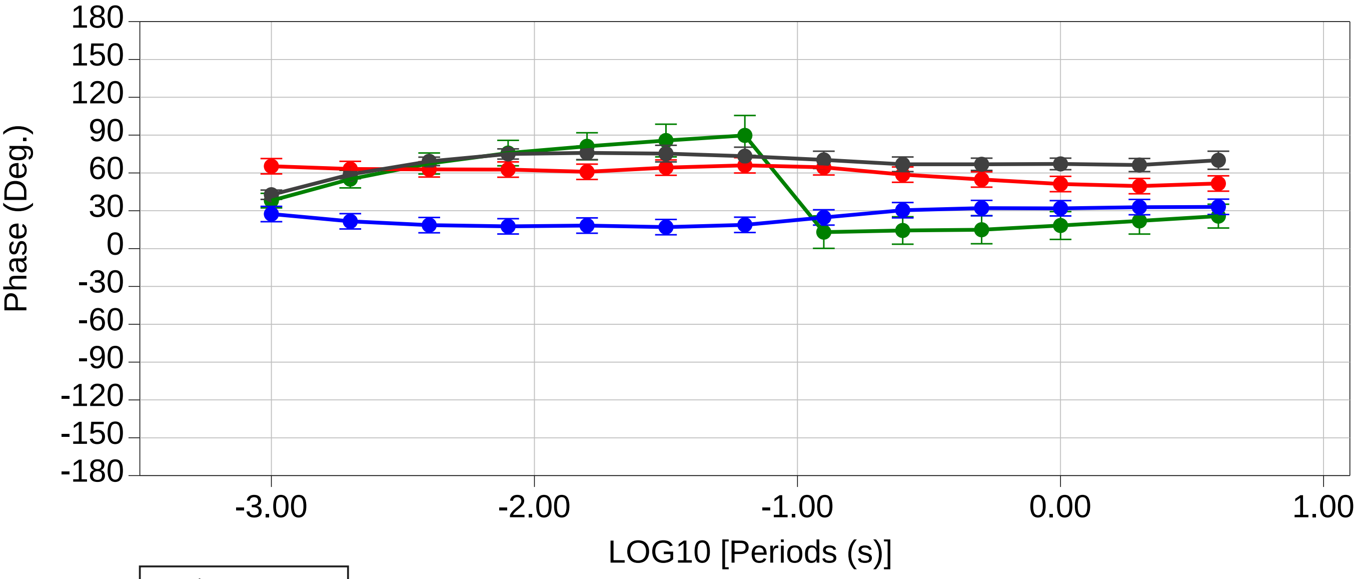

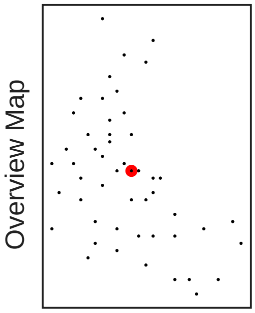

Overall RMS (Z+Tz)= 0.00
Total Z RMS = 0.00
Zxx RMS = 0.00
Zxy RMS = 0.00
Zyx RMS = 0.01
Zyy RMS = 0.00

Site: vulc04

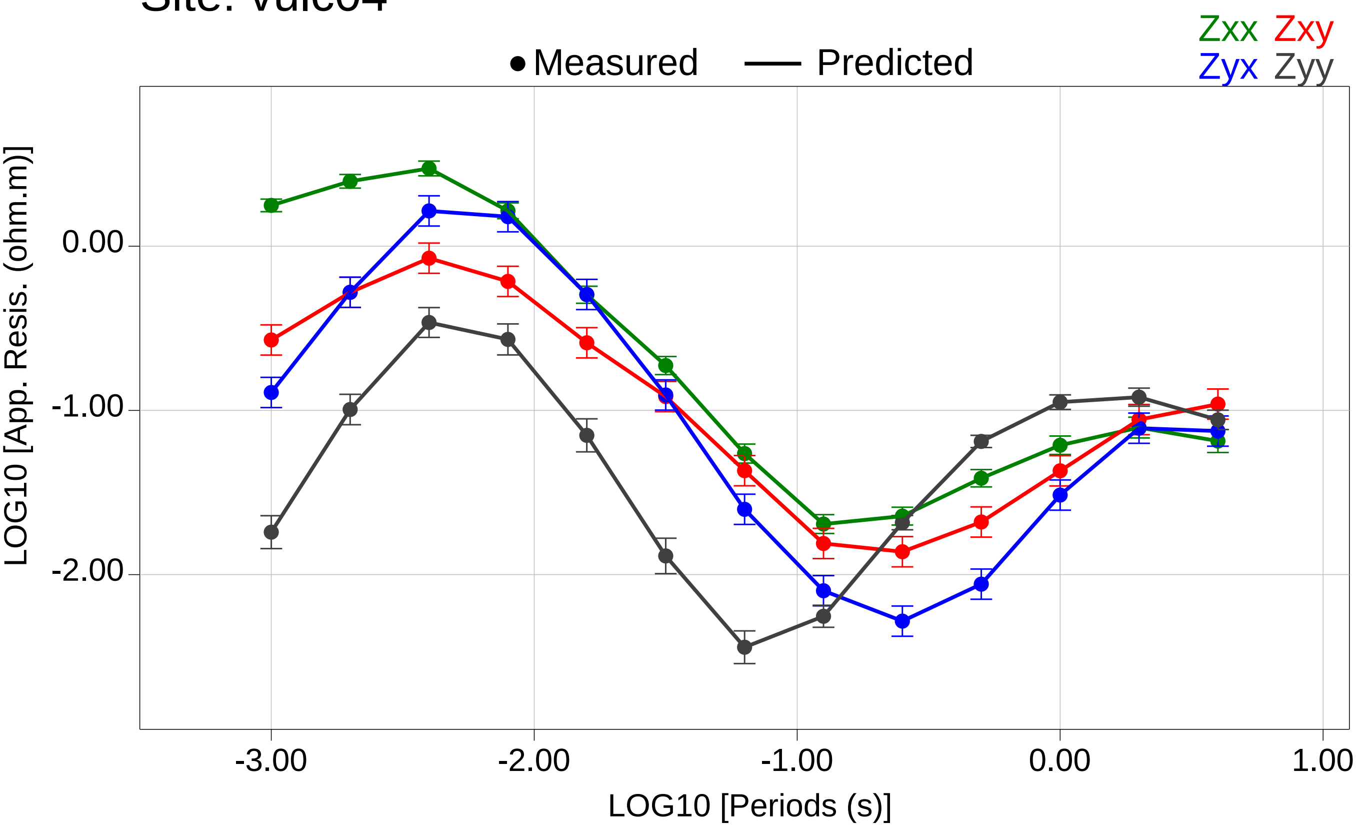

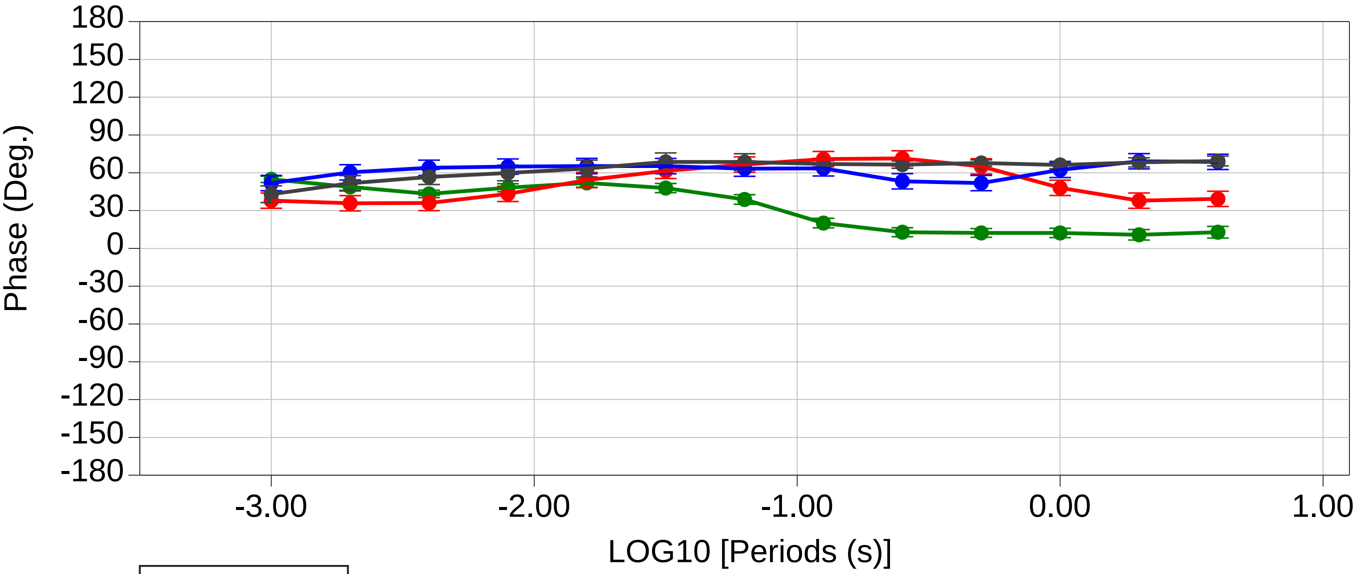

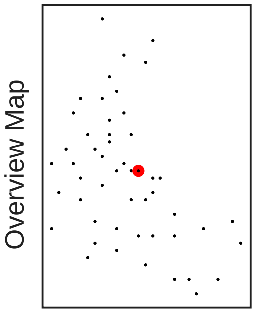

Overall RMS (Z+Tz)= 0.00
Total Z RMS = 0.00
Zxx RMS = 0.00
Zxy RMS = 0.00
Zyx RMS = 0.00
Zyy RMS = 0.00

Site: vulc05

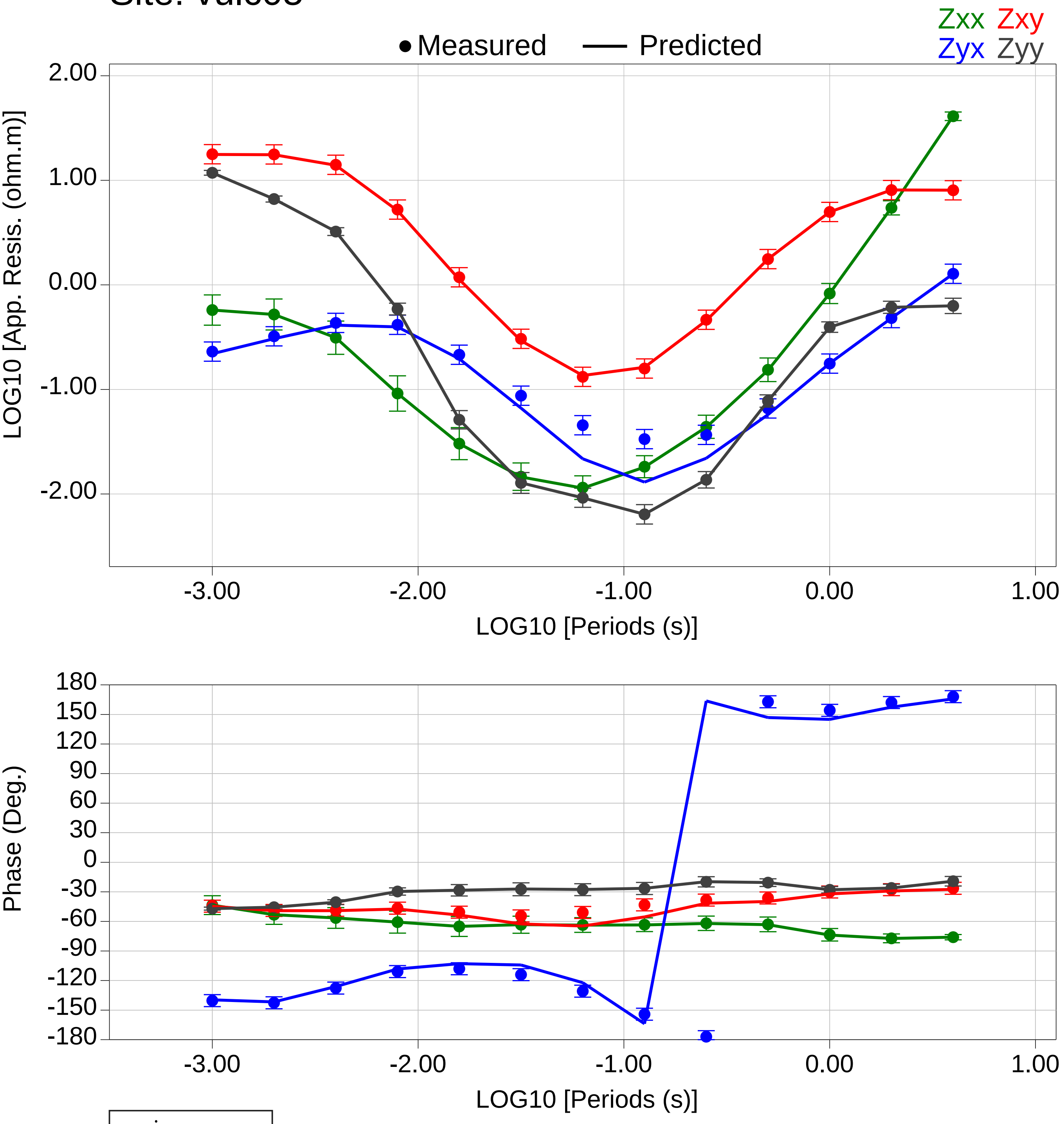

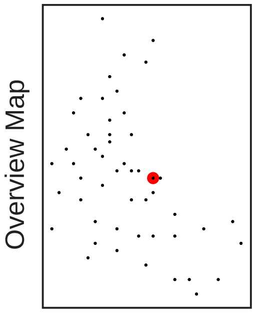

Overall RMS (Z+Tz)= 0.55
Total Z RMS = 0.55
Zxx RMS = 0.02
Zxy RMS = 0.48
Zyx RMS = 0.99
Zyy RMS = 0.01

Site: vulc06

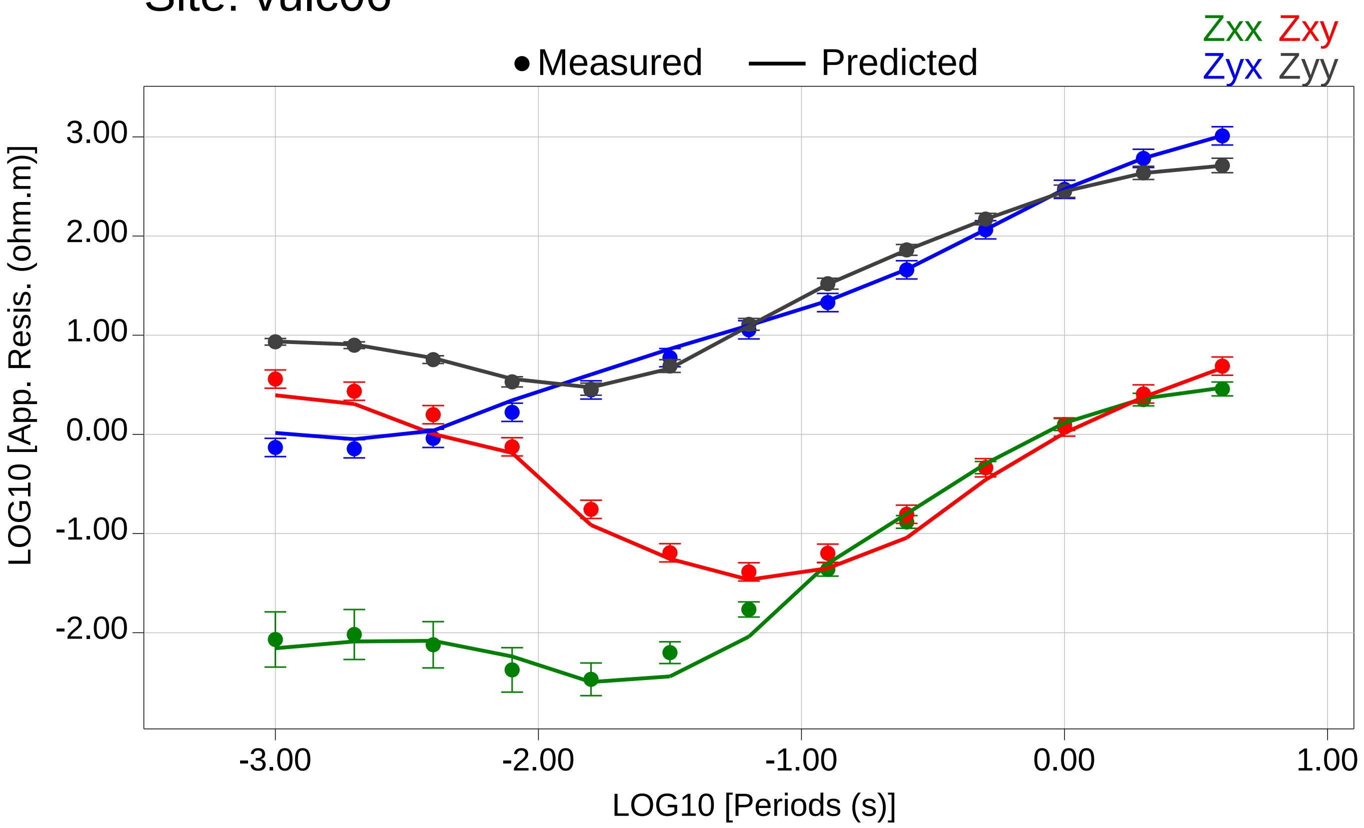

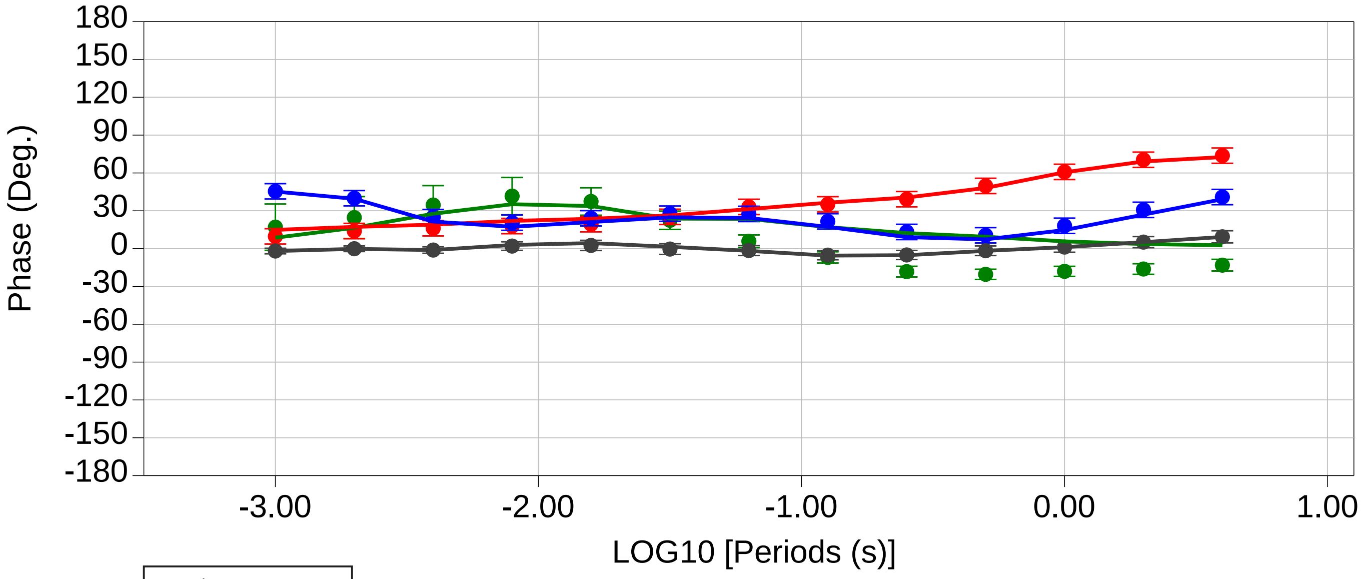

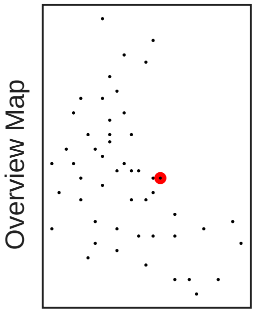

Overall RMS (Z+Tz)= 1.16
Total Z RMS = 1.16
Zxx RMS = 2.14
Zxy RMS = 0.67
Zyx RMS = 0.54
Zyy RMS = 0.16

Site: vulc07

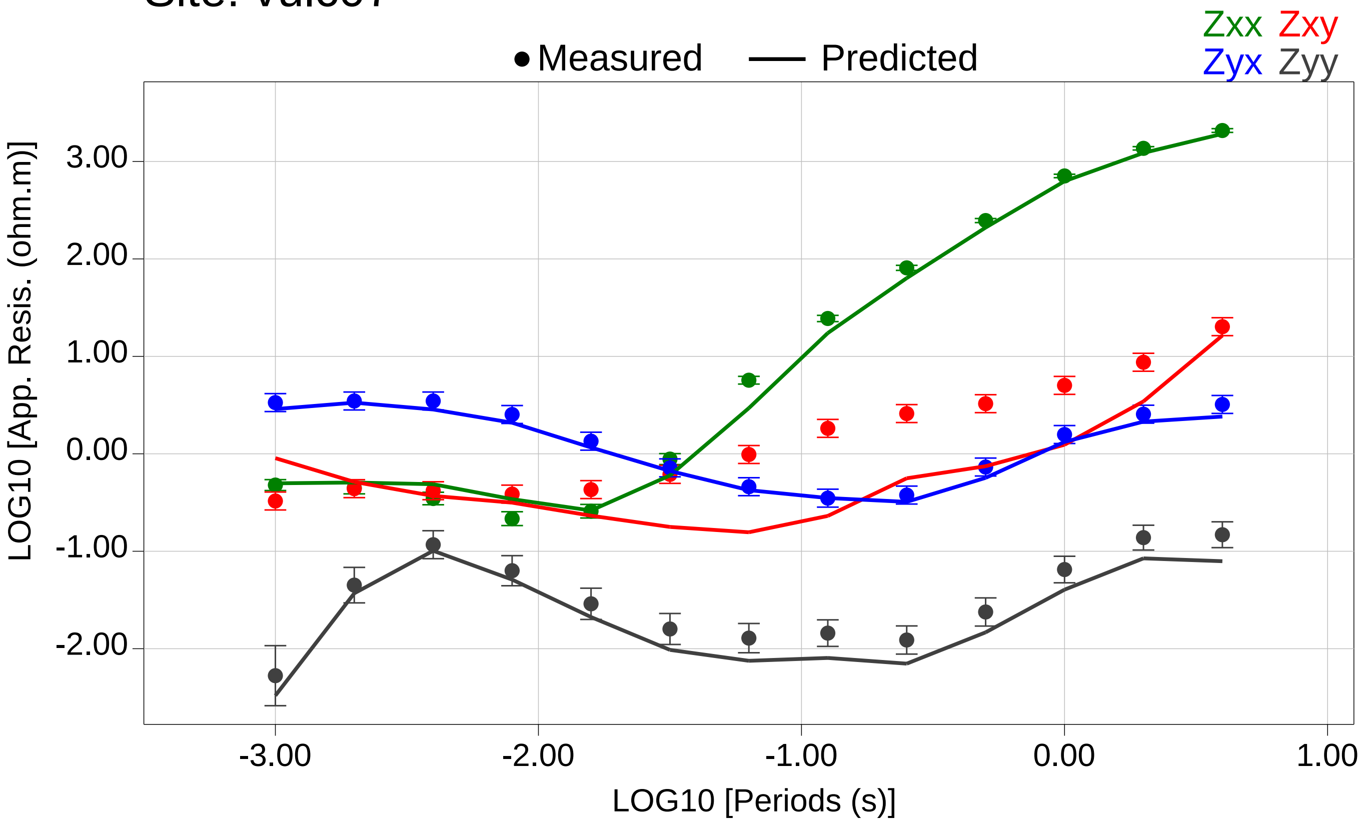

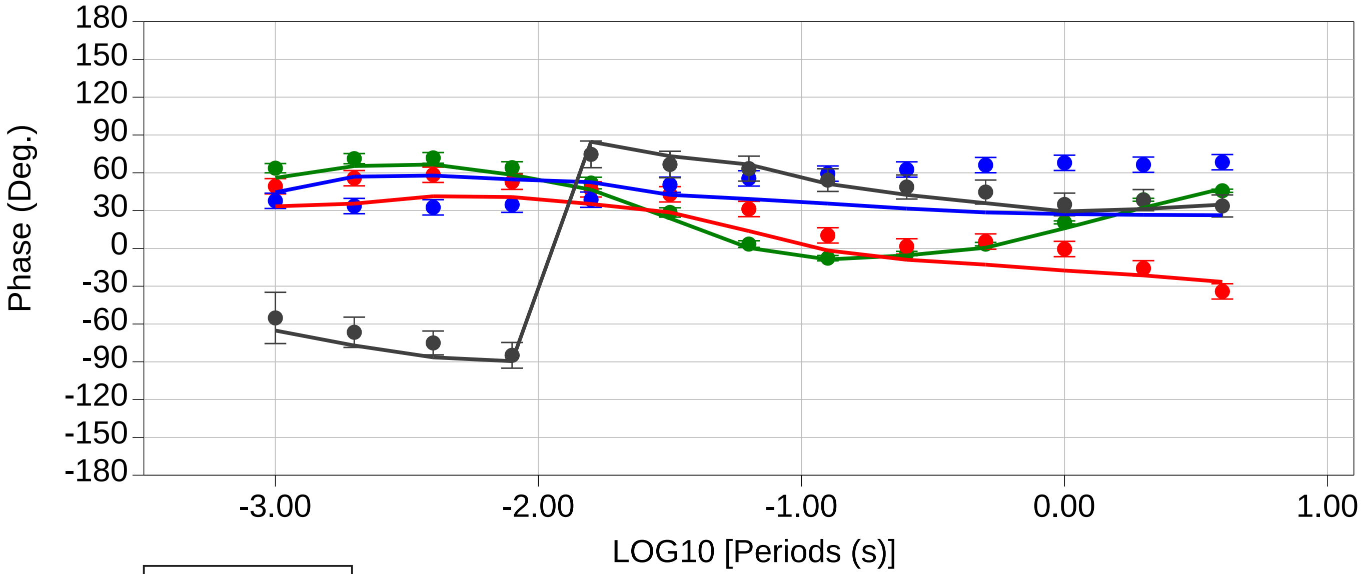

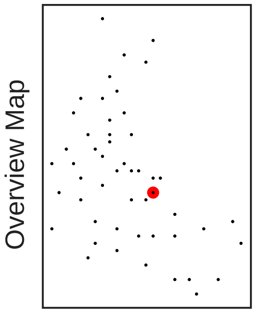

Overall RMS (Z+Tz)= 1.88
Total Z RMS = 1.88
Zxx RMS = 1.91
Zxy RMS = 2.30
Zyx RMS = 2.19
Zyy RMS = 0.68

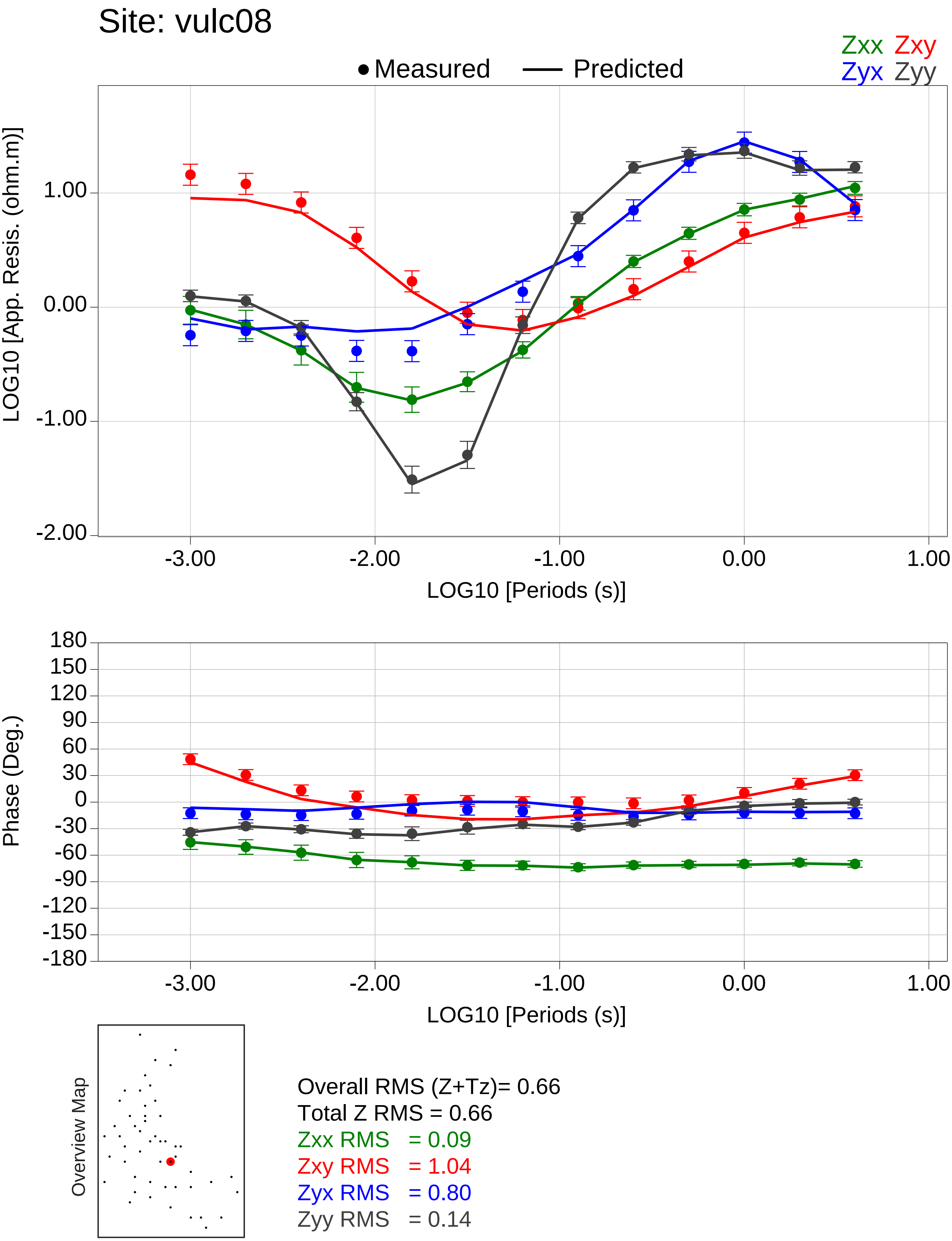

Overall RMS (Z+Tz)= 0.66
Total Z RMS = 0.66
Zxx RMS = 0.09
Zxy RMS = 1.04
Zyx RMS = 0.80
Zyy RMS = 0.14

Site: vulc09

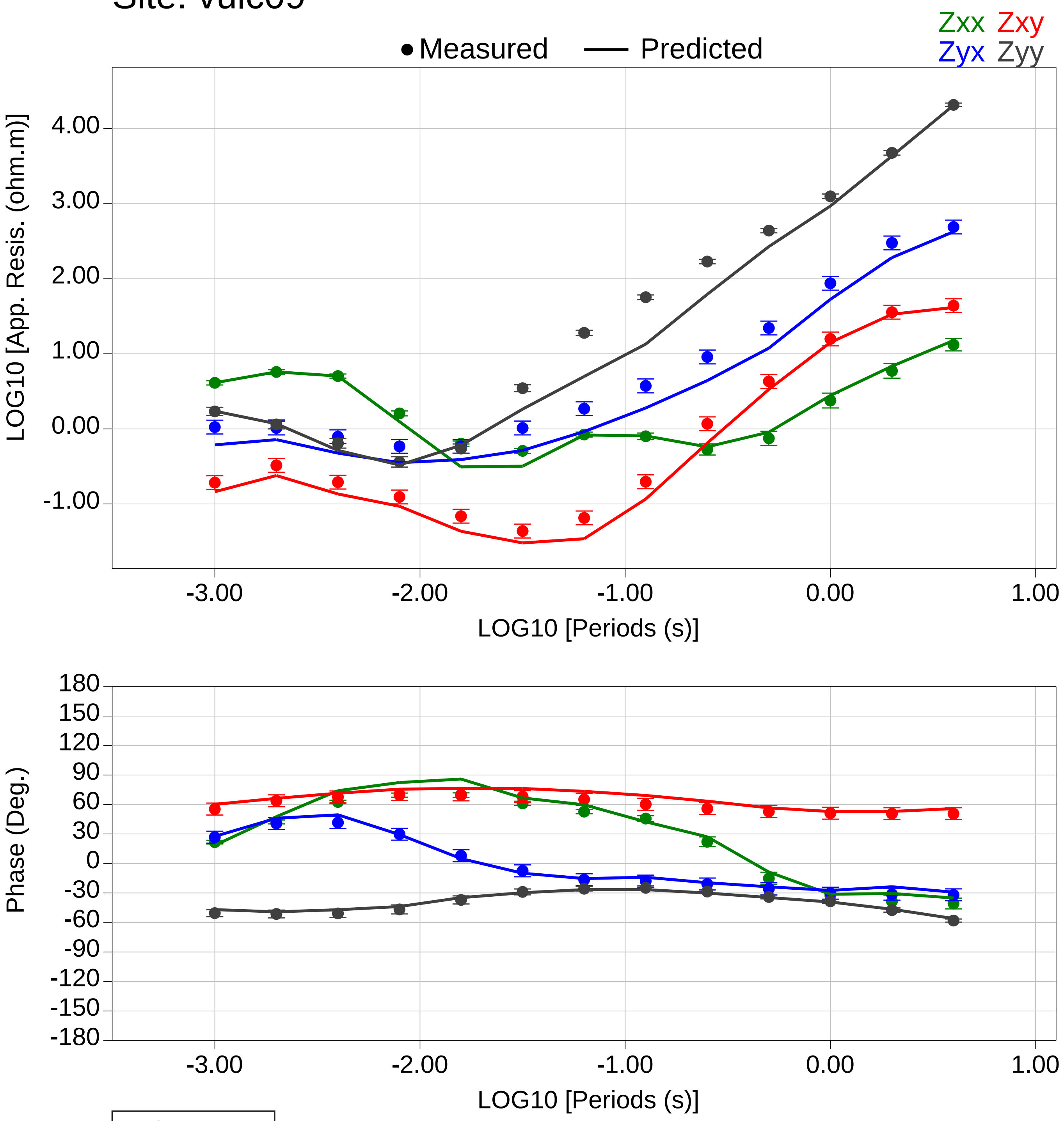

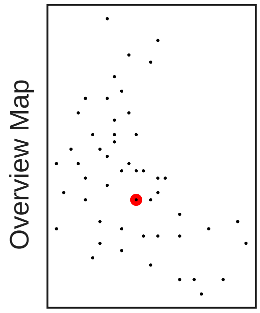

Overall RMS (Z+Tz)= 2.12
Total Z RMS = 2.12
Zxx RMS = 2.13
Zxy RMS = 0.90
Zyx RMS = 1.15
Zyy RMS = 3.36

Site: vulc10

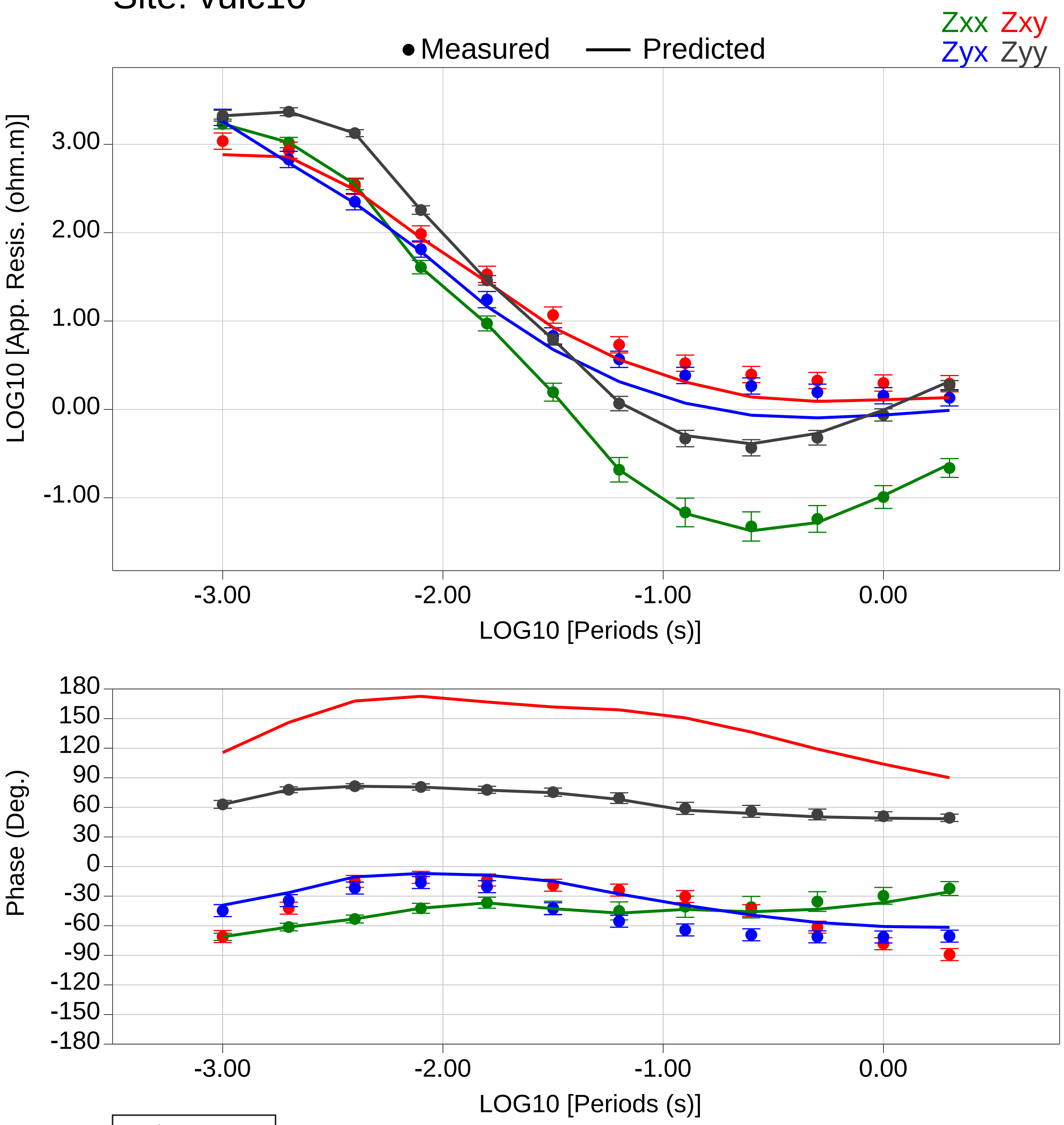

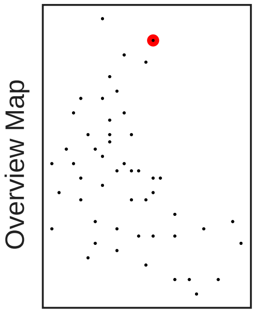

Overall RMS (Z+Tz)= 4.43
Total Z RMS = 4.43
Zxx RMS = 0.21
Zxy RMS = 8.71
Zyx RMS = 1.52
Zyy RMS = 0.25

Site: vulc11

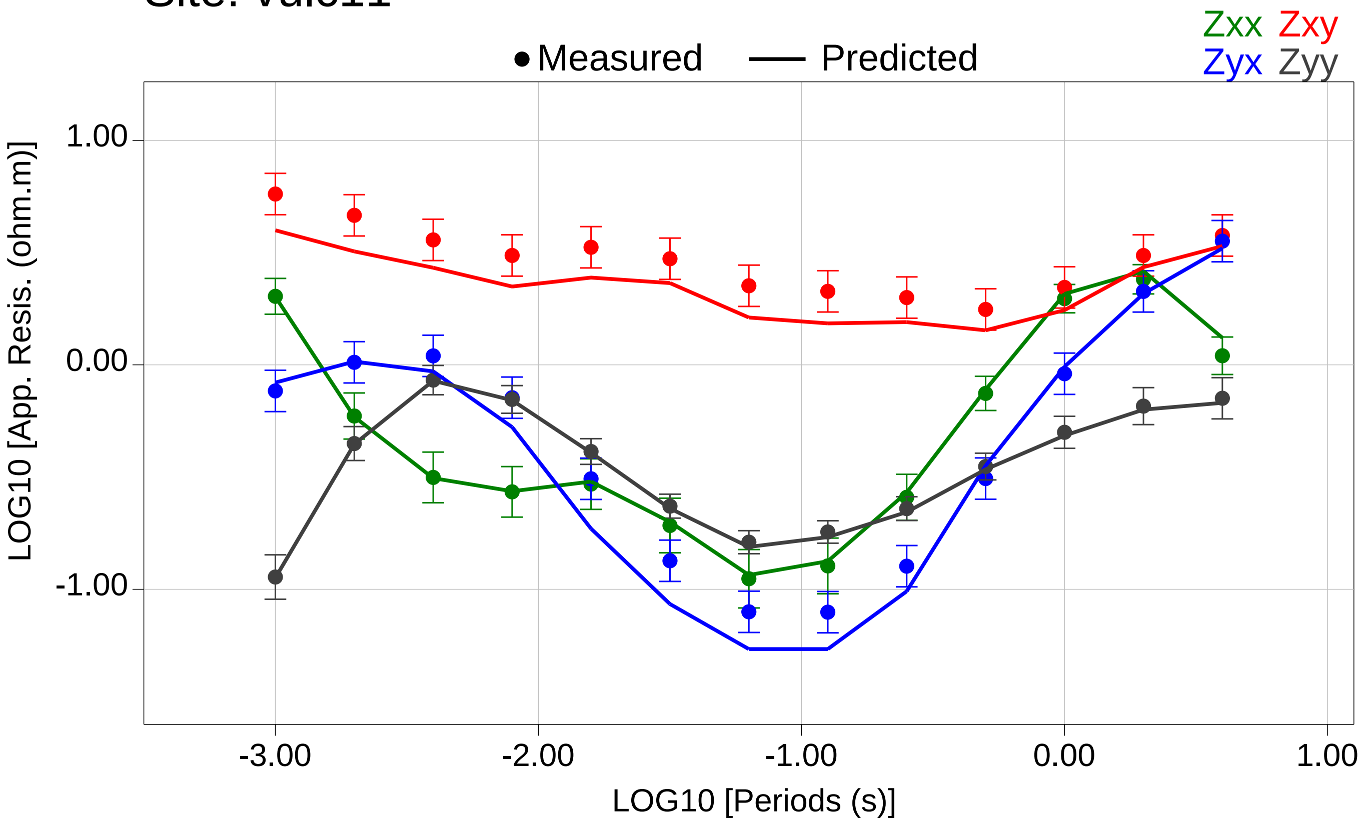

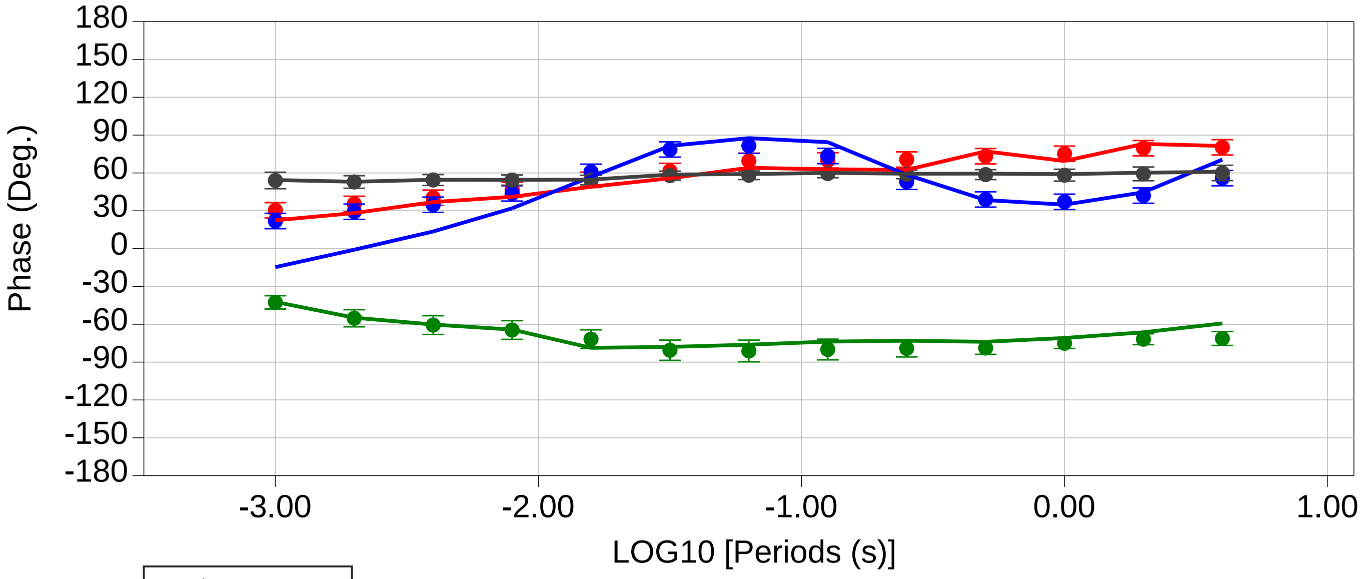

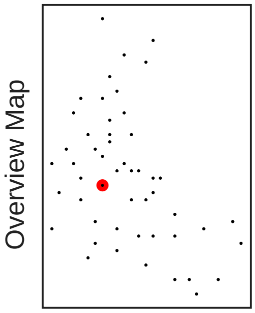

Overall RMS (Z+Tz)= 0.84
Total Z RMS = 0.84
Zxx RMS = 0.50
Zxy RMS = 0.75
Zyx RMS = 1.41
Zyy RMS = 0.14

Site: vulc12

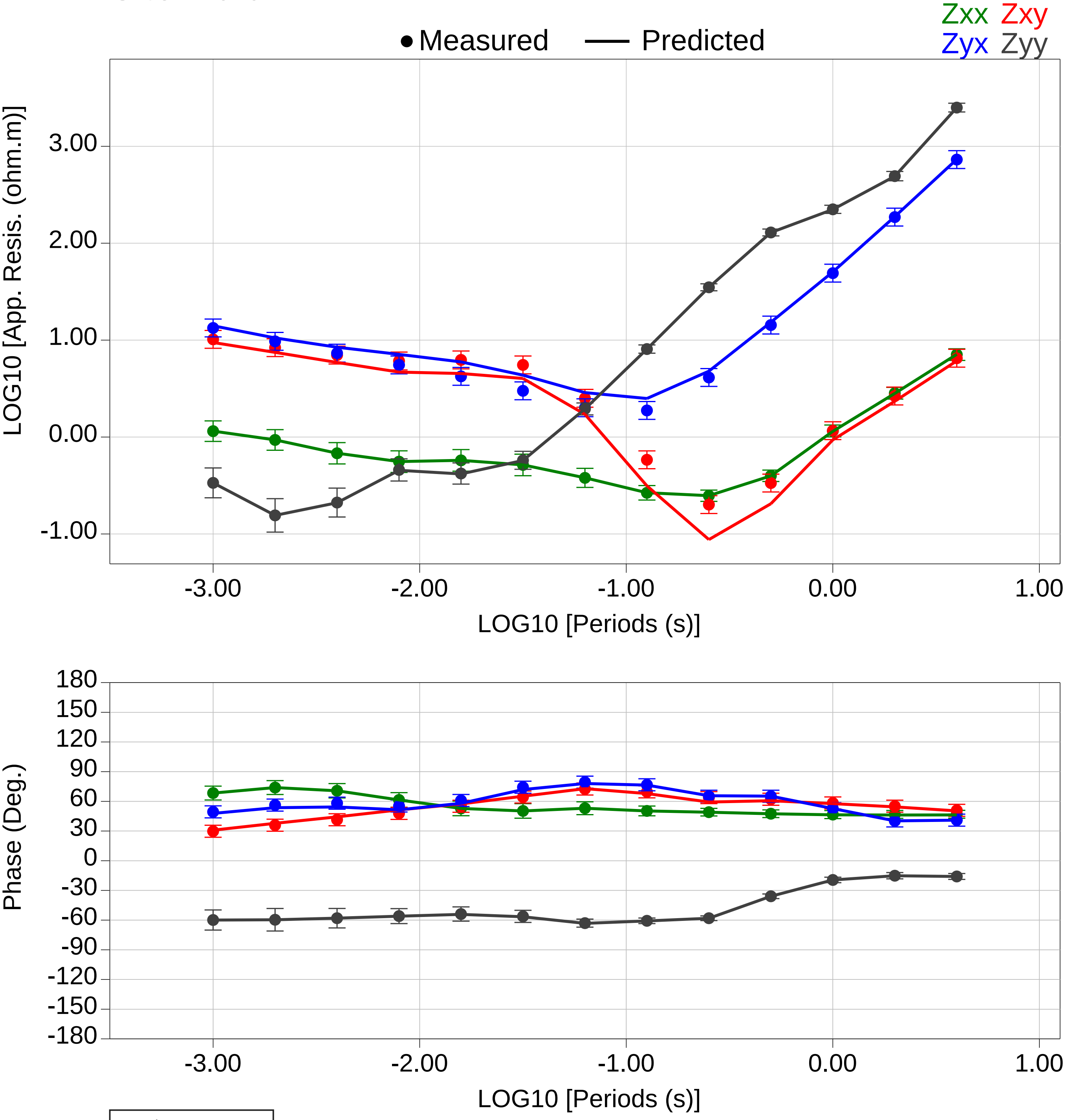

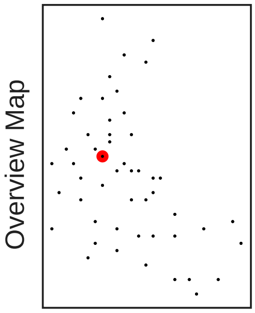

Overall RMS (Z+Tz)= 0.49
Total Z RMS = 0.49
Zxx RMS = 0.04
Zxy RMS = 0.79
Zyx RMS = 0.57
Zyy RMS = 0.02

Site: vulc13

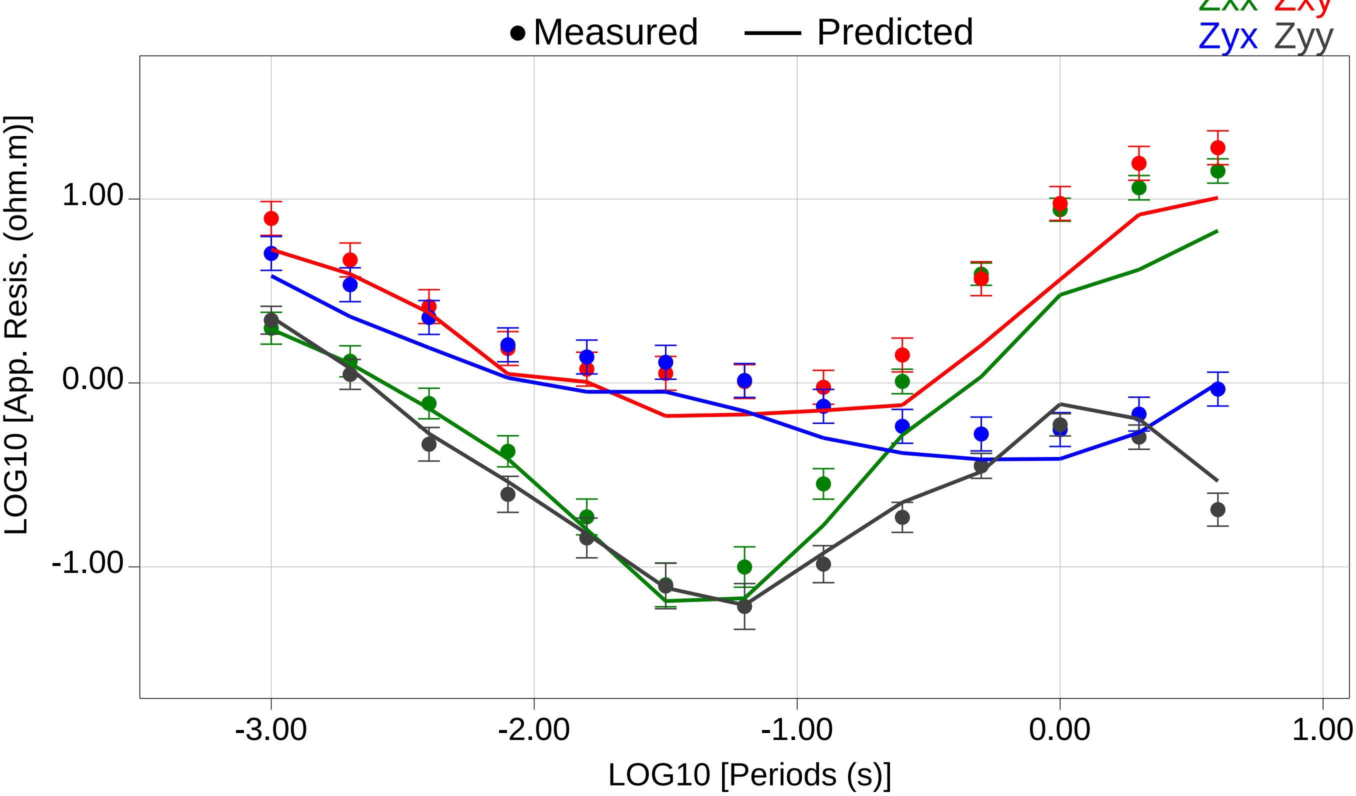

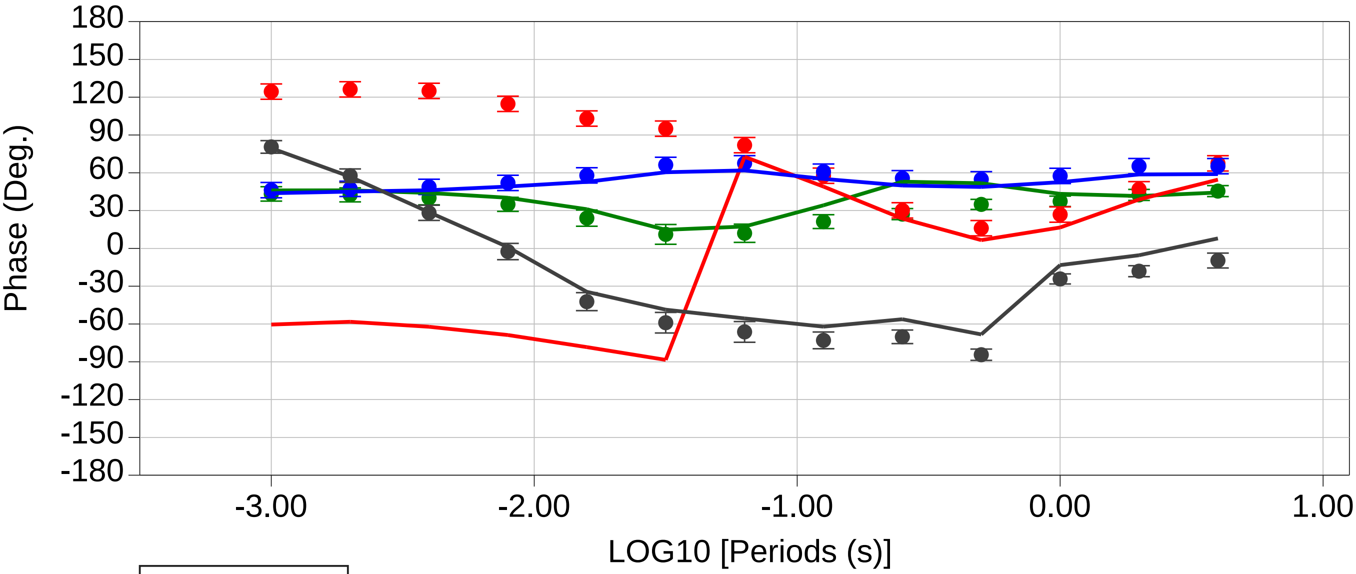

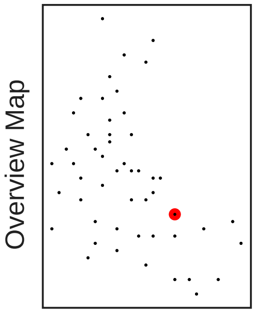

Overall RMS (Z+Tz)= 3.27
Total Z RMS = 3.27
Zxx RMS = 1.91
Zxy RMS = 6.10
Zyx RMS = 0.84
Zyy RMS = 1.15

Site: vulc14

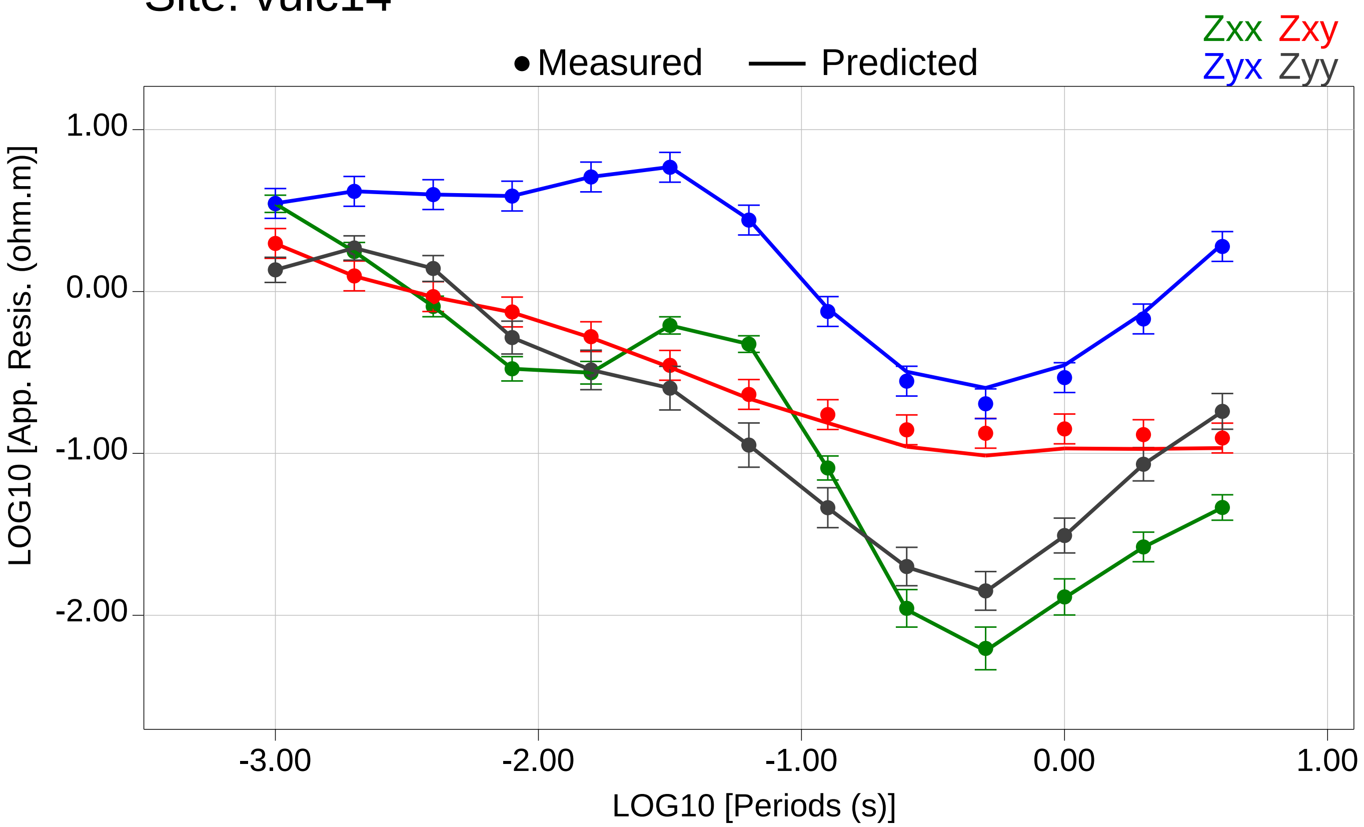

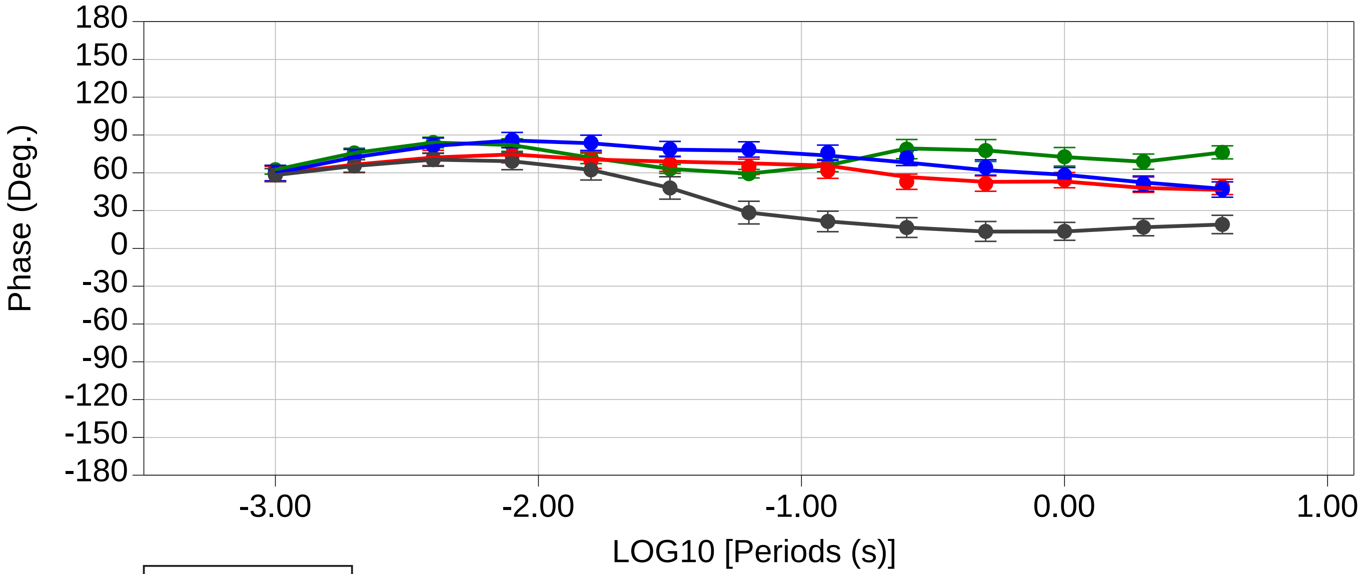

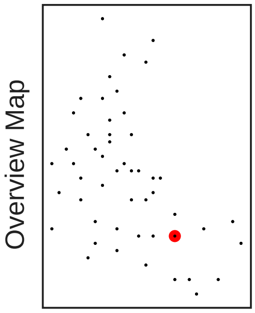

Overall RMS (Z+Tz)= 0.23
Total Z RMS = 0.23
Zxx RMS = 0.02
Zxy RMS = 0.38
Zyx RMS = 0.26
Zyy RMS = 0.01

Site: vulc15

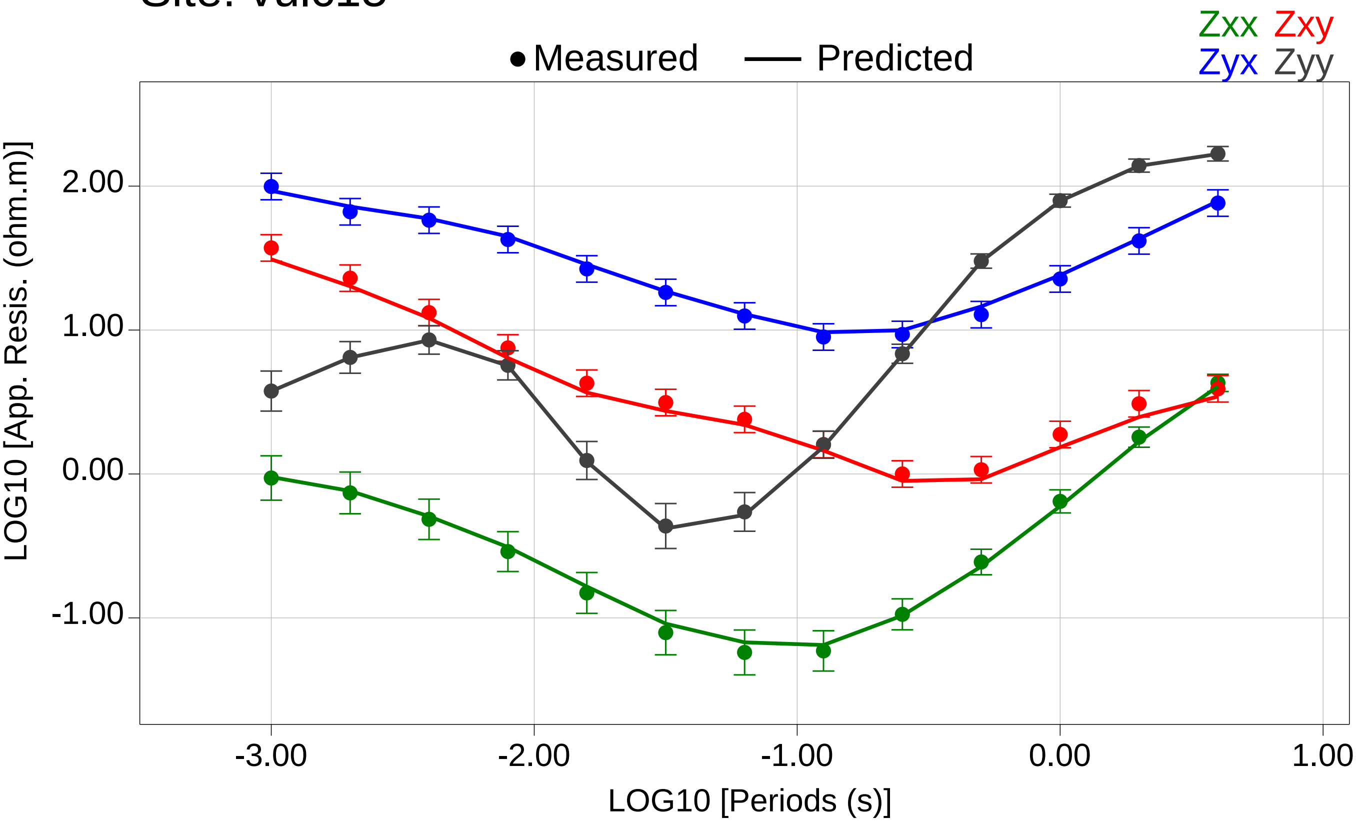

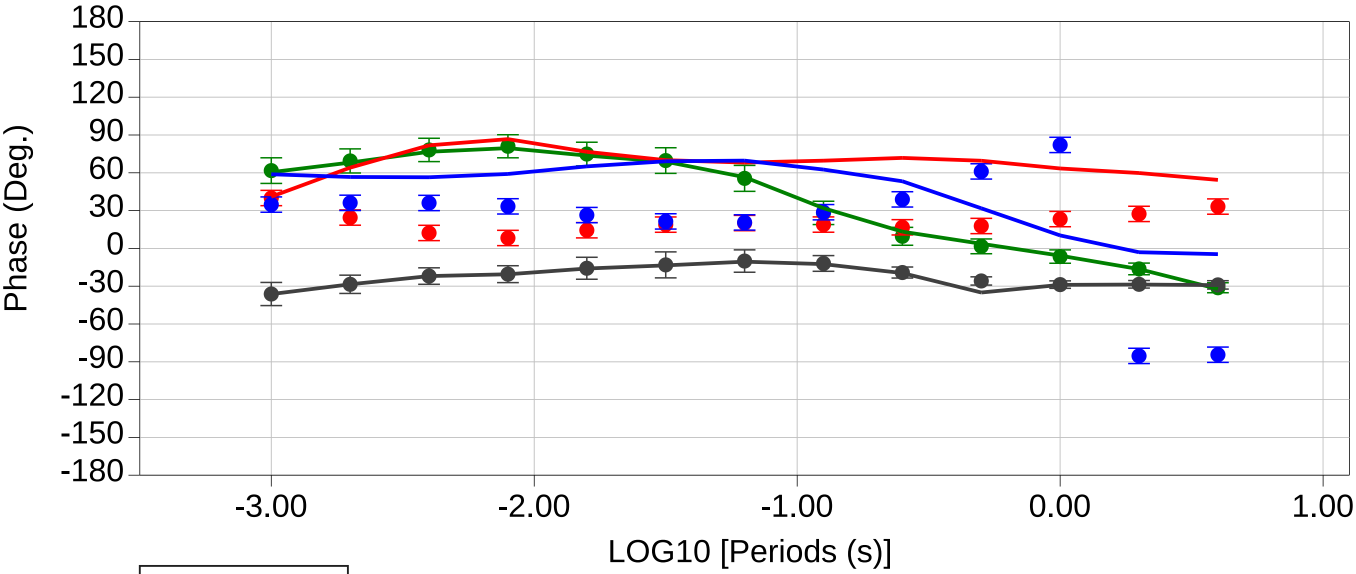

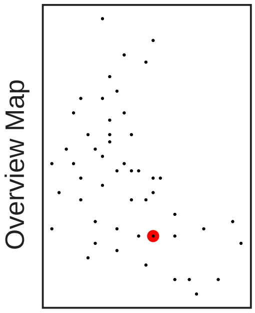

Overall RMS (Z+Tz)= 2.67
Total Z RMS = 2.67
Zxx RMS = 0.20
Zxy RMS = 3.83
Zyx RMS = 3.70
Zyy RMS = 0.39

Site: vulc16

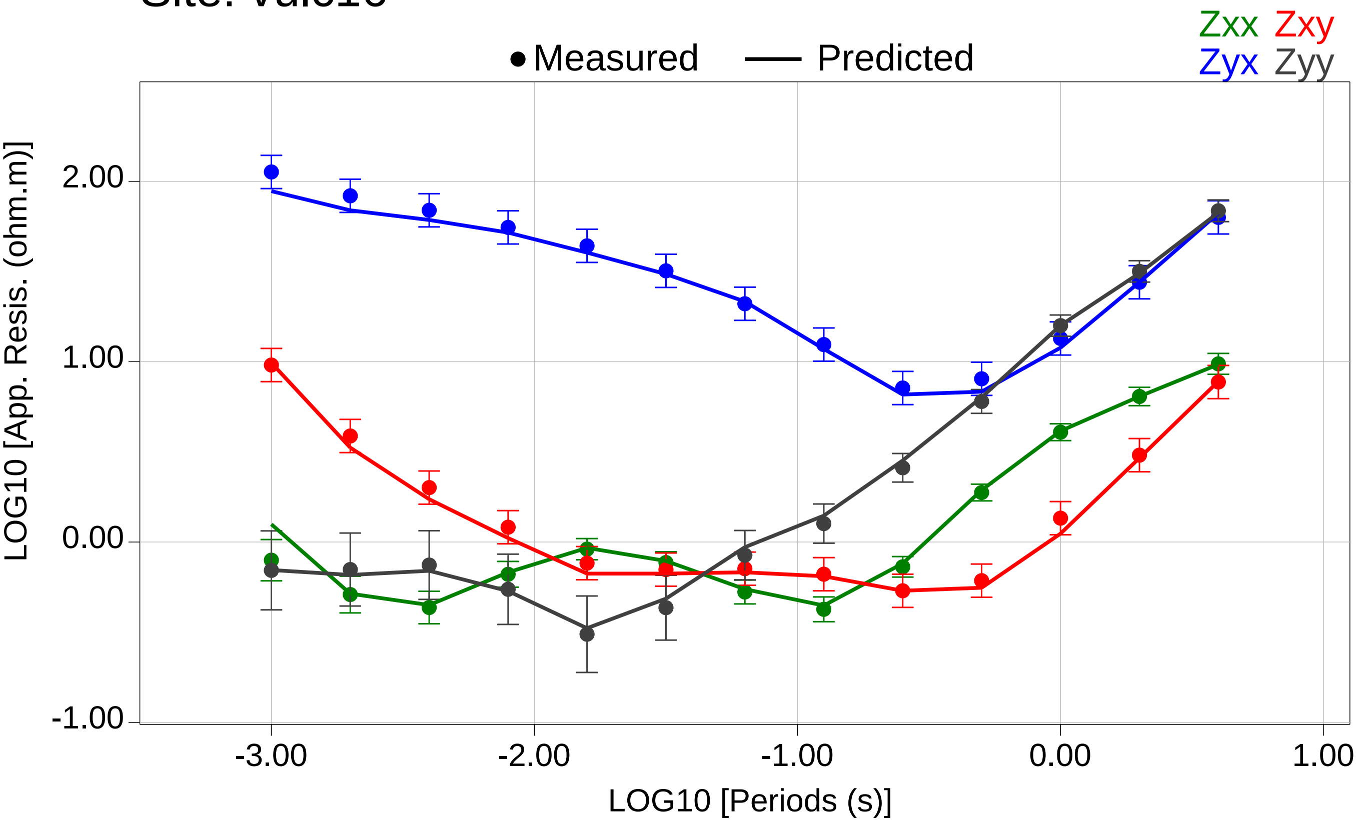

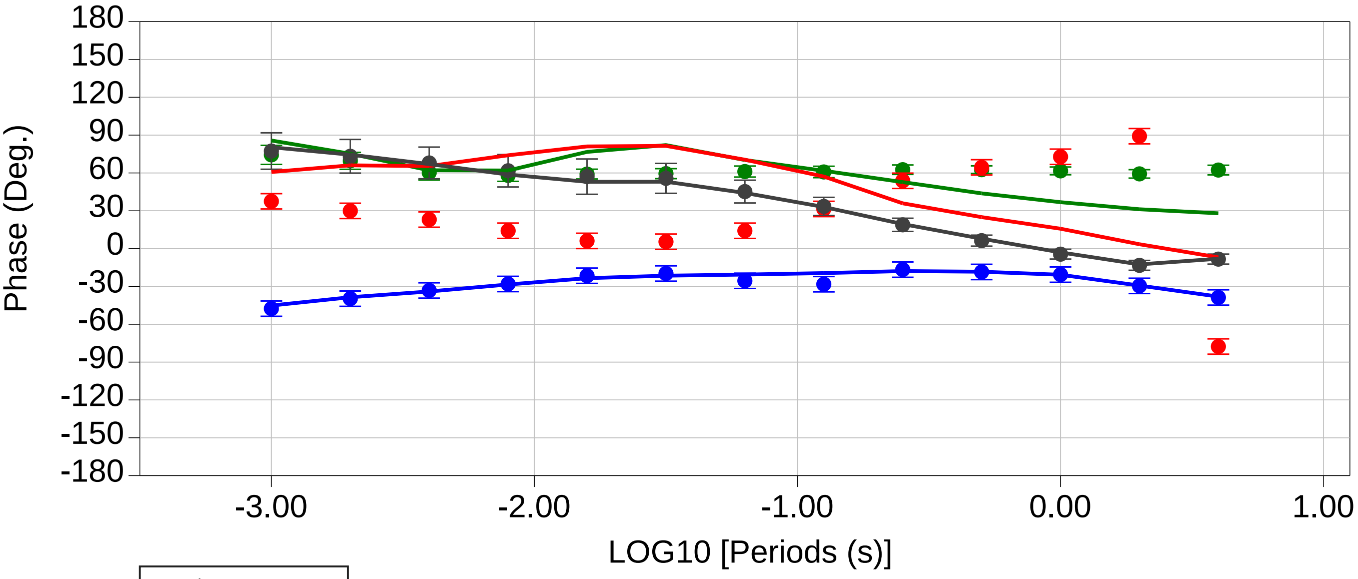

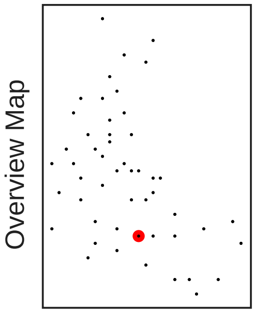

Overall RMS (Z+Tz)= 2.46
Total Z RMS = 2.46
Zxx RMS = 2.49
Zxy RMS = 4.22
Zyx RMS = 0.36
Zyy RMS = 0.17

Site: vulc17

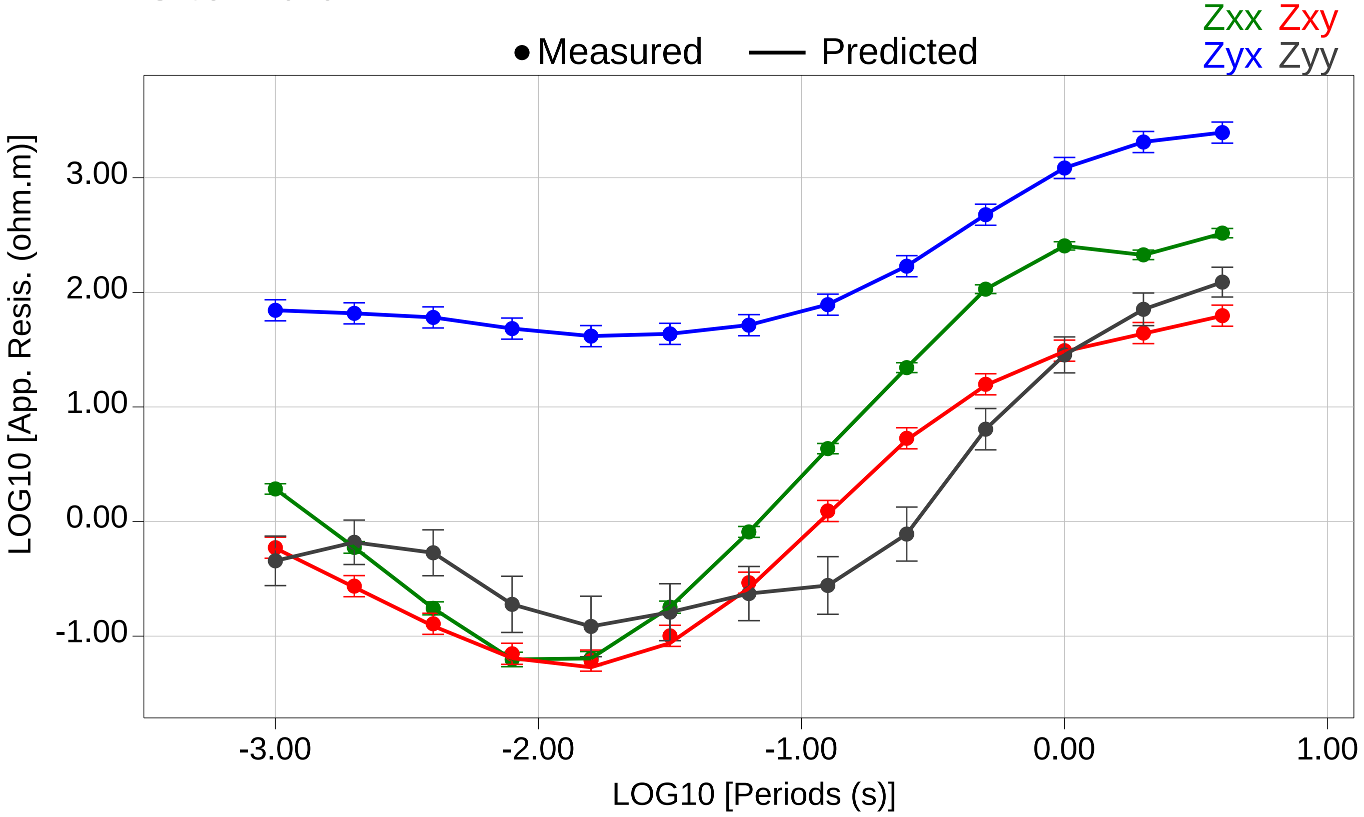

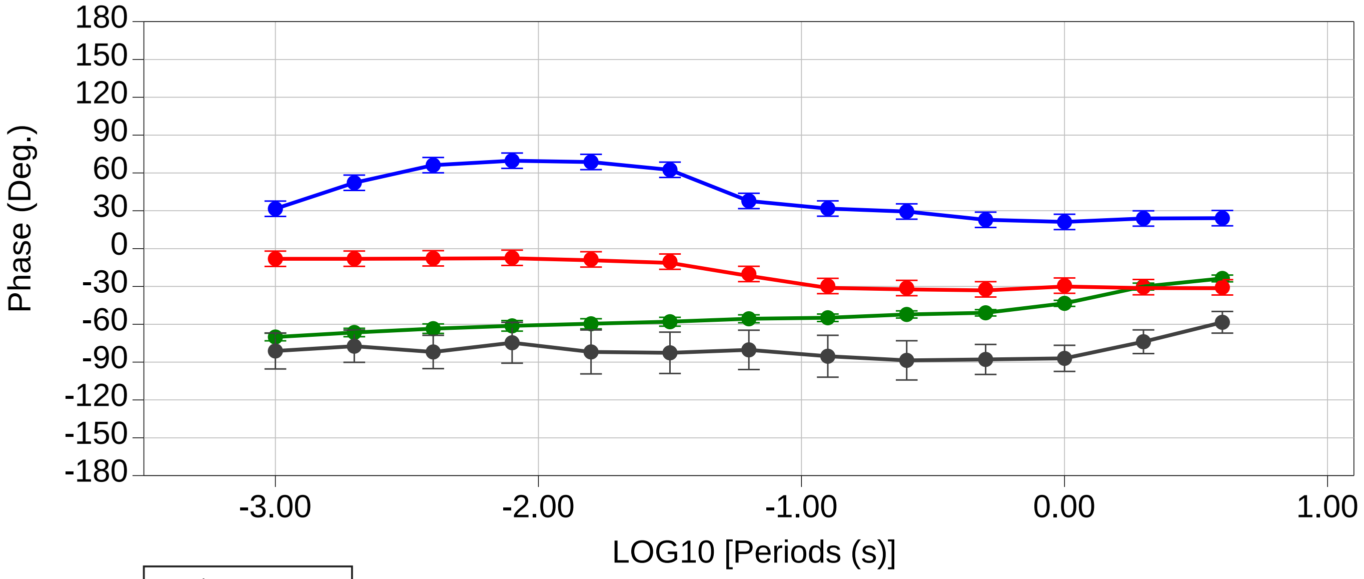

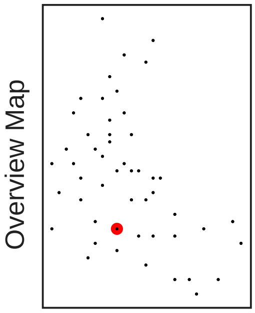

Overall RMS (Z+Tz)= 0.09
Total Z RMS = 0.09
Zxx RMS = 0.01
Zxy RMS = 0.18
Zyx RMS = 0.01
Zyy RMS = 0.00

Site: vulc18

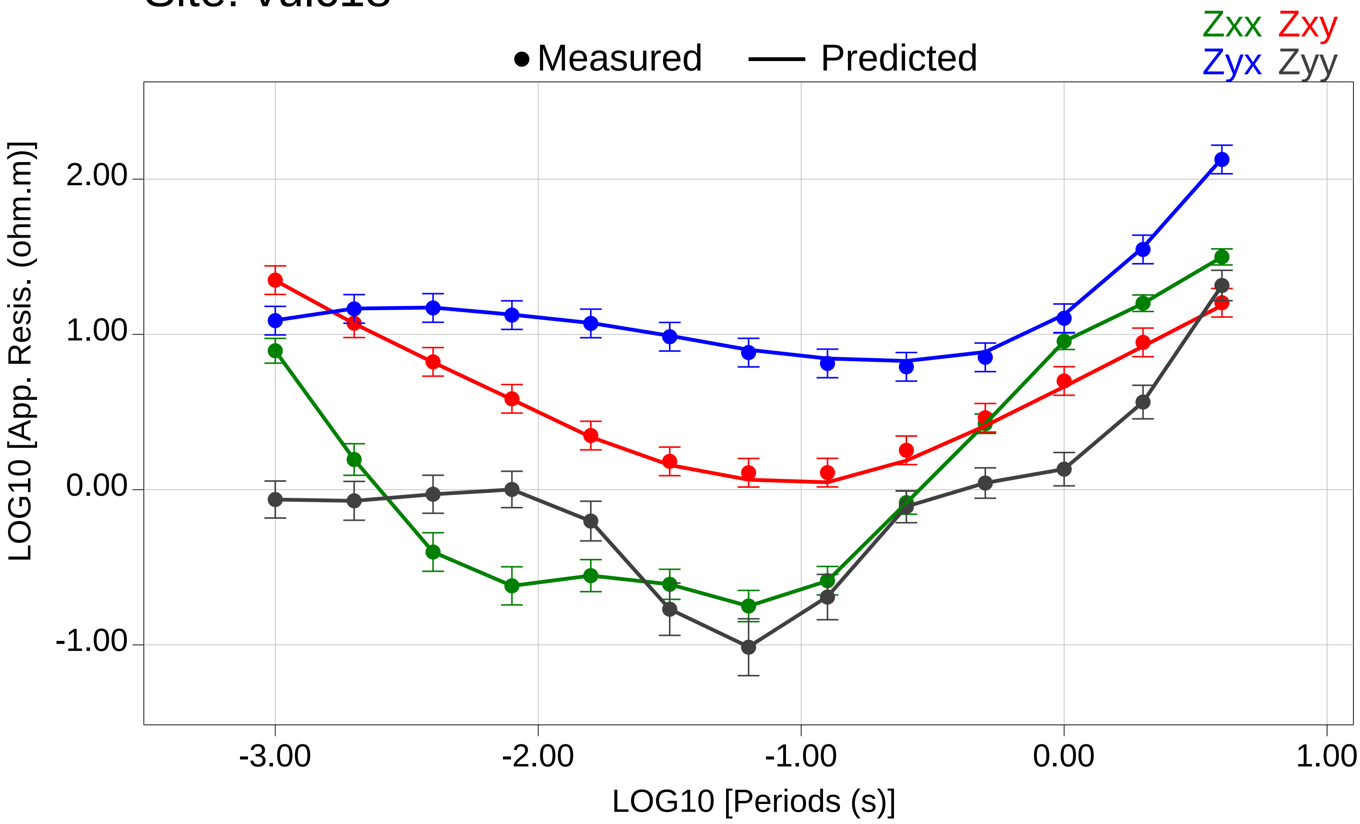

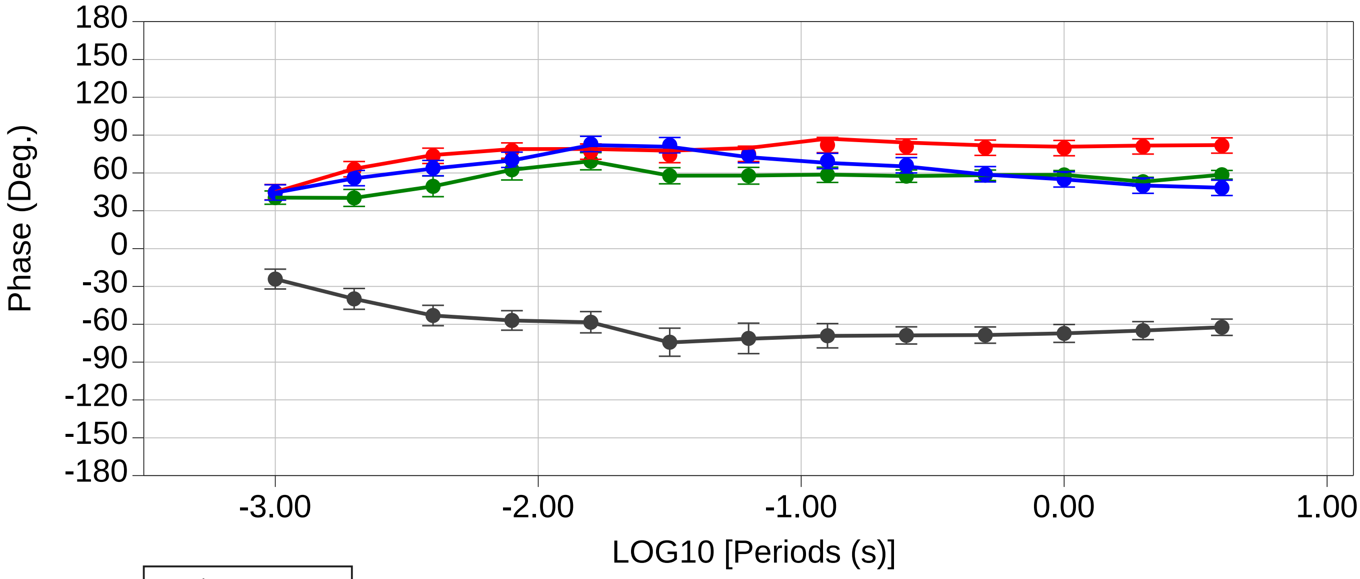

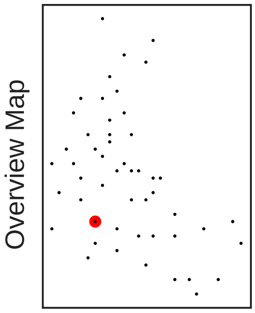

Overall RMS (Z+Tz)= 0.15
Total Z RMS = 0.15
Zxx RMS = 0.01
Zxy RMS = 0.27
Zyx RMS = 0.13
Zyy RMS = 0.01

Site: vulc19

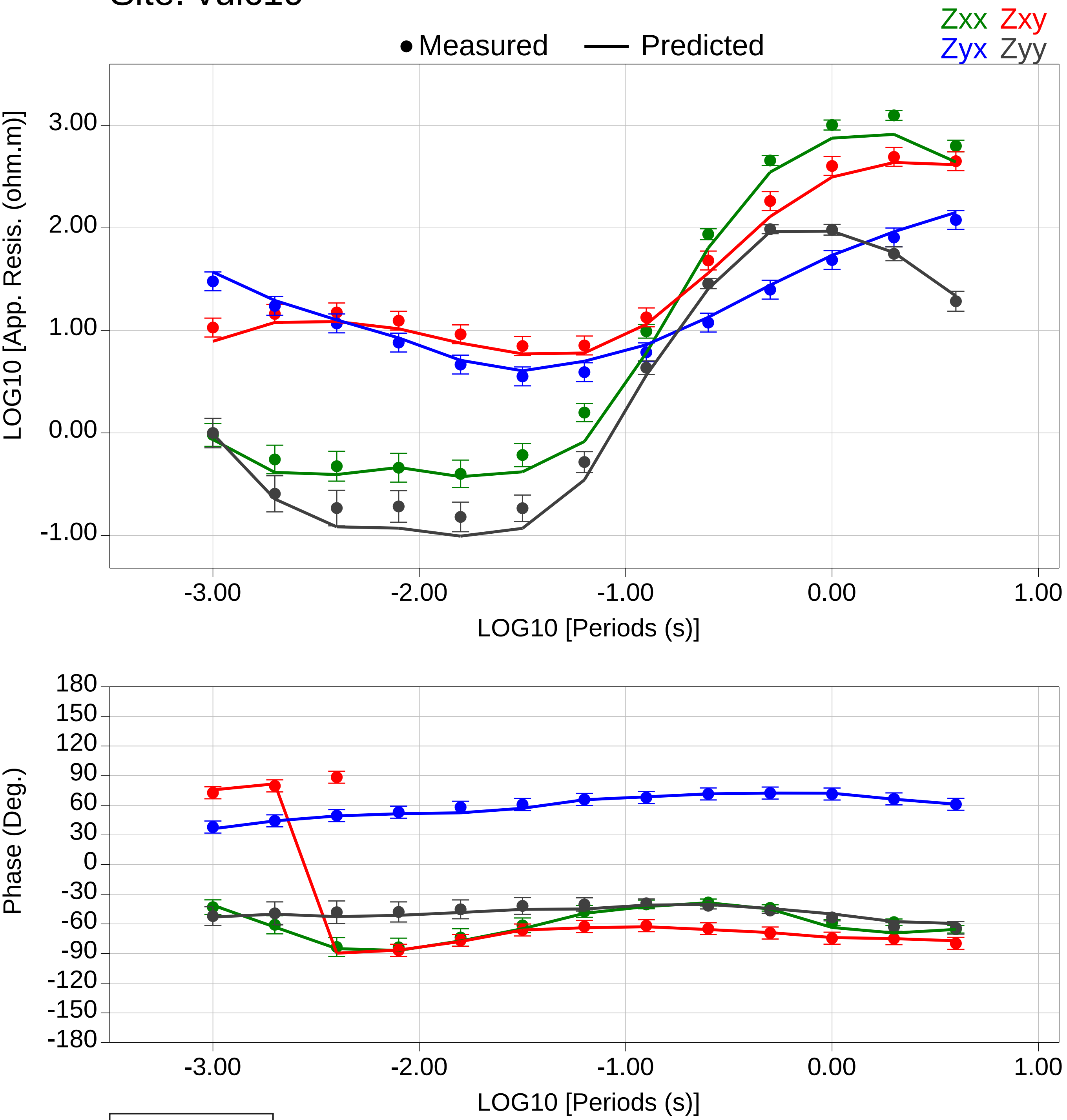

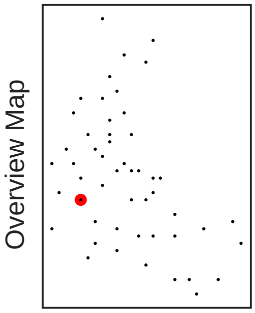

Overall RMS (Z+Tz)= 1.42
Total Z RMS = 1.42
Zxx RMS = 1.12
Zxy RMS = 2.53
Zyx RMS = 0.39
Zyy RMS = 0.55

Site: vulc20

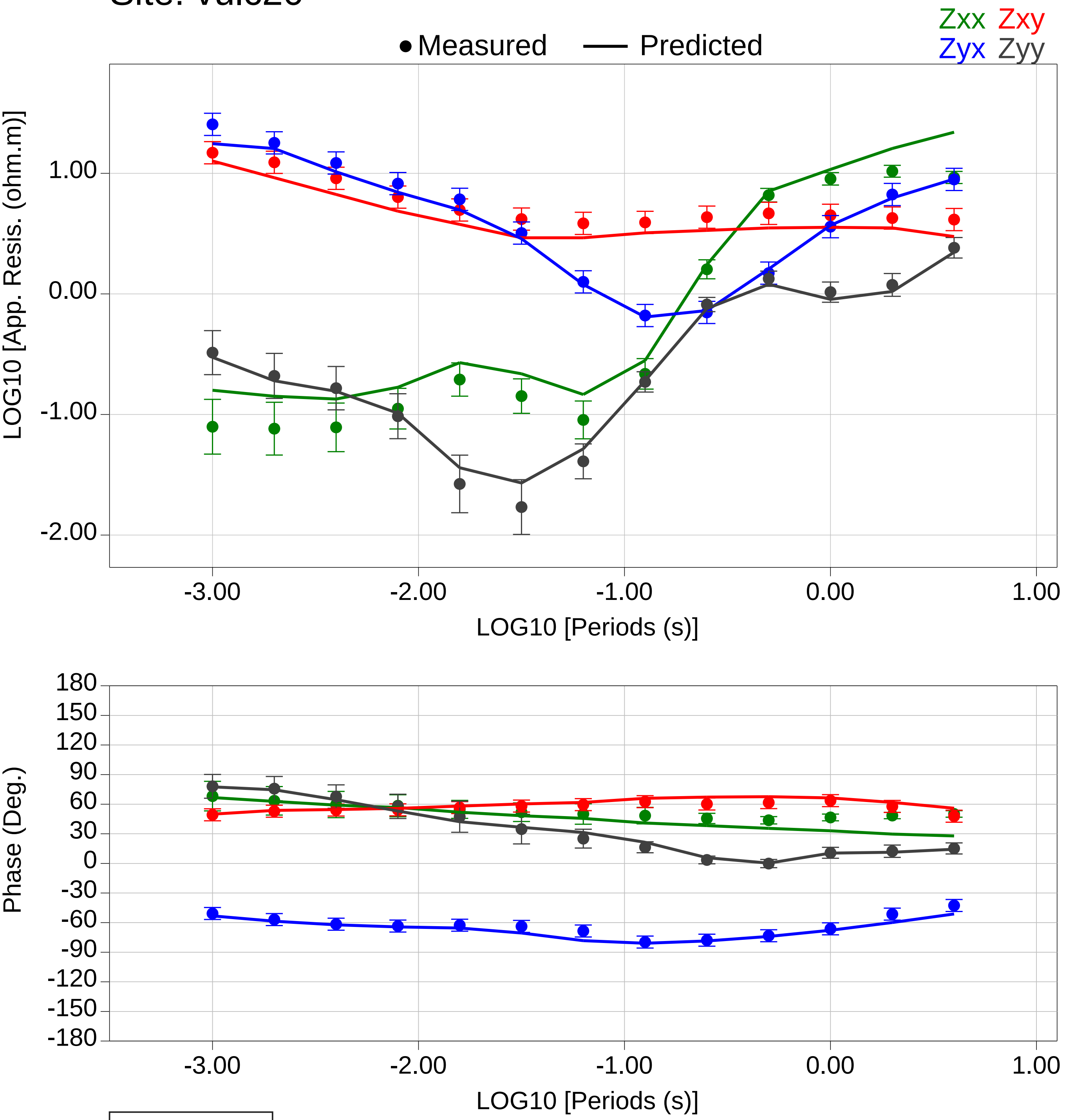

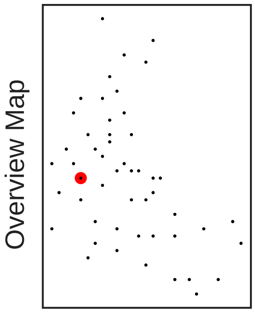

Overall RMS (Z+Tz)= 1.20
Total Z RMS = 1.20
Zxx RMS = 2.23
Zxy RMS = 0.66
Zyx RMS = 0.51
Zyy RMS = 0.34

Site: vulc21

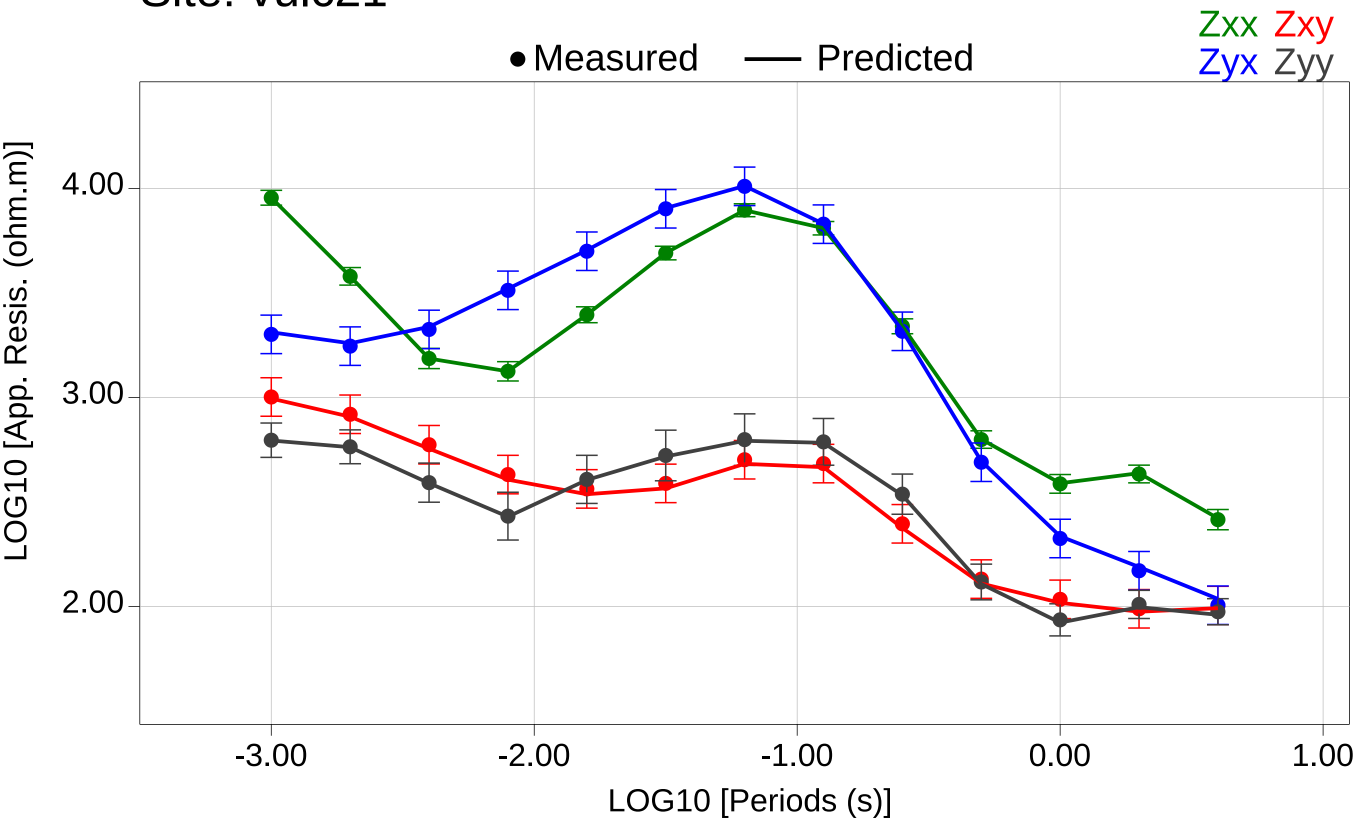

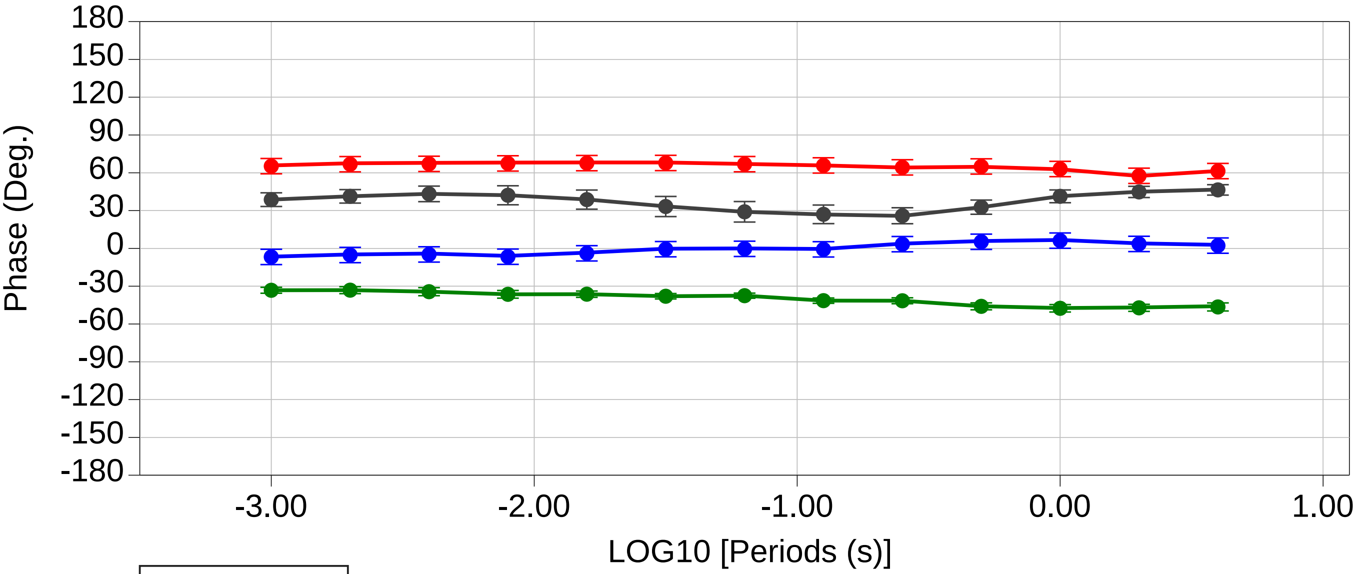

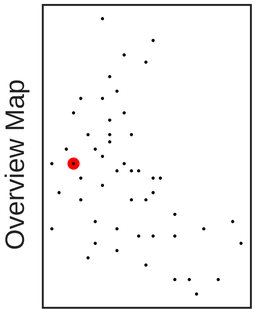

Overall RMS (Z+Tz)= 0.07
Total Z RMS = 0.07
Zxx RMS = 0.04
Zxy RMS = 0.11
Zyx RMS = 0.08
Zyy RMS = 0.05

Site: vulc22

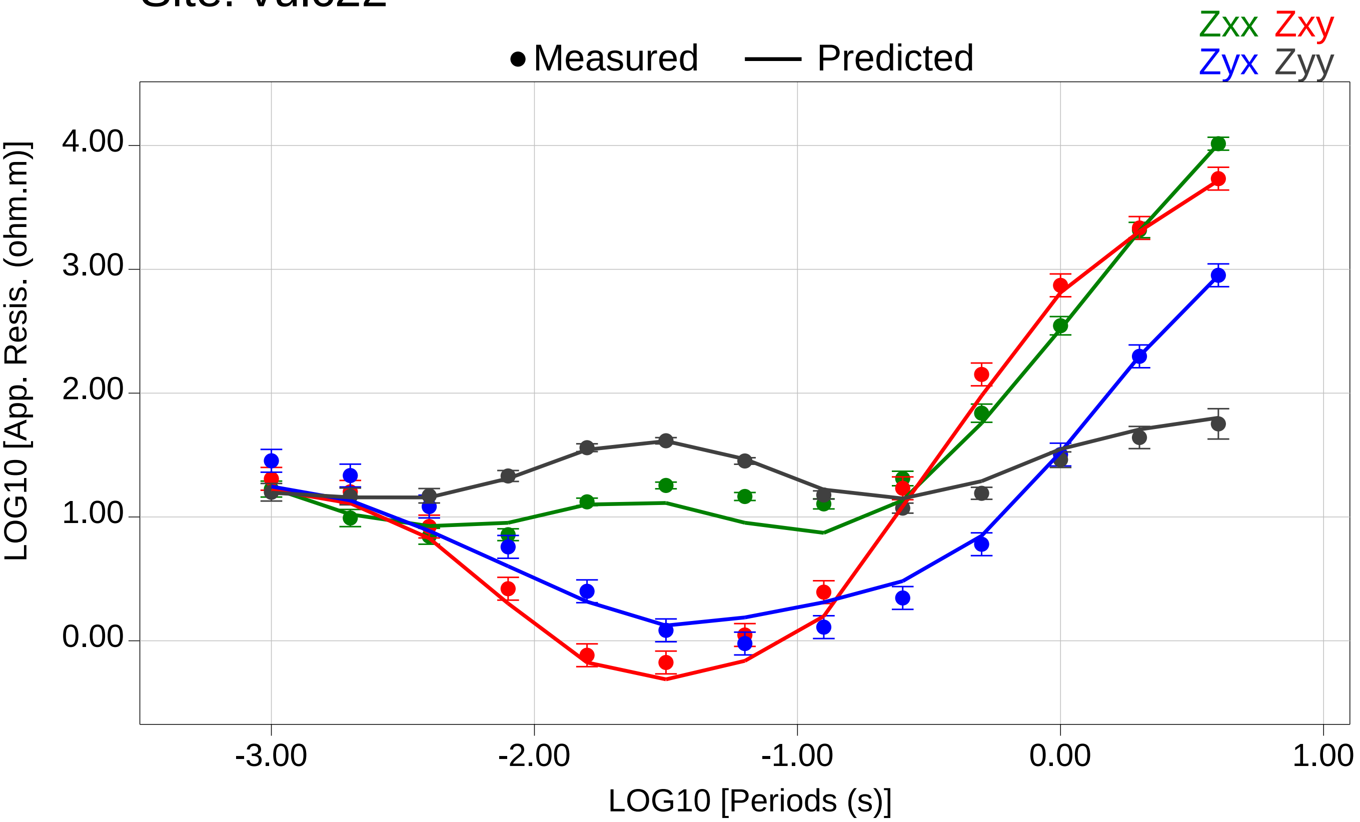

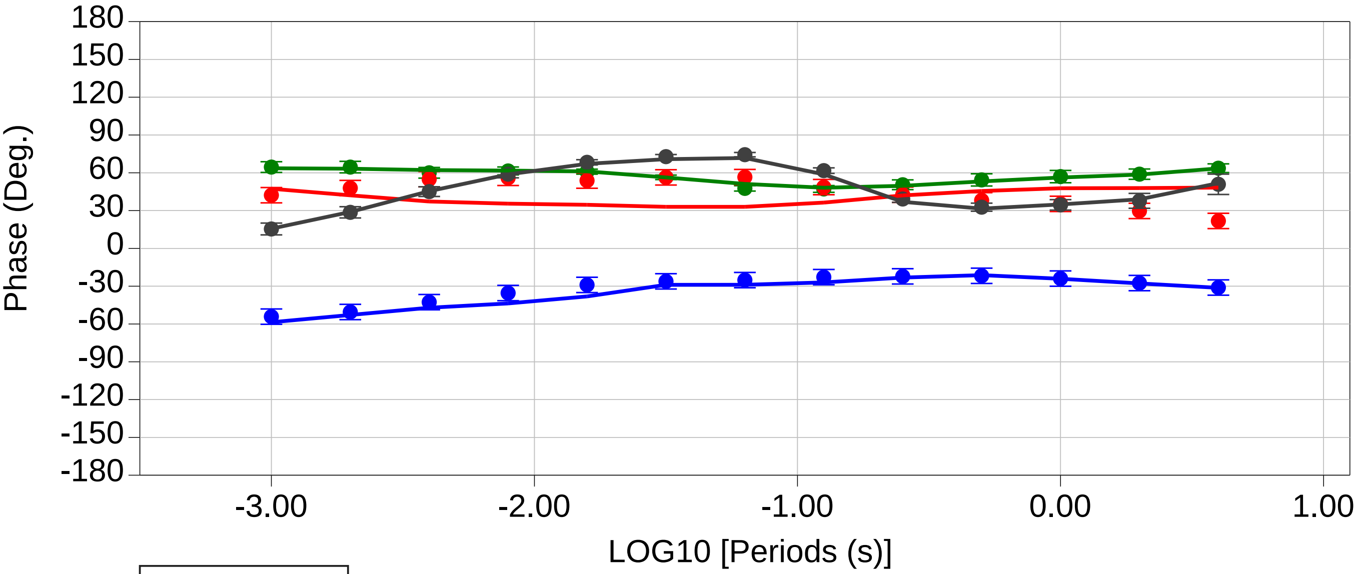

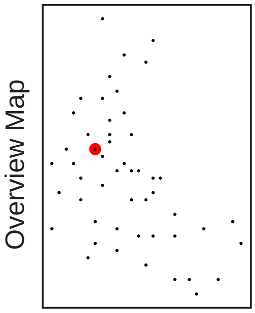

Overall RMS (Z+Tz)= 1.13
Total Z RMS = 1.13
Zxx RMS = 1.40
Zxy RMS = 1.43
Zyx RMS = 0.84
Zyy RMS = 0.64

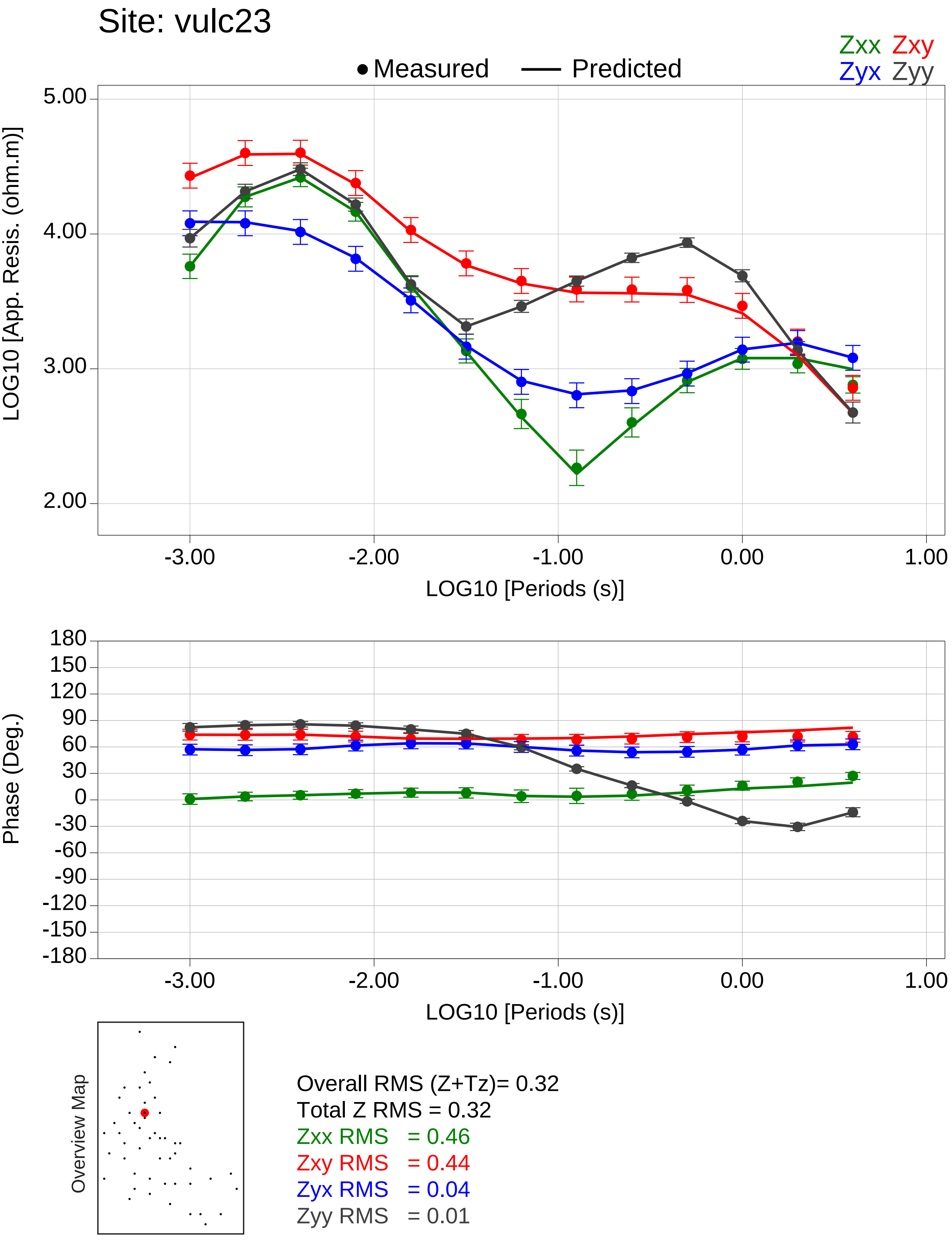

Overall RMS (Z+Tz)= 0.32
Total Z RMS = 0.32
Zxx RMS = 0.46
Zxy RMS = 0.44
Zyx RMS = 0.04
Zyy RMS = 0.01

Site: vulc24

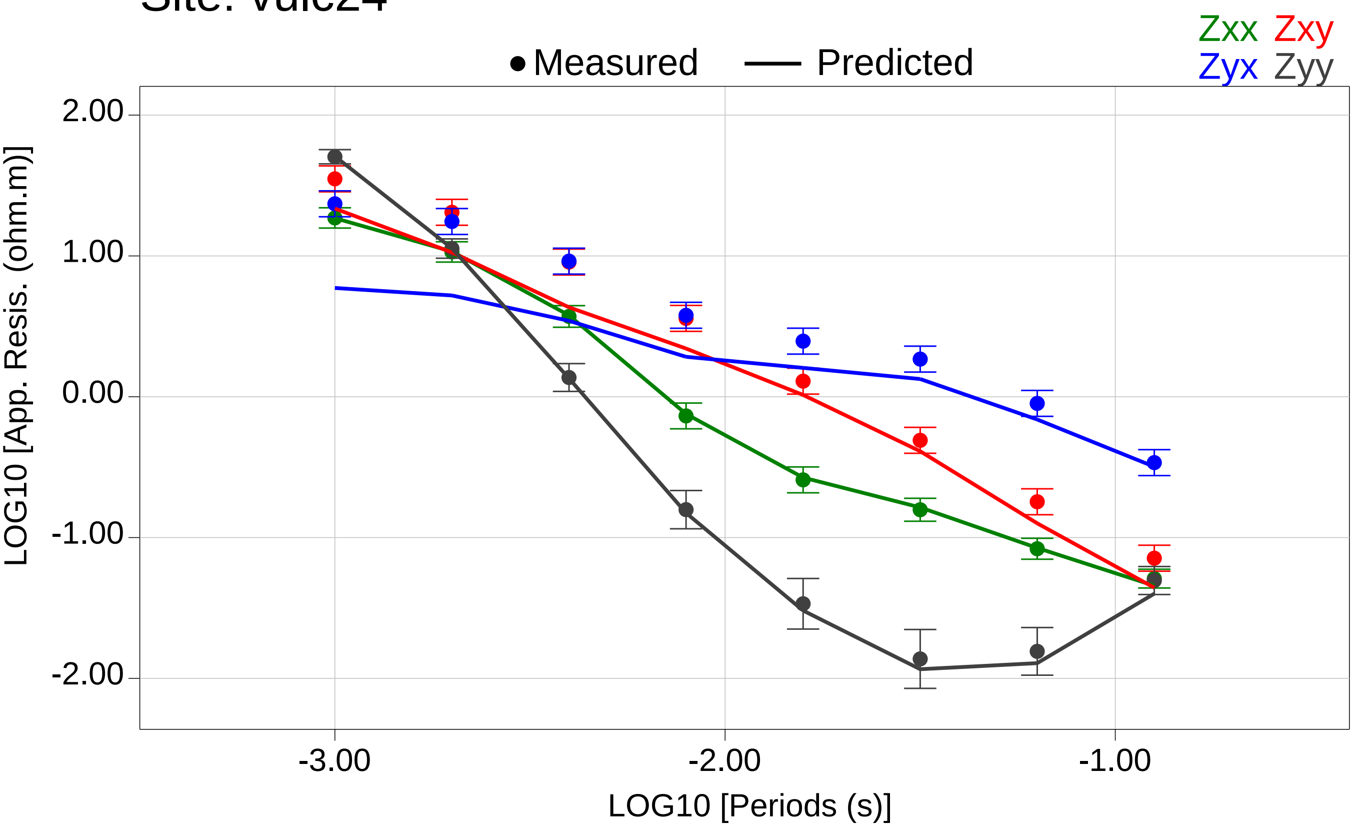

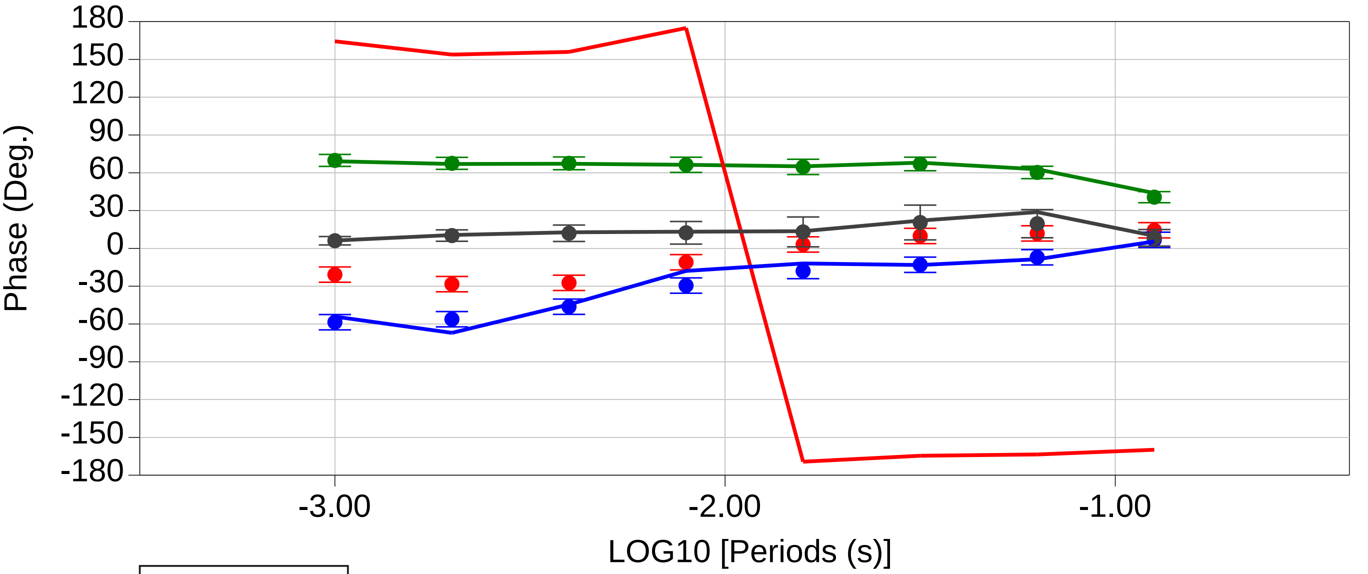

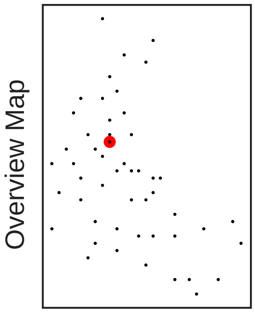

Overall RMS (Z+Tz)= 4.31
Total Z RMS = 4.31
Zxx RMS = 0.22
Zxy RMS = 8.49
Zyx RMS = 1.51
Zyy RMS = 0.25

Site: vulc25

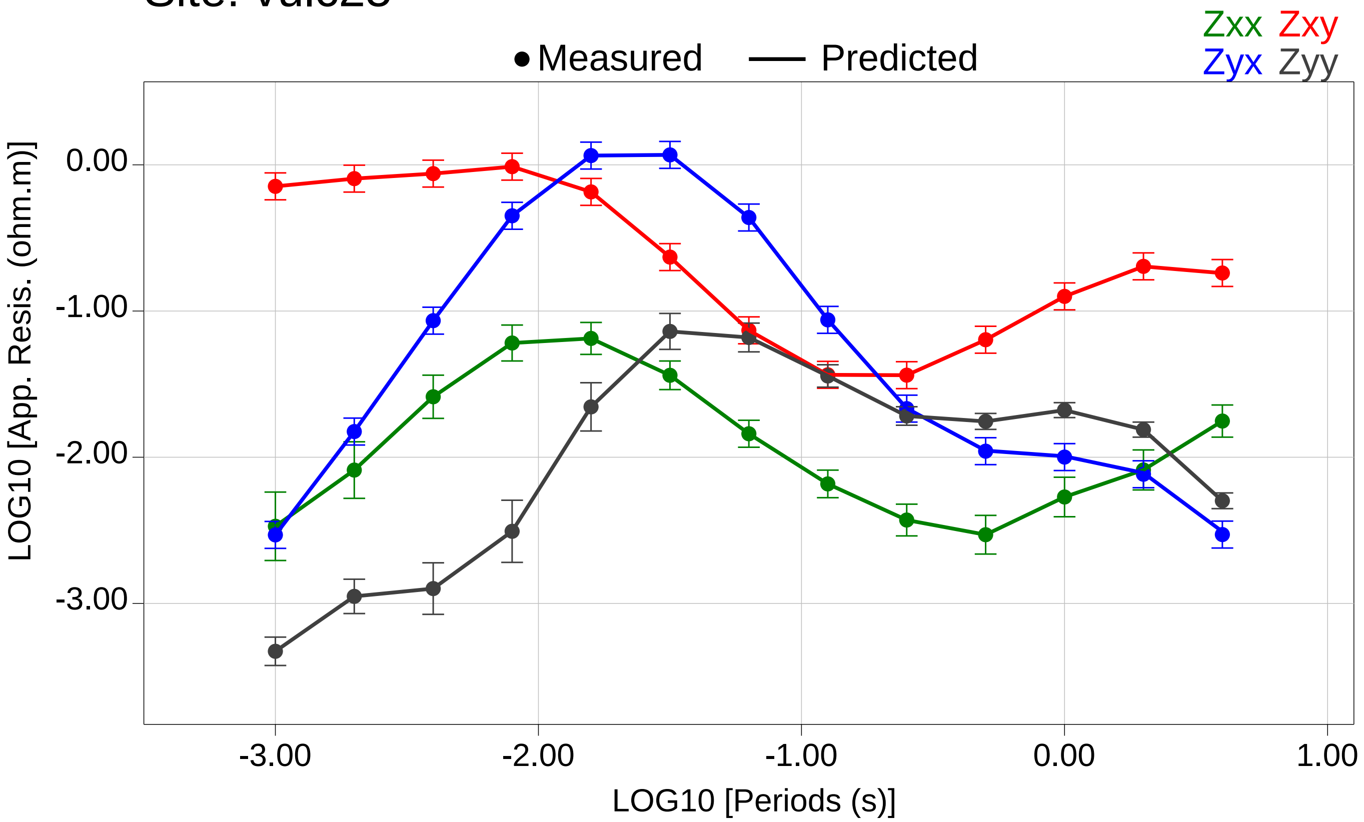

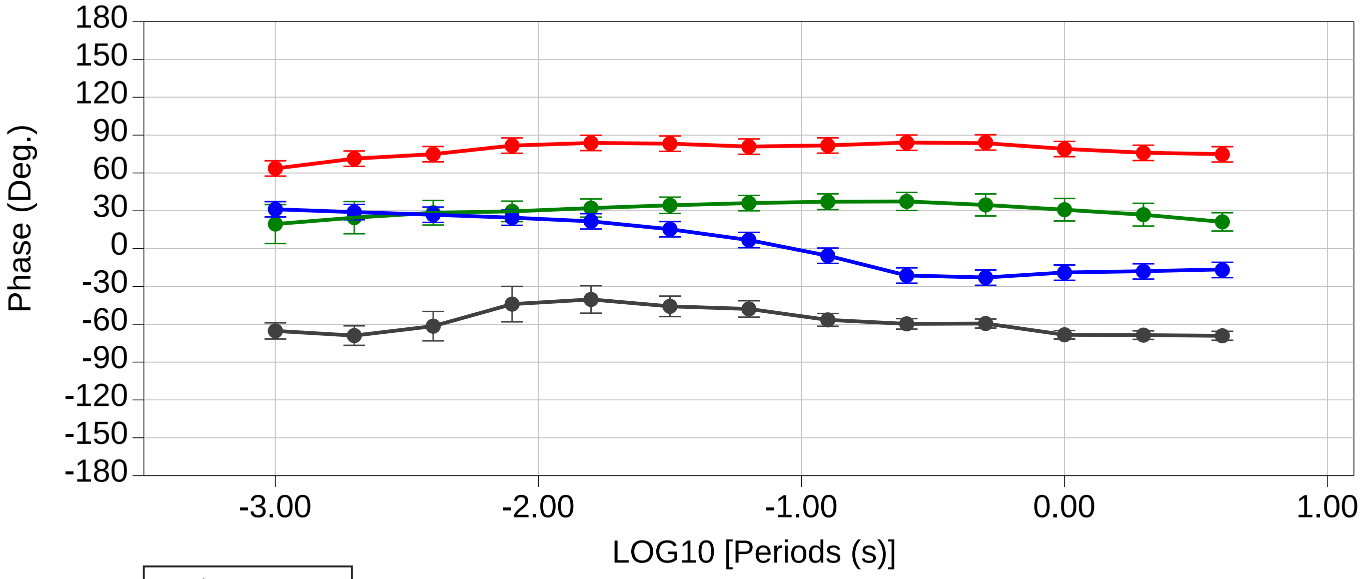

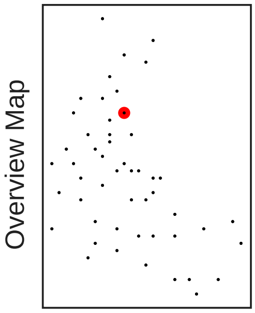

Overall RMS (Z+Tz)= 0.02
Total Z RMS = 0.02
Zxx RMS = 0.00
Zxy RMS = 0.02
Zyx RMS = 0.04
Zyy RMS = 0.00

Site: vulc26

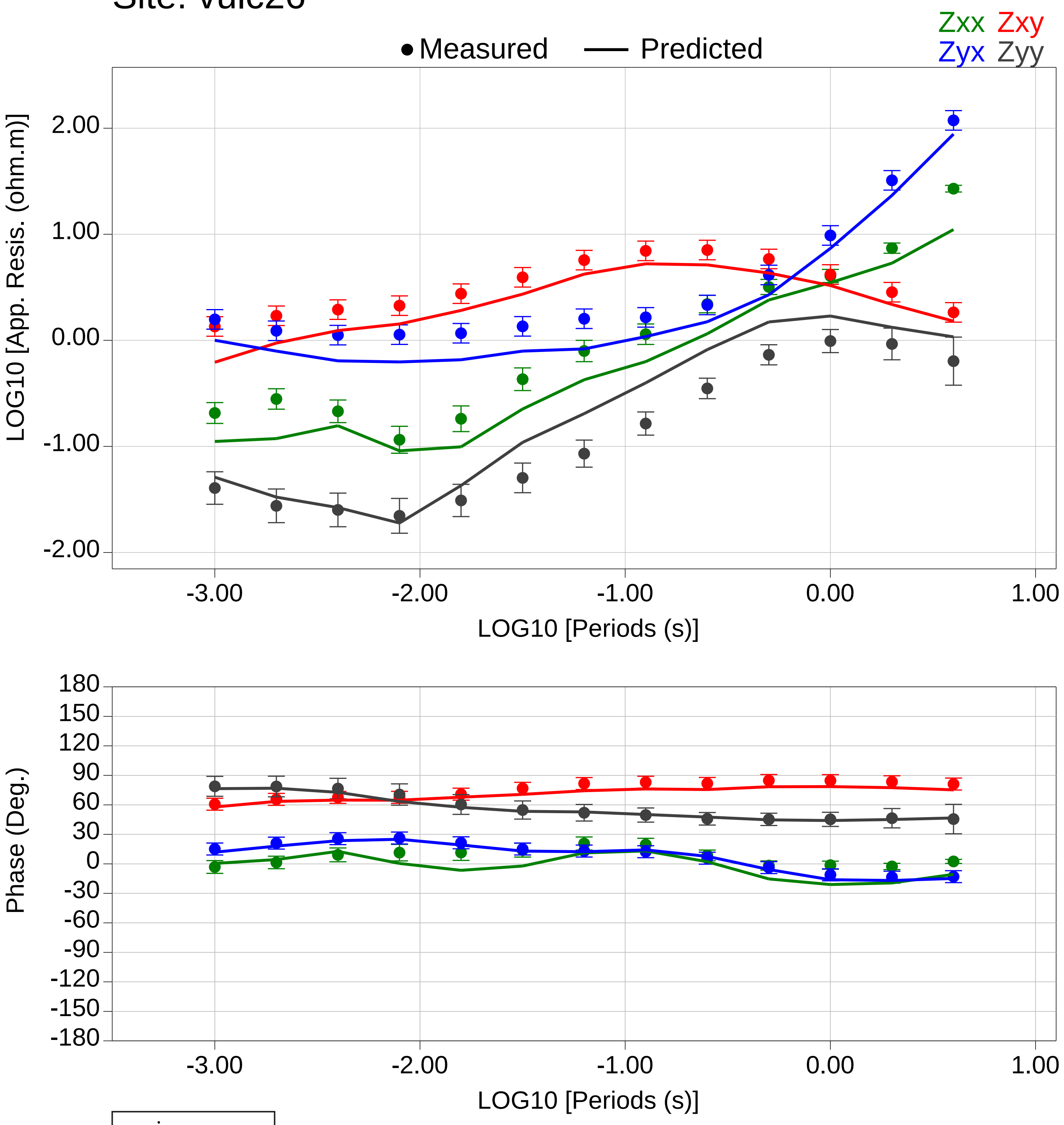

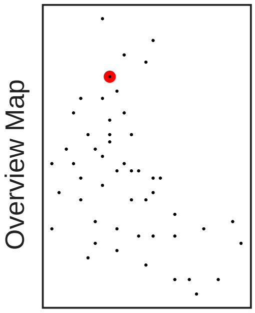

Overall RMS (Z+Tz)= 1.44
Total Z RMS = 1.44
Zxx RMS = 2.17
Zxy RMS = 0.93
Zyx RMS = 1.00
Zyy RMS = 1.32

Site: vulc27

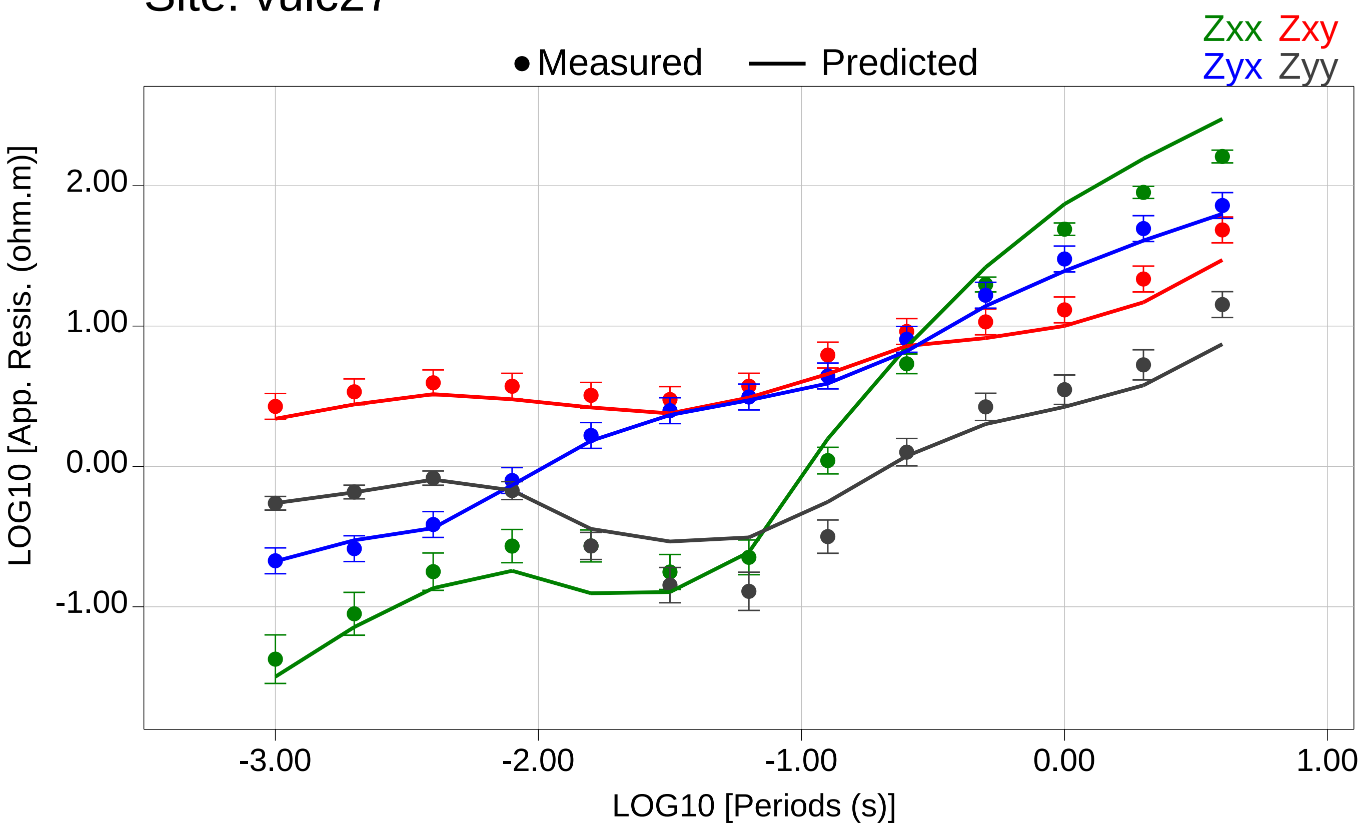

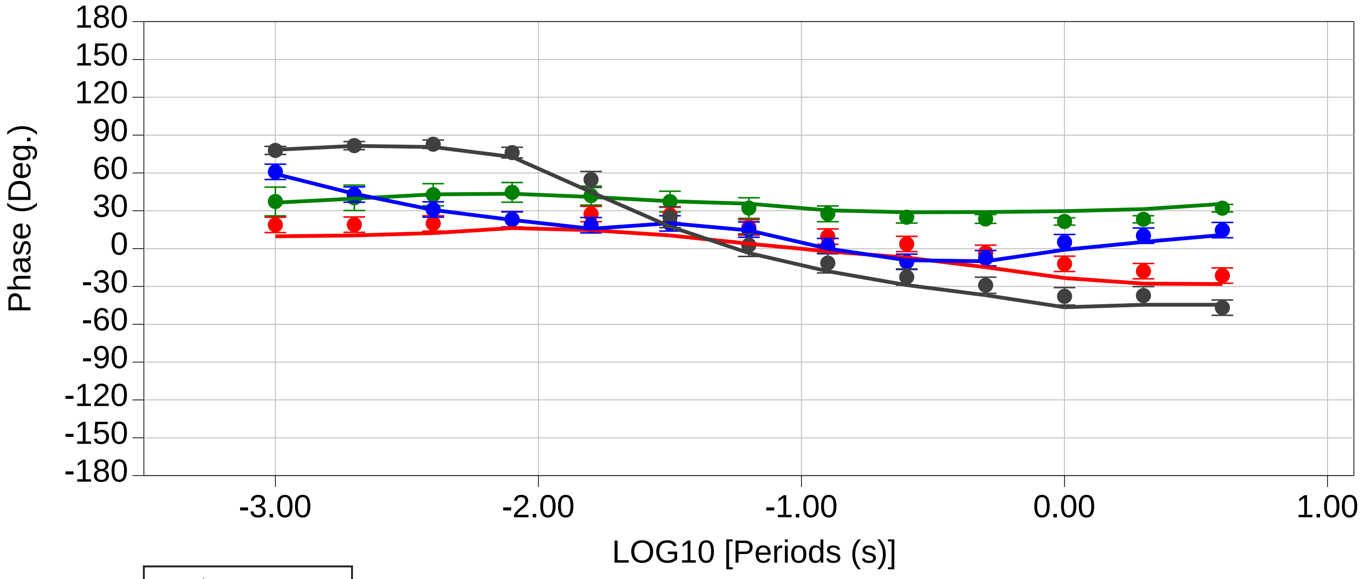

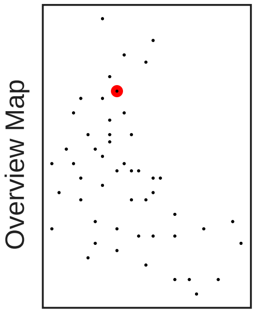

Overall RMS (Z+Tz)= 1.14
Total Z RMS = 1.14
Zxx RMS = 1.75
Zxy RMS = 1.02
Zyx RMS = 0.37
Zyy RMS = 0.99

Site: vulc28

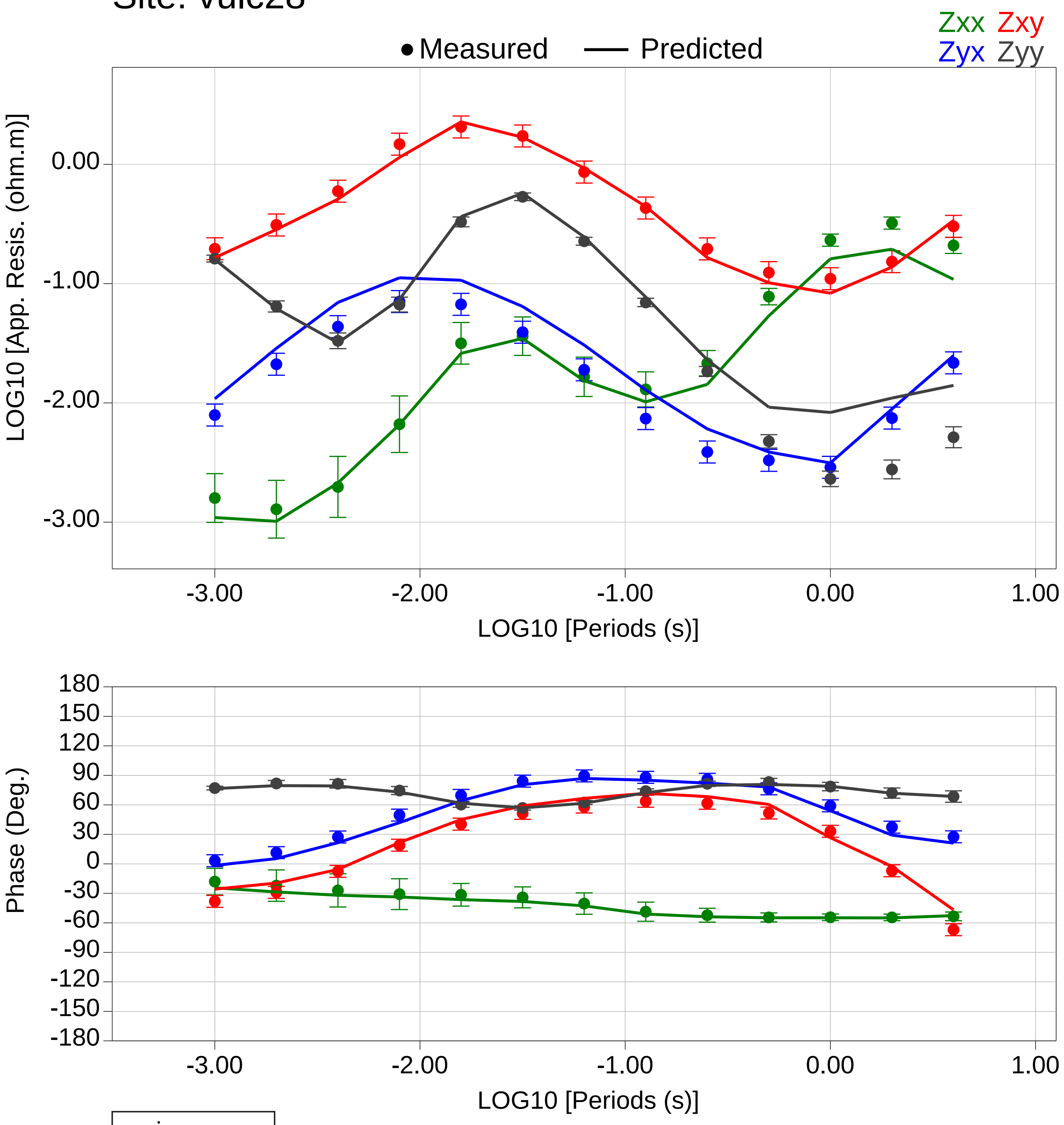

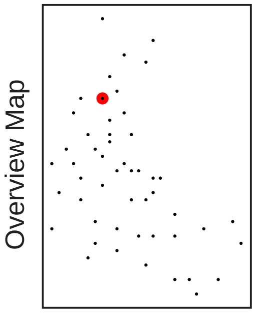

Overall RMS (Z+Tz)= 1.55
Total Z RMS = 1.55
Zxx RMS = 0.92
Zxy RMS = 0.82
Zyx RMS = 1.12
Zyy RMS = 2.62

Site: vulc29

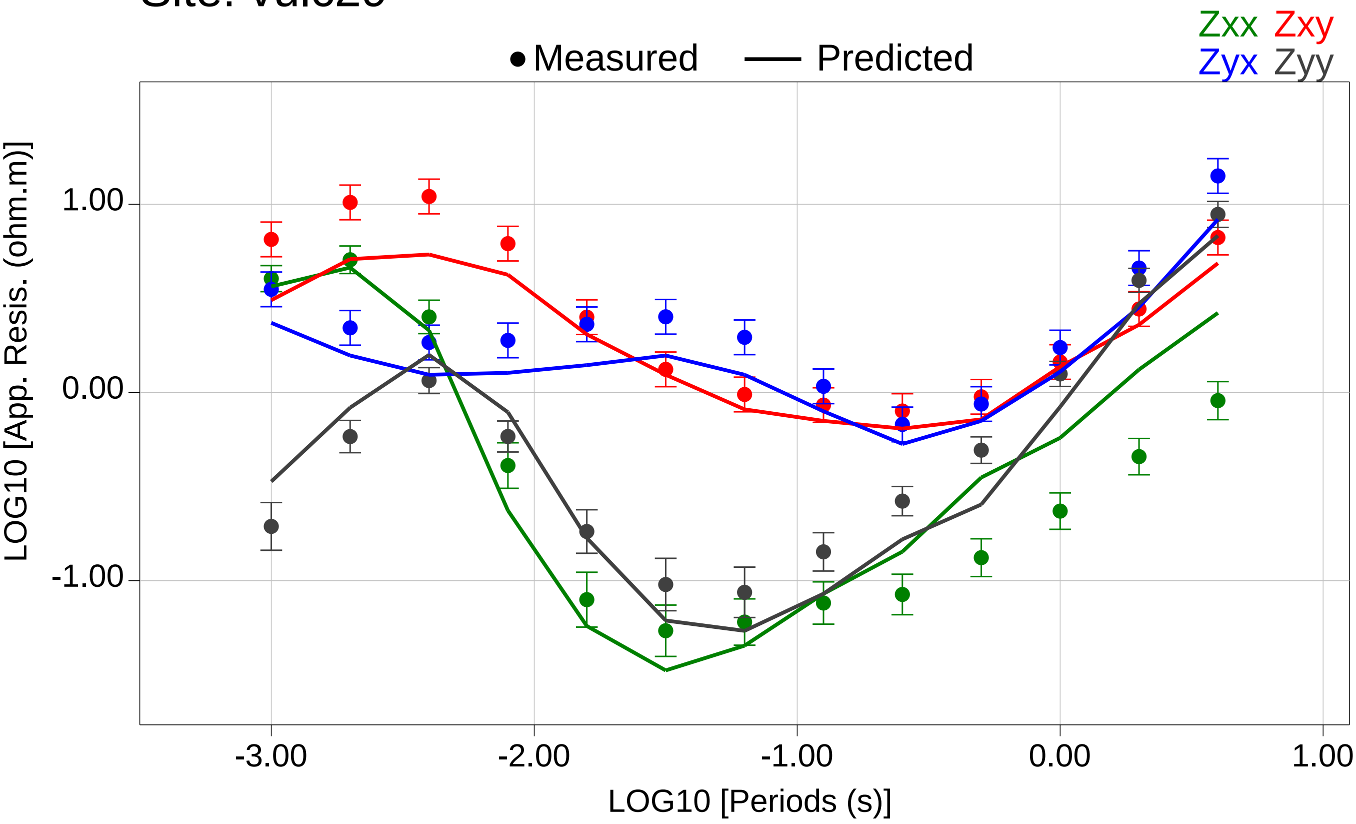

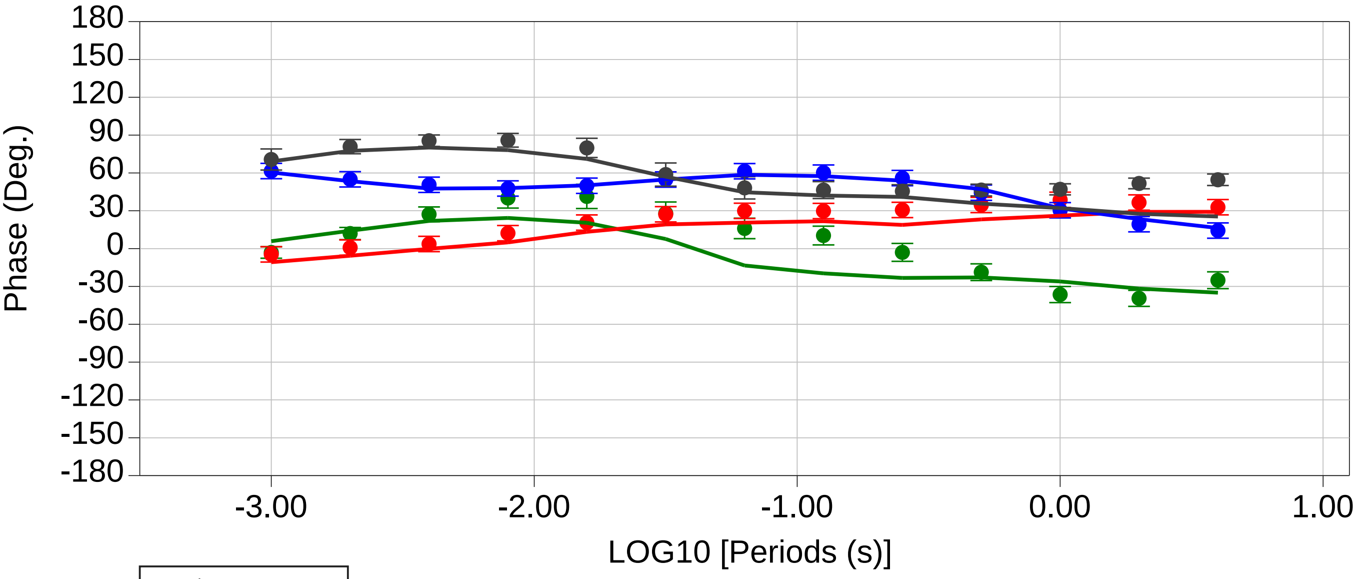

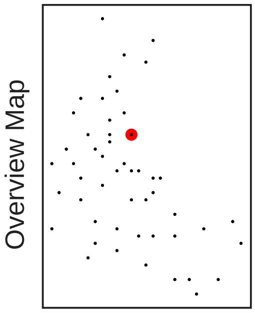

Overall RMS (Z+Tz)= 1.46
Total Z RMS = 1.46
Zxx RMS = 2.02
Zxy RMS = 1.04
Zyx RMS = 0.86
Zyy RMS = 1.61

Site: vulc30

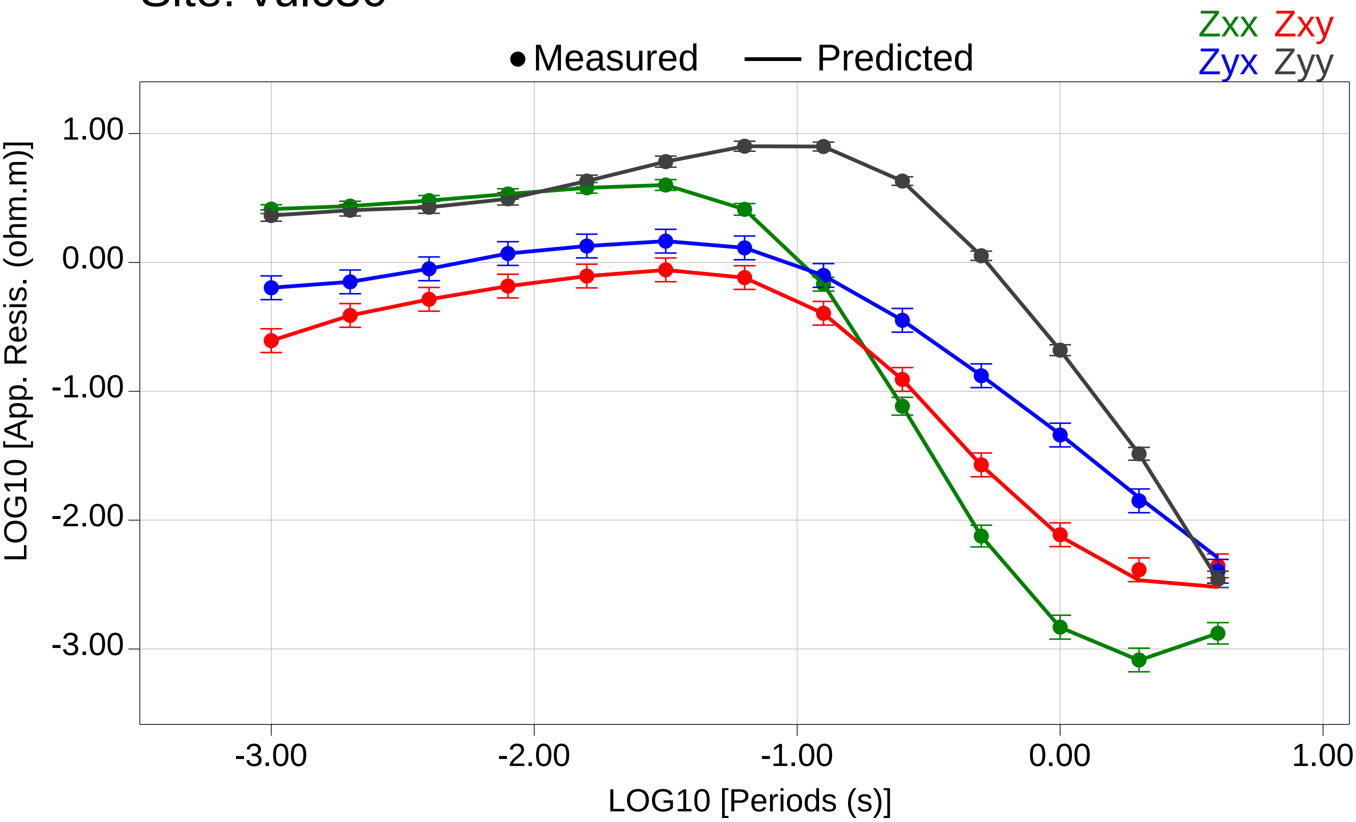

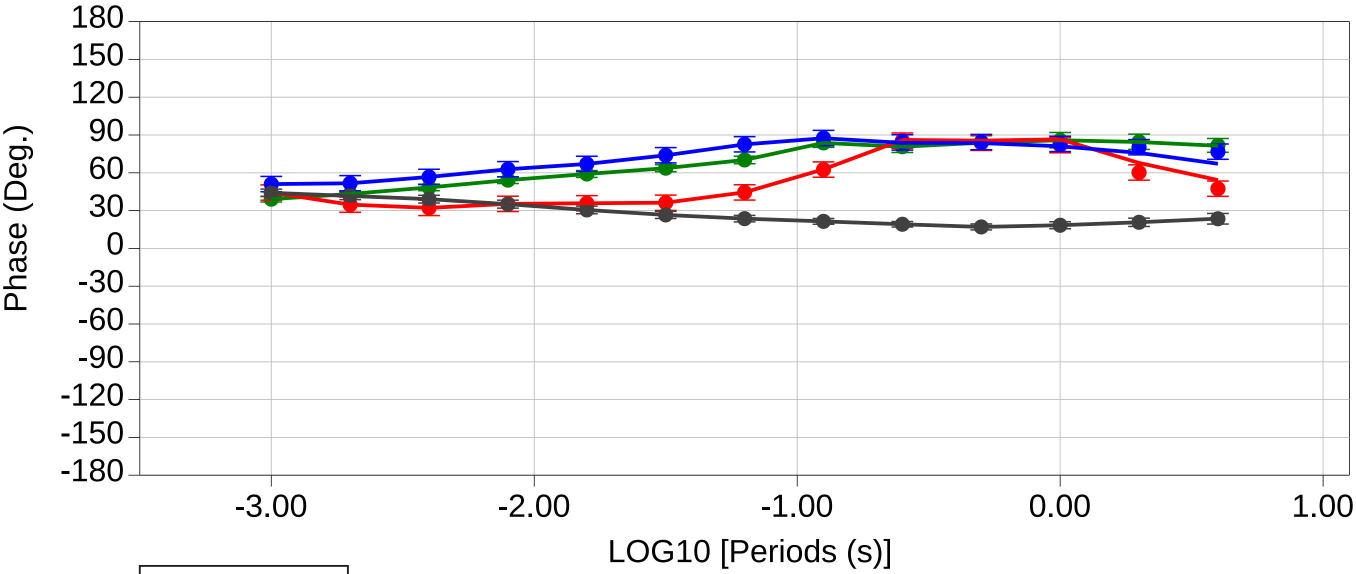

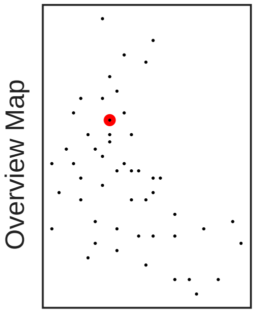

Overall RMS (Z+Tz)= 0.24
Total Z RMS = 0.24
Zxx RMS = 0.01
Zxy RMS = 0.35
Zyx RMS = 0.31
Zyy RMS = 0.00

Site: vulc31

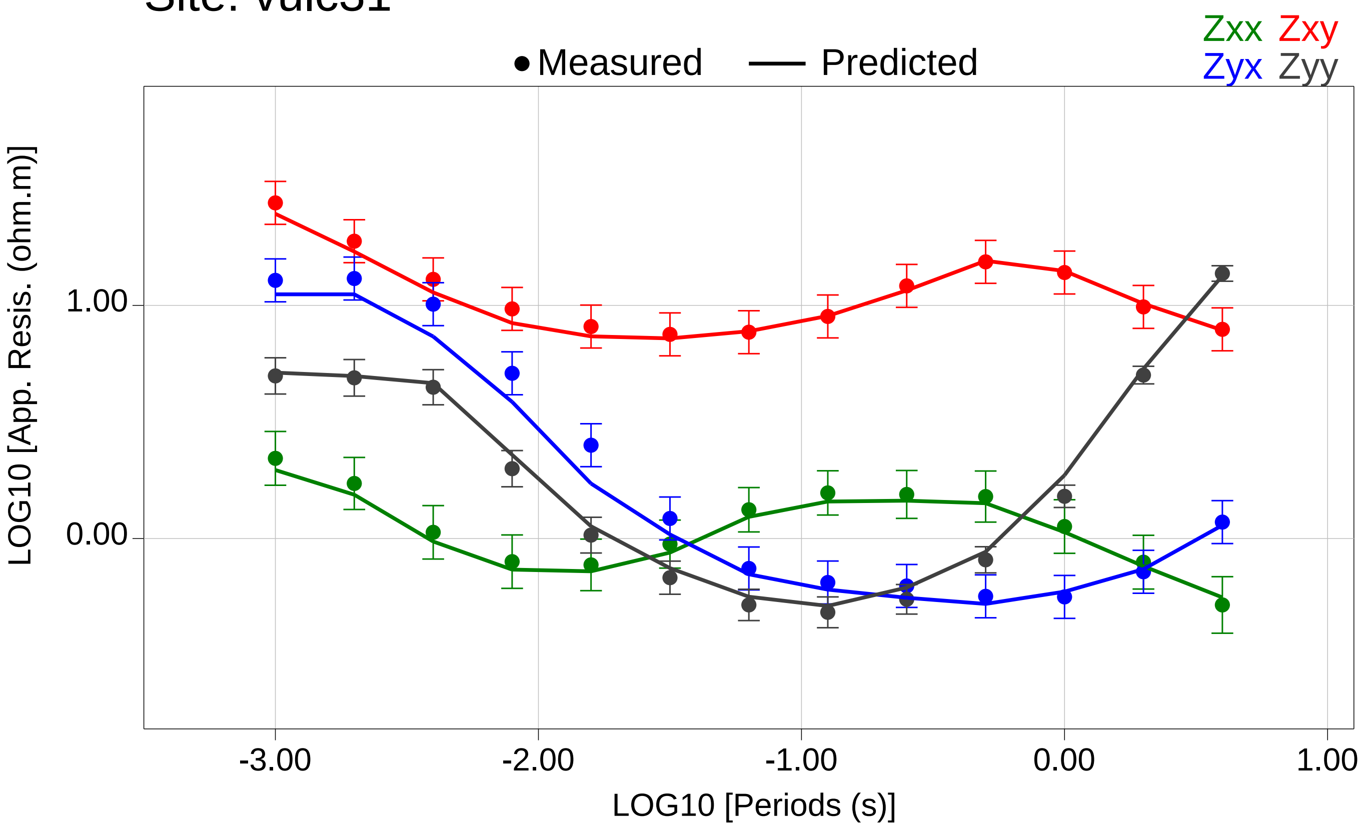

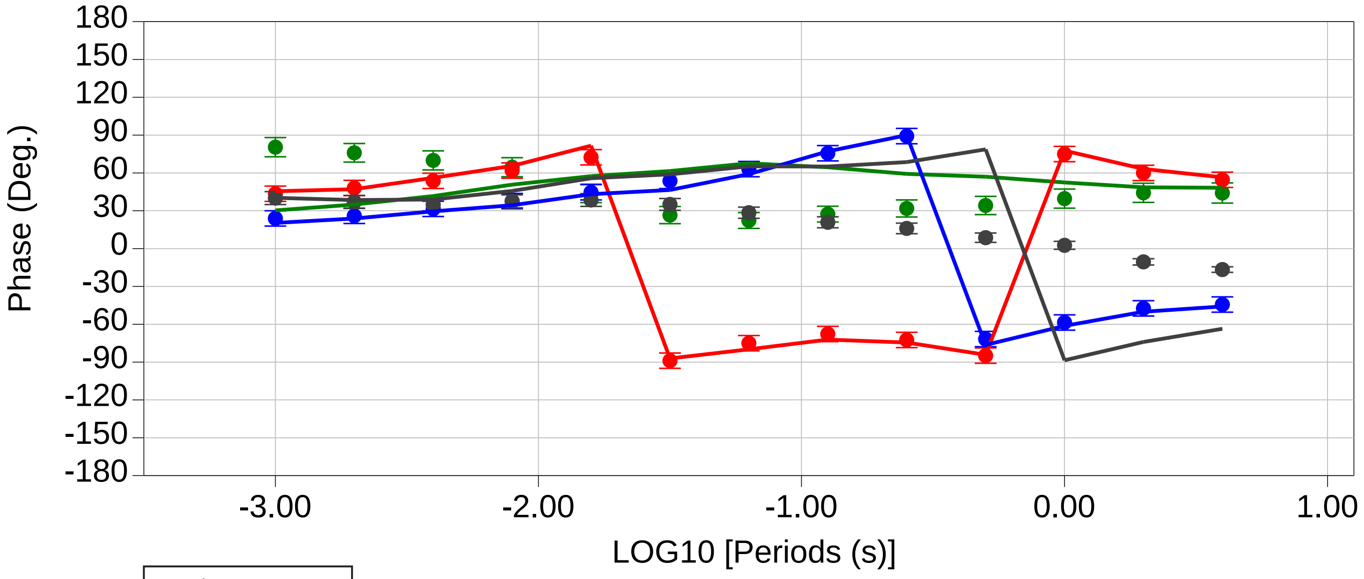

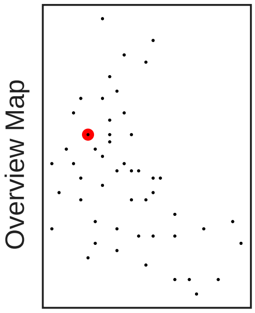

Overall RMS (Z+Tz)= 3.62
Total Z RMS = 3.62
Zxx RMS = 2.06
Zxy RMS = 0.35
Zyx RMS = 0.48
Zyy RMS = 6.91

Site: vulc32

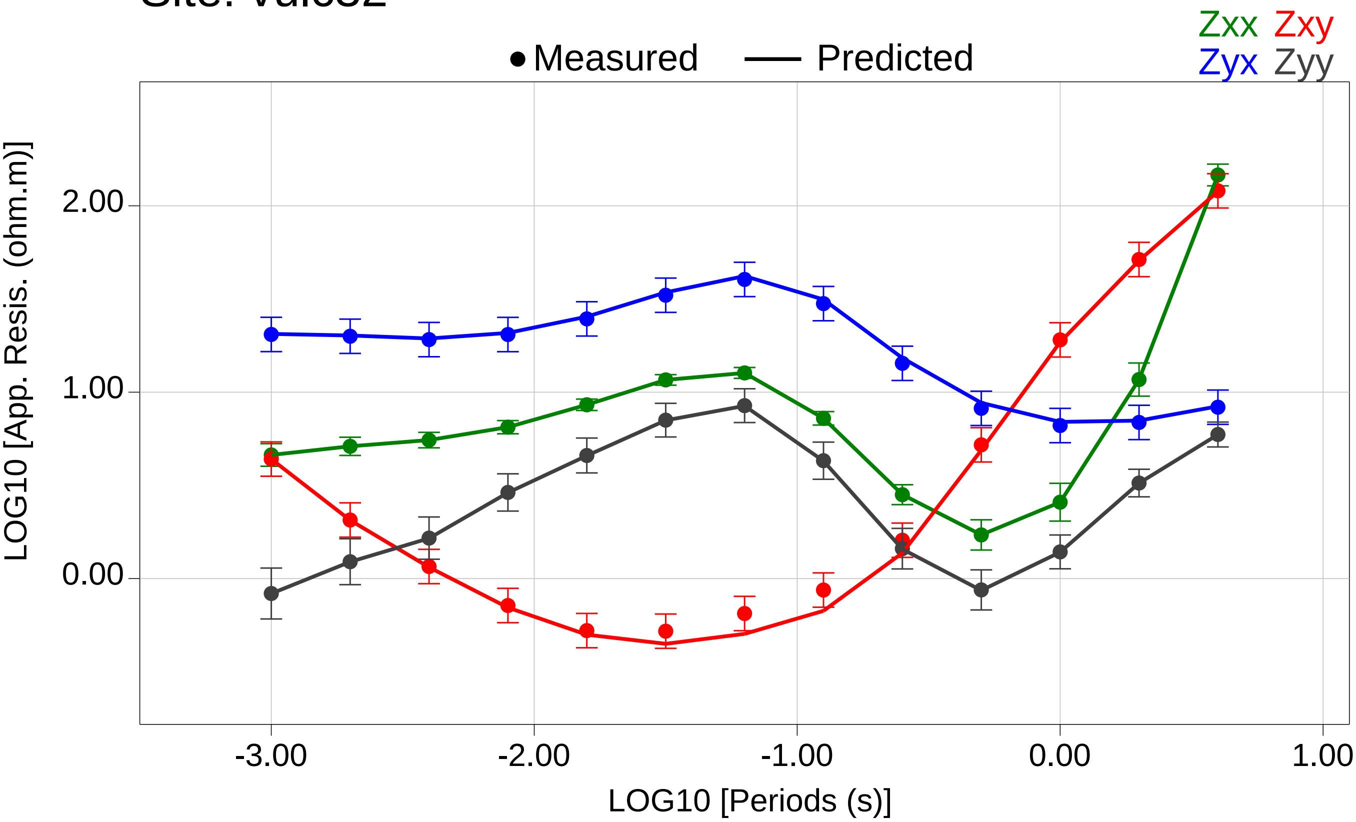

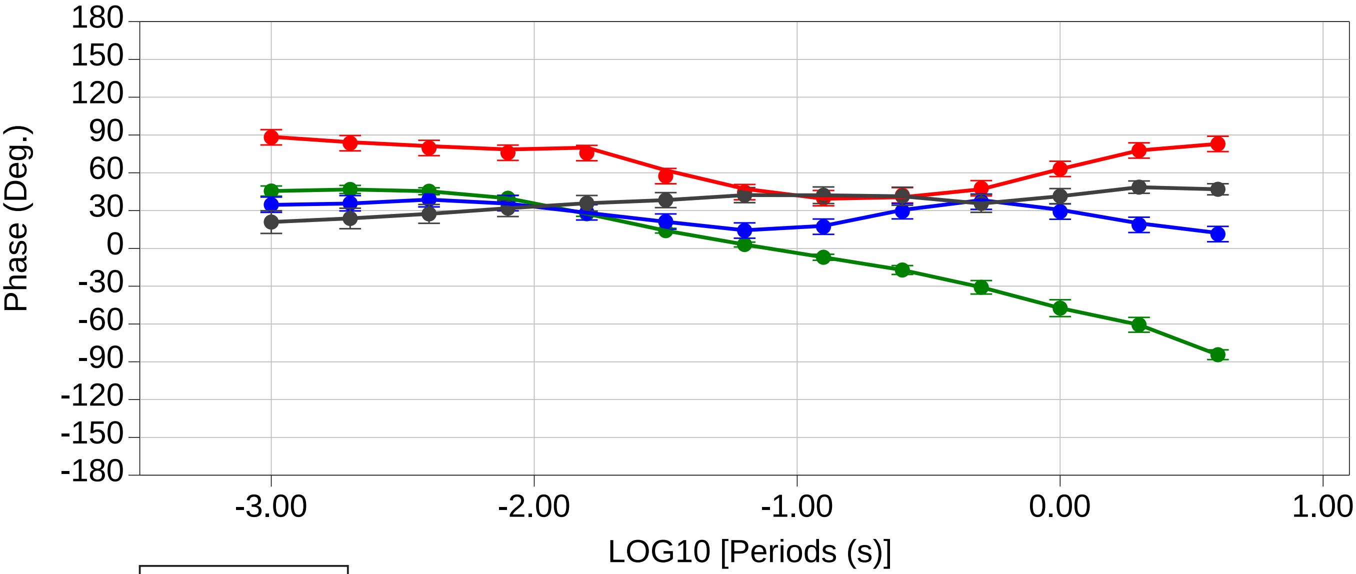

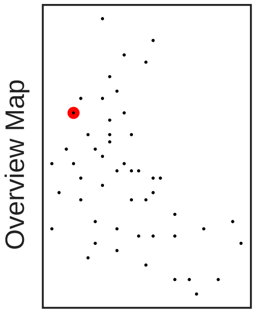

Overall RMS (Z+Tz)= 0.17
Total Z RMS = 0.17
Zxx RMS = 0.00
Zxy RMS = 0.32
Zyx RMS = 0.11
Zyy RMS = 0.01

Site: vulc33

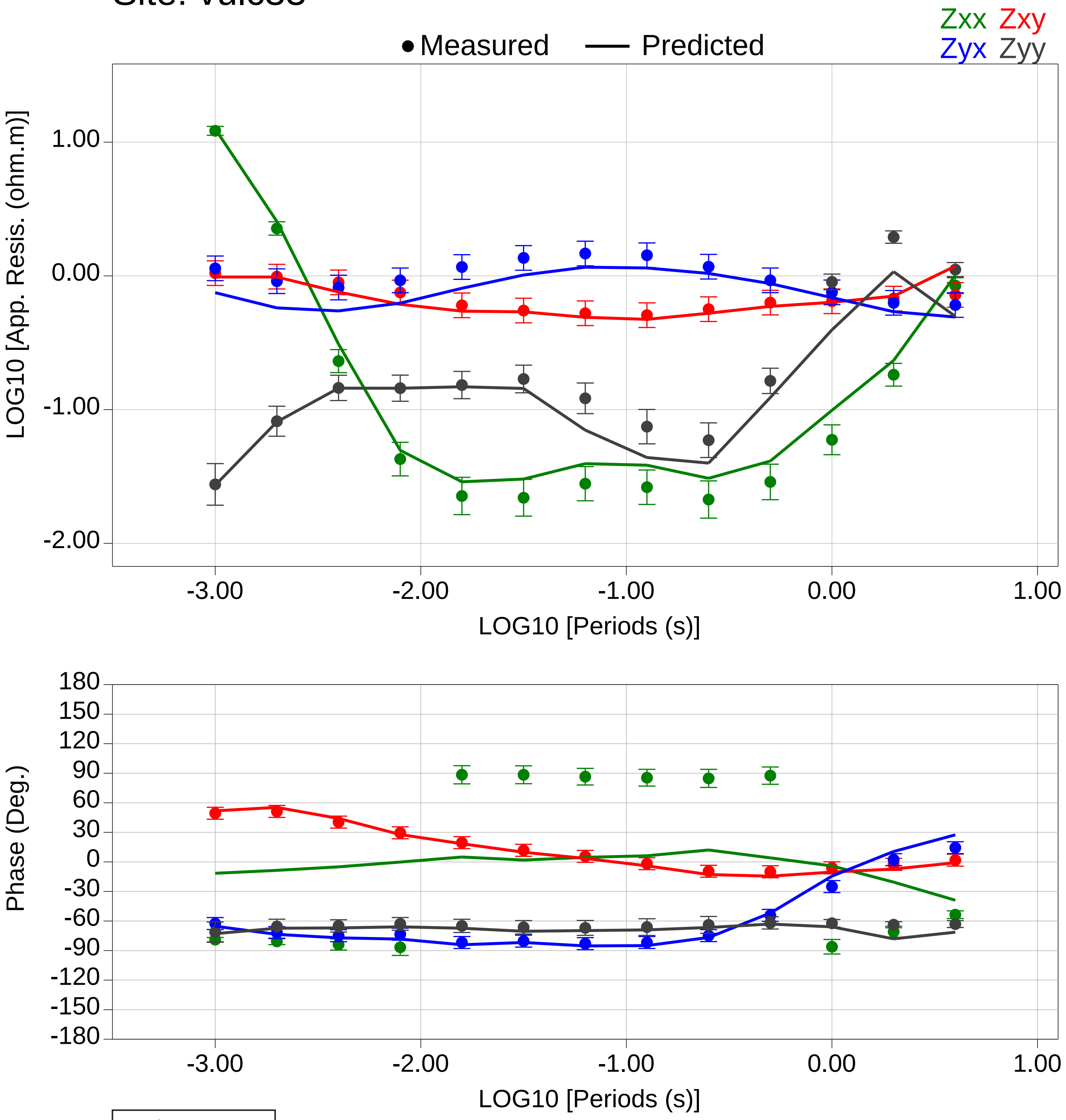

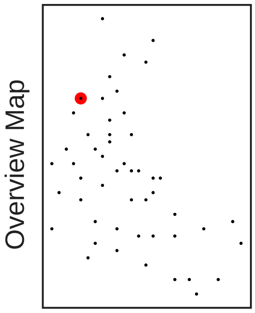

Overall RMS (Z+Tz)= 3.47
Total Z RMS = 3.47
Zxx RMS = 6.72
Zxy RMS = 0.50
Zyx RMS = 0.77
Zyy RMS = 1.45

Site: vulc34

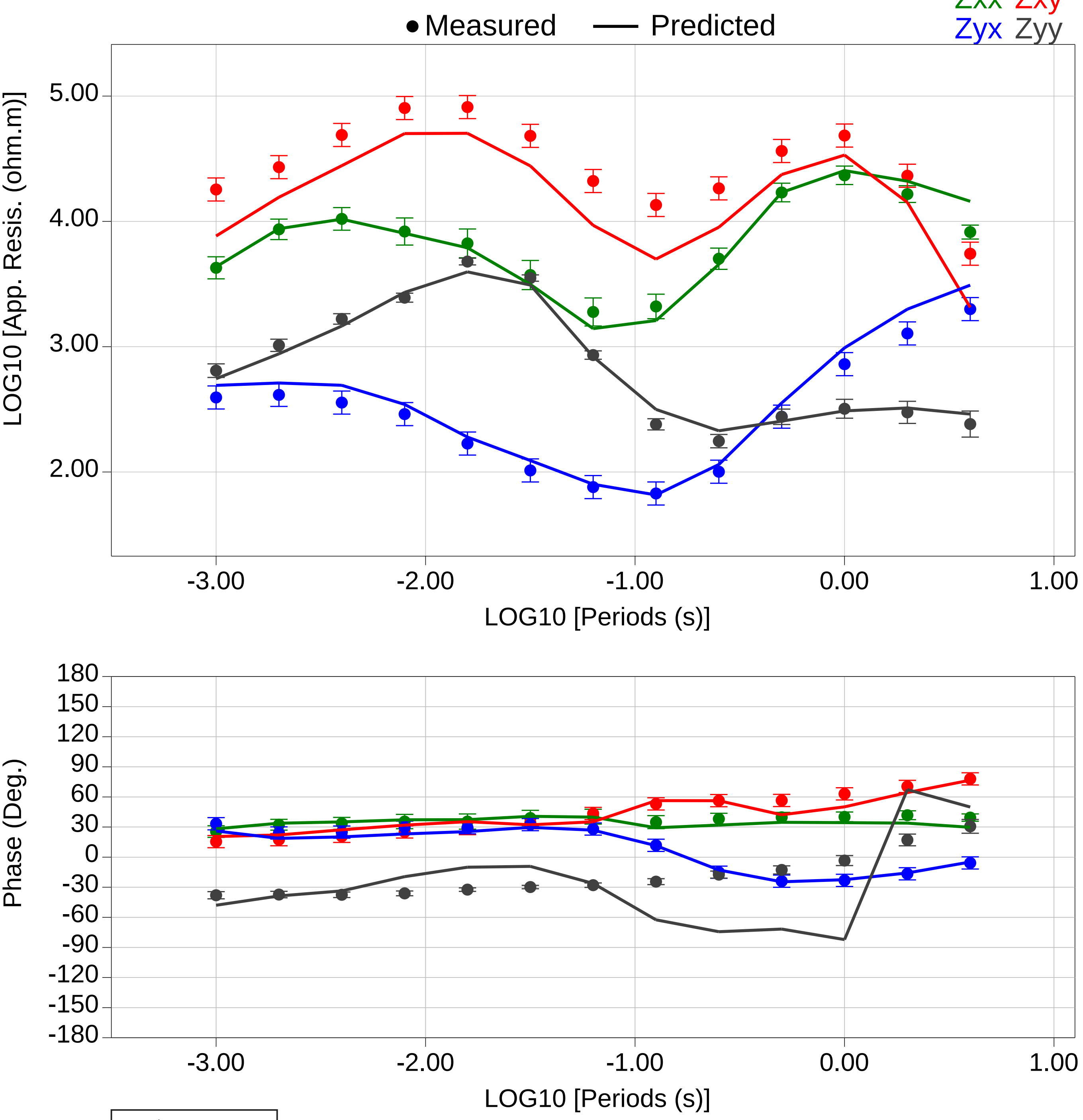

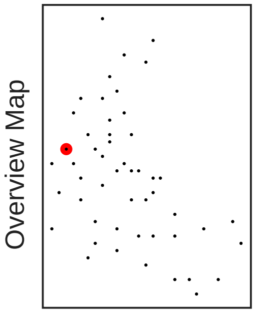

Overall RMS (Z+Tz)= 2.67
Total Z RMS = 2.67
Zxx RMS = 0.98
Zxy RMS = 1.41
Zyx RMS = 0.73
Zyy RMS = 5.01

Site: vulc35

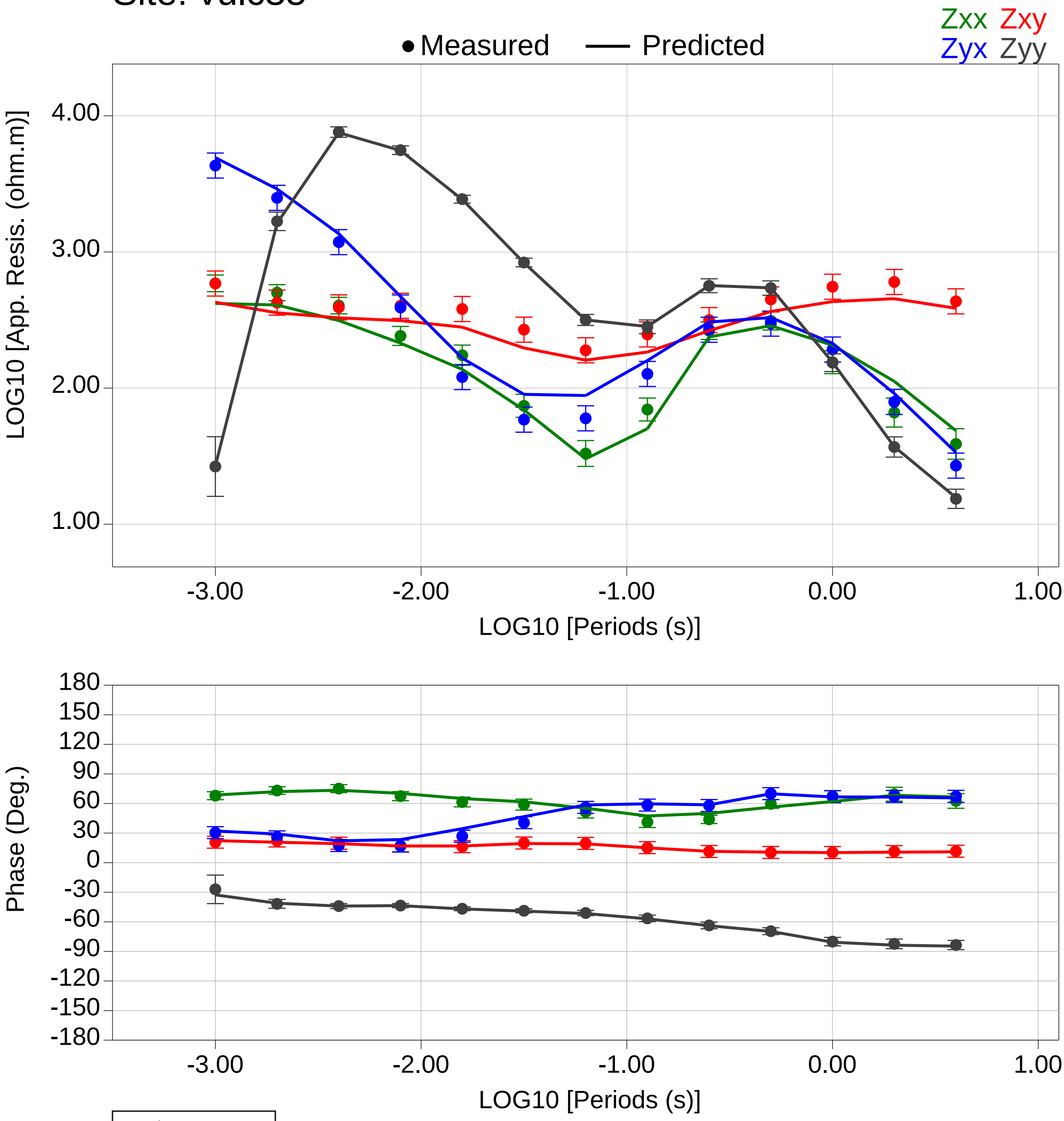

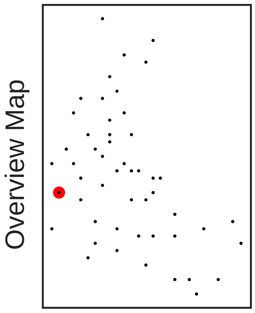

Overall RMS (Z+Tz)= 0.58
Total Z RMS = 0.58
Zxx RMS = 0.78
Zxy RMS = 0.54
Zyx RMS = 0.68
Zyy RMS = 0.10

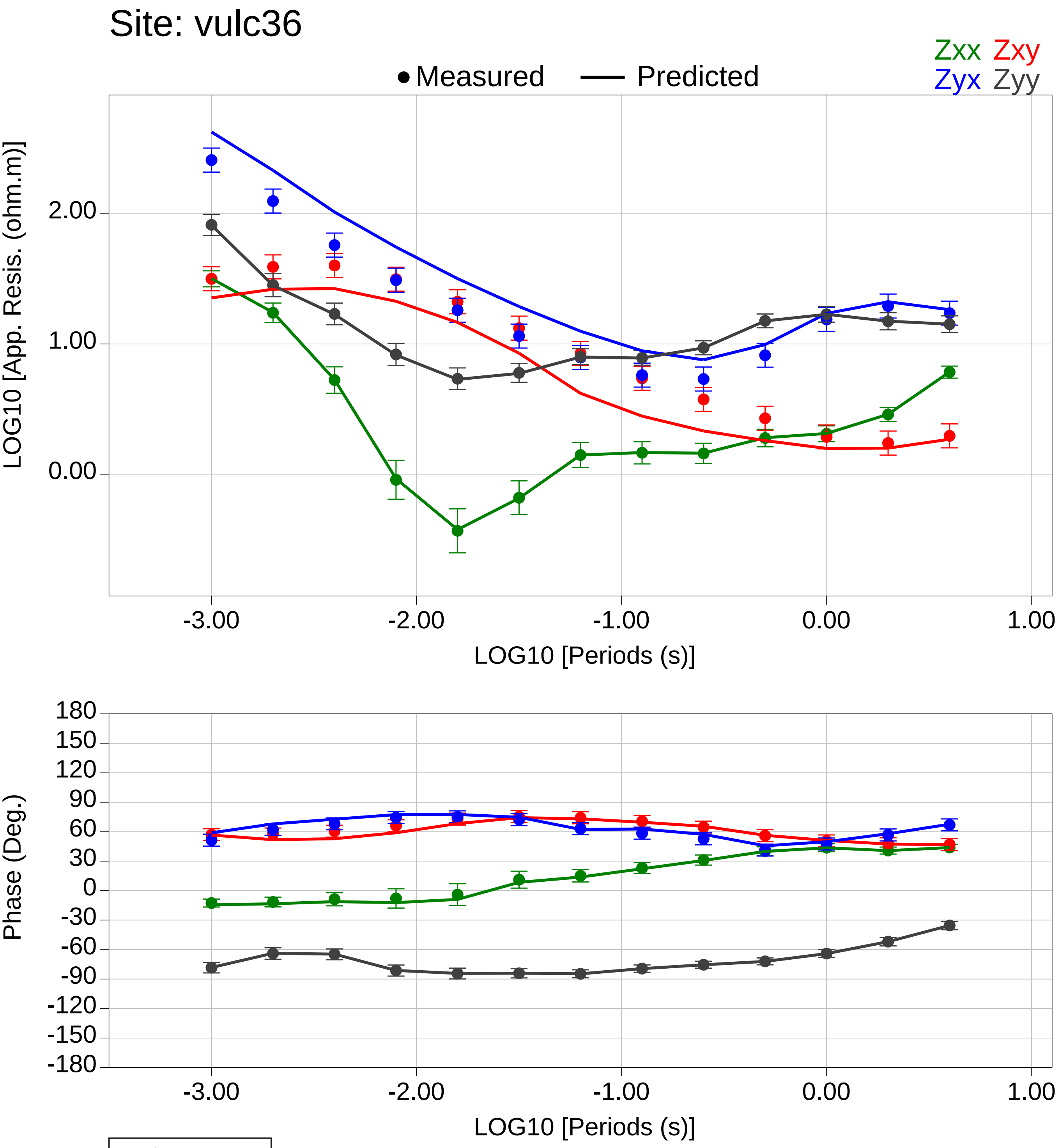

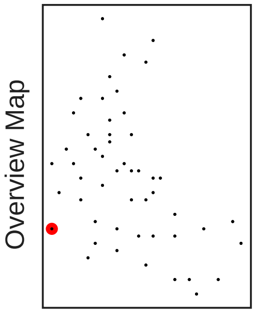

Overall RMS (Z+Tz)= 0.76
Total Z RMS = 0.76
Zxx RMS = 0.14
Zxy RMS = 0.93
Zyx RMS = 1.21
Zyy RMS = 0.02

Site: vulc37

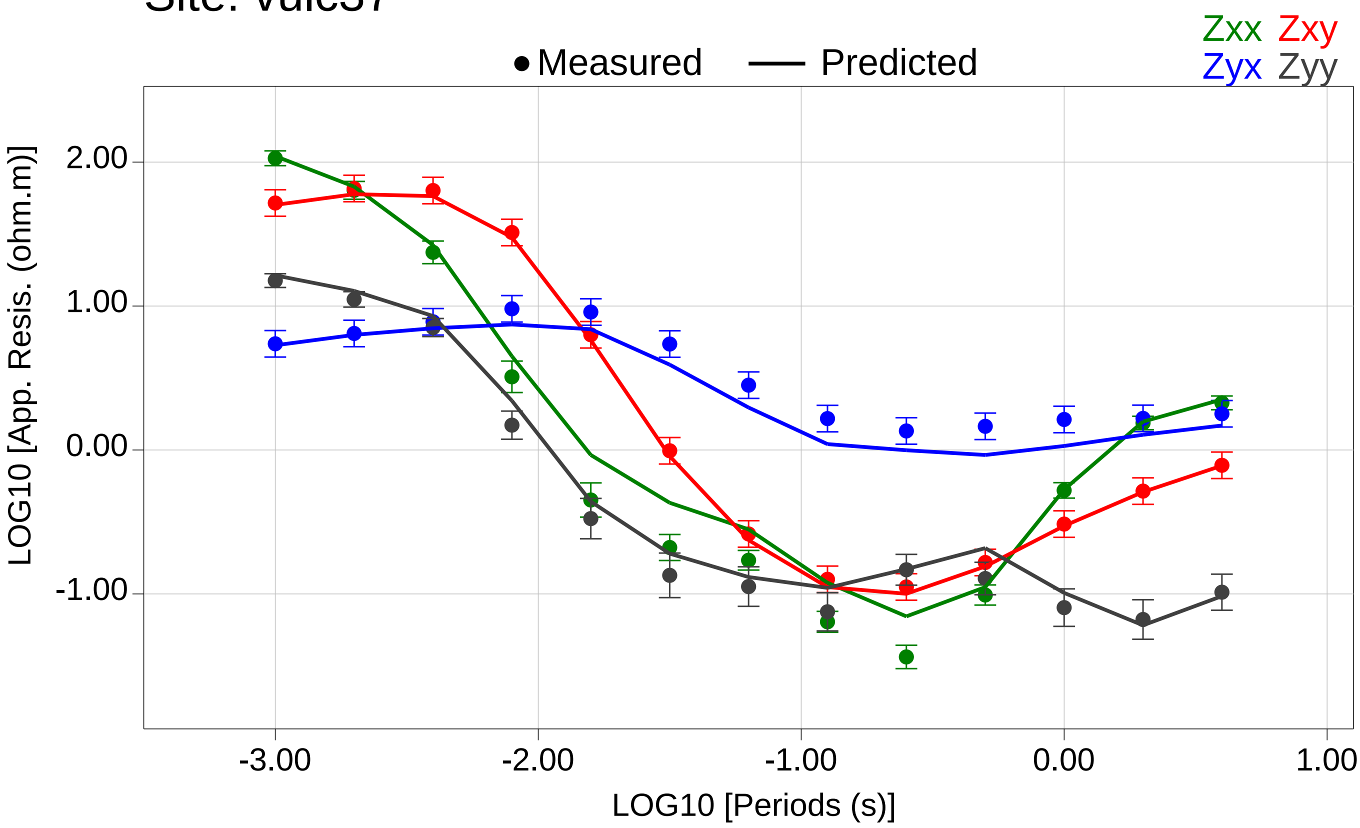

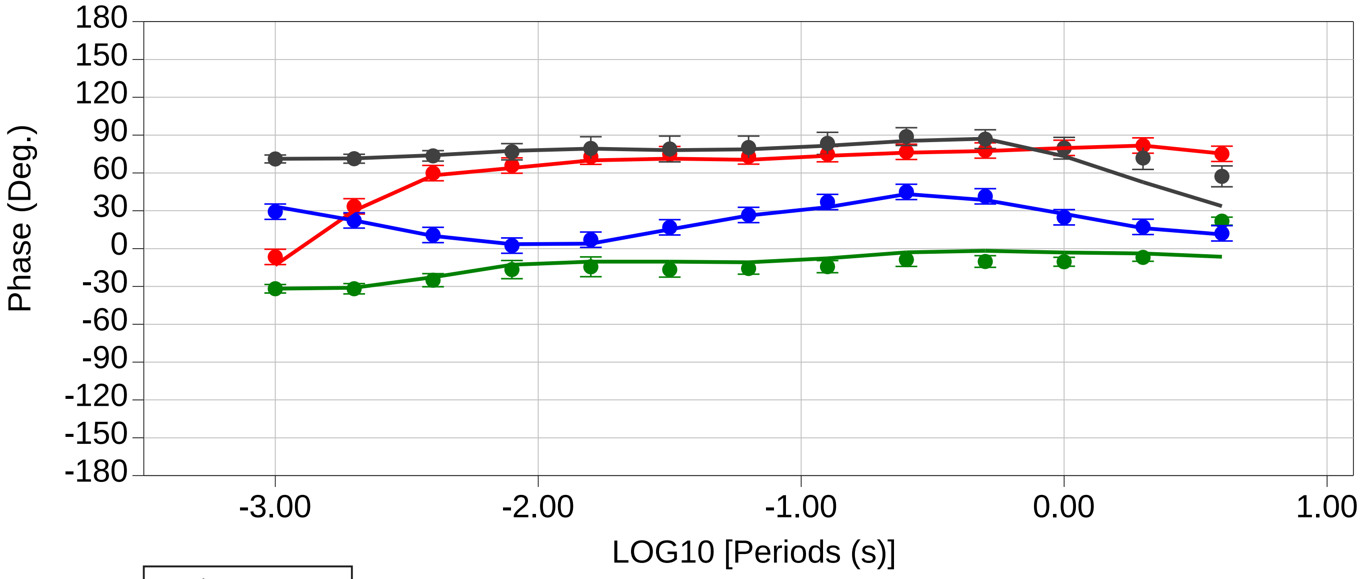

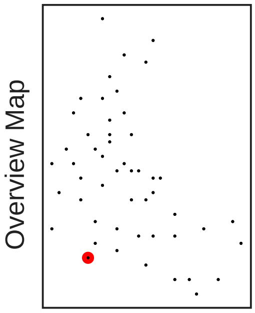

Overall RMS (Z+Tz)= 1.06
Total Z RMS = 1.06
Zxx RMS = 1.85
Zxy RMS = 0.28
Zyx RMS = 0.66
Zyy RMS = 0.76

Site: vulc38

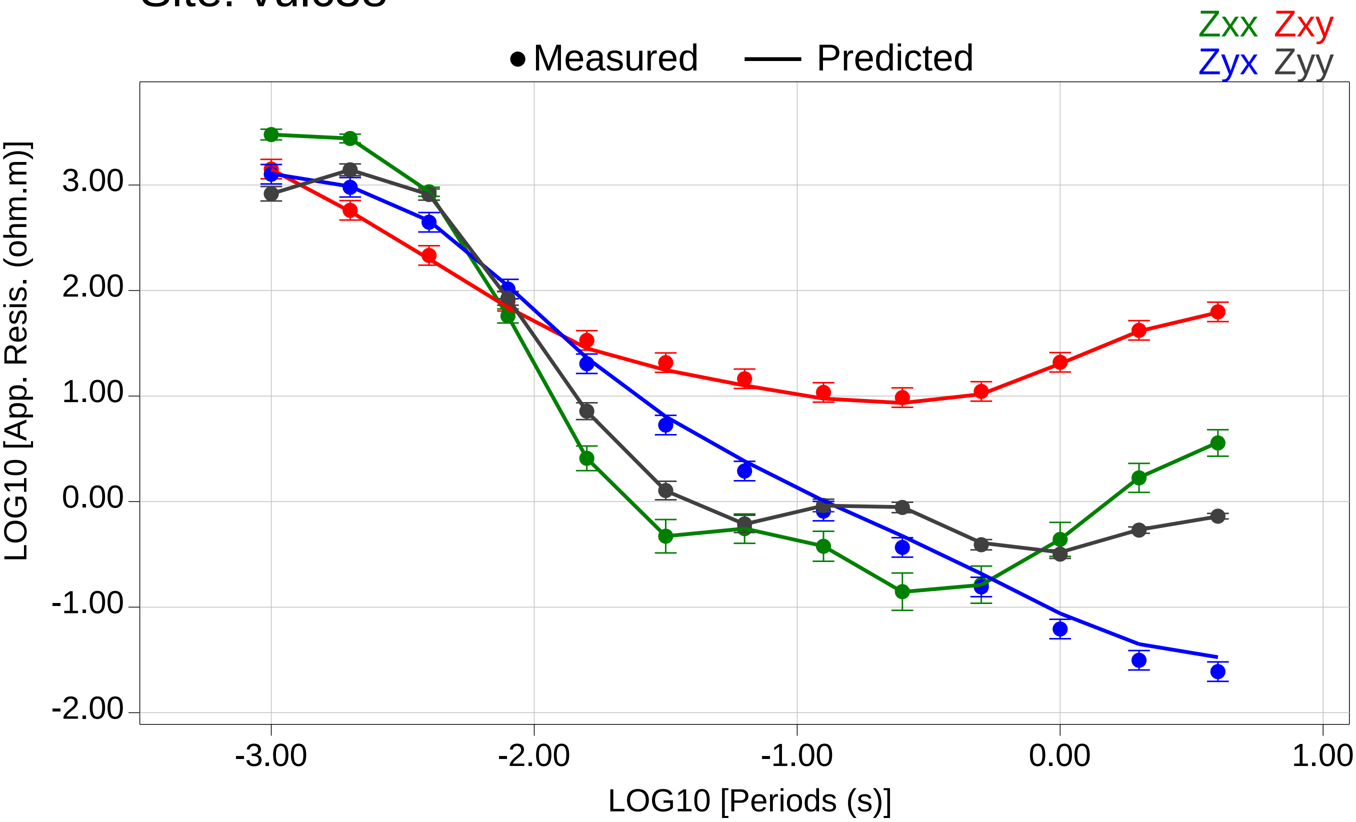

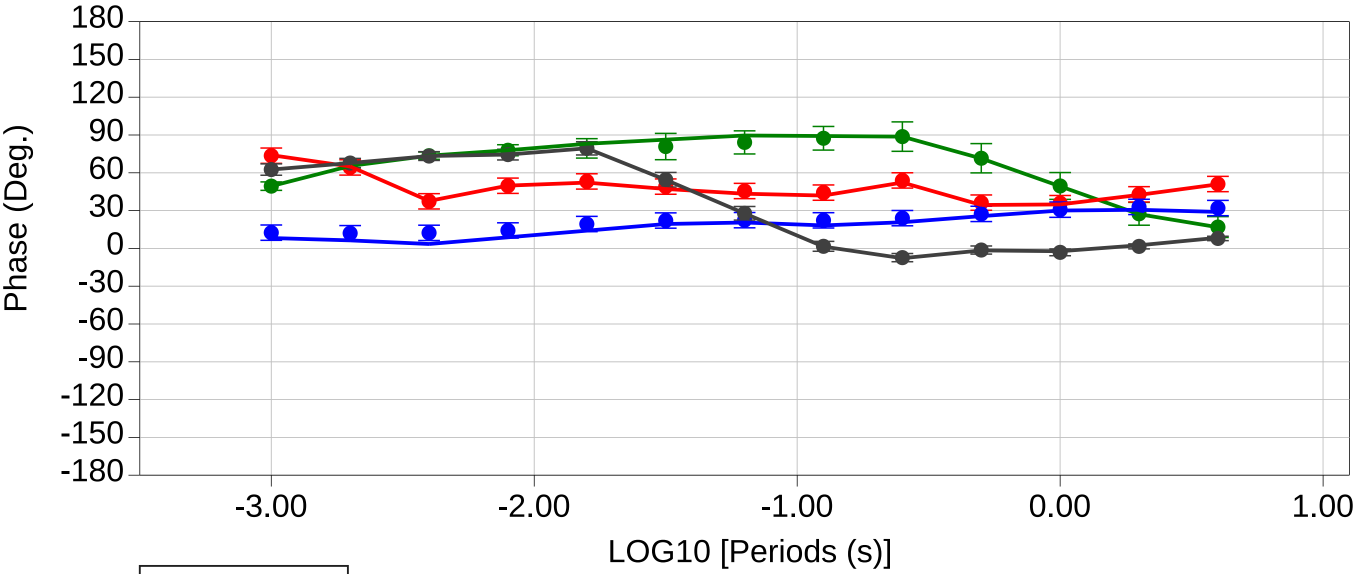

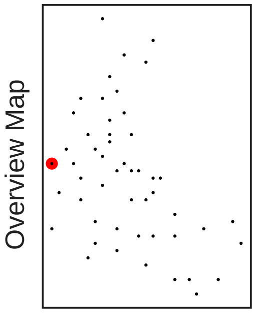

Overall RMS (Z+Tz)= 0.37
Total Z RMS = 0.37
Zxx RMS = 0.13
Zxy RMS = 0.26
Zyx RMS = 0.67
Zyy RMS = 0.12

Site: vulc39

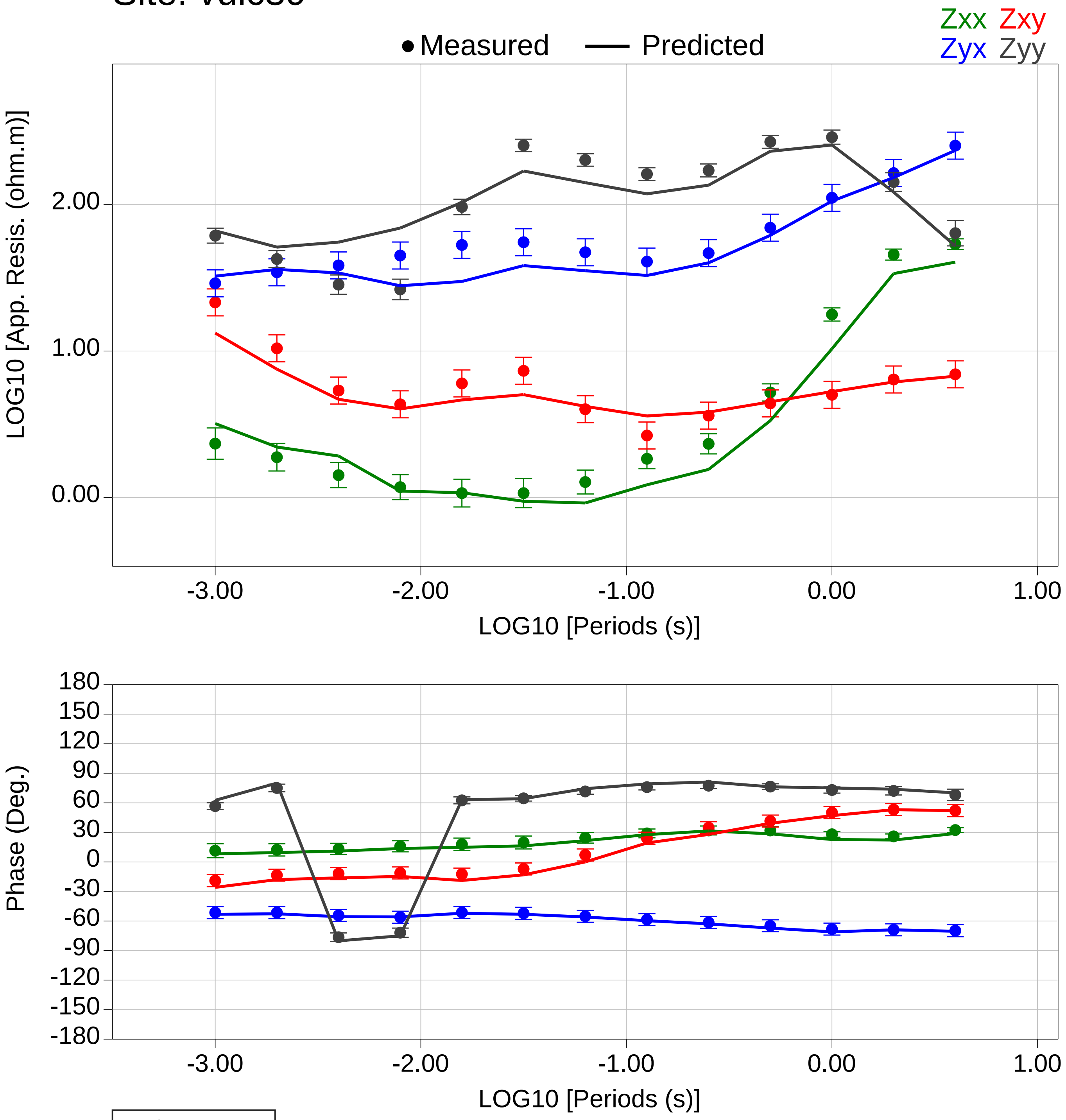

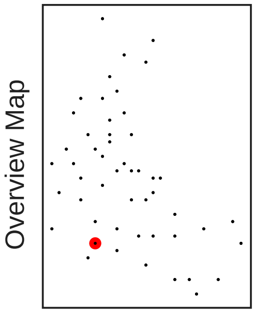

Overall RMS (Z+Tz)= 1.11
Total Z RMS = 1.11
Zxx RMS = 1.22
Zxy RMS = 0.64
Zyx RMS = 0.57
Zyy RMS = 1.66

Site: vulc40

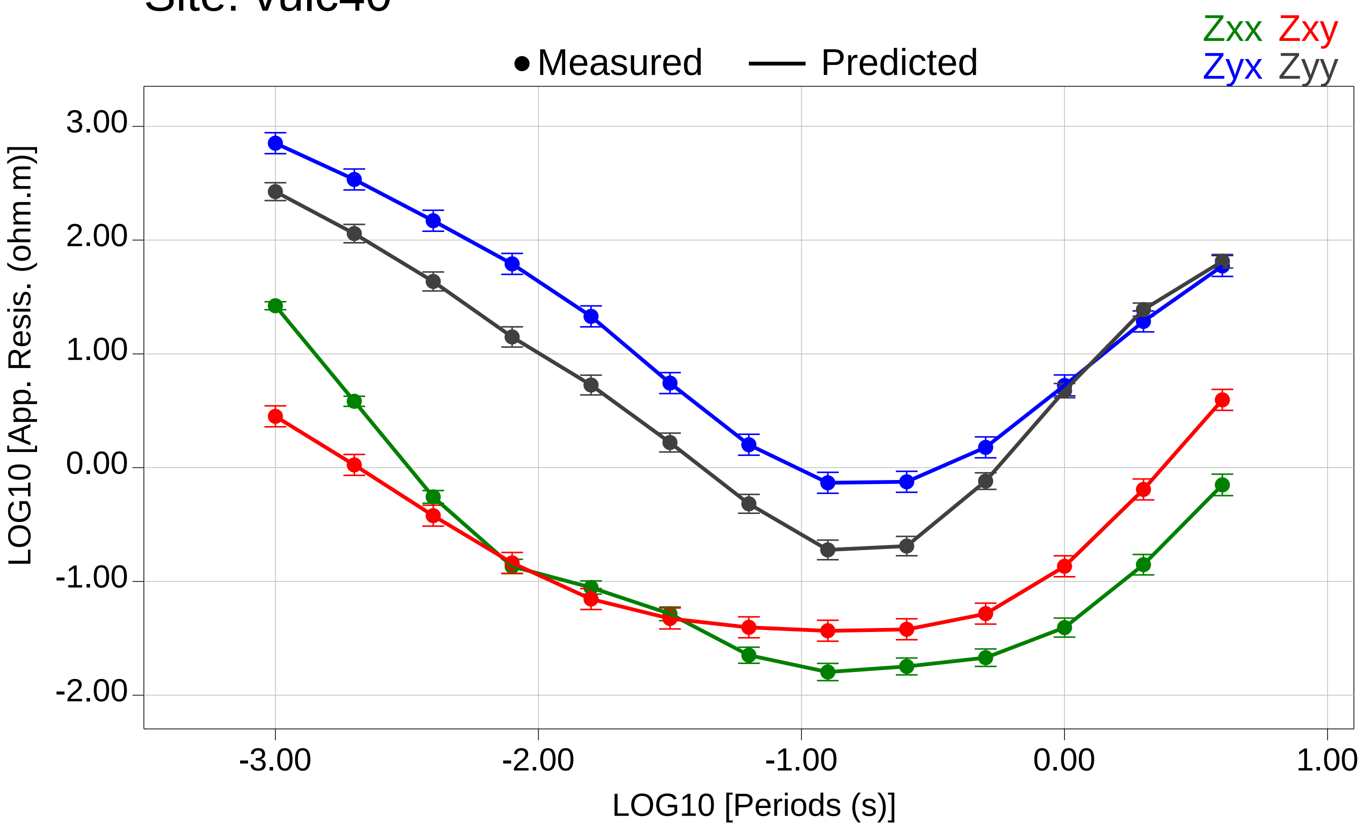

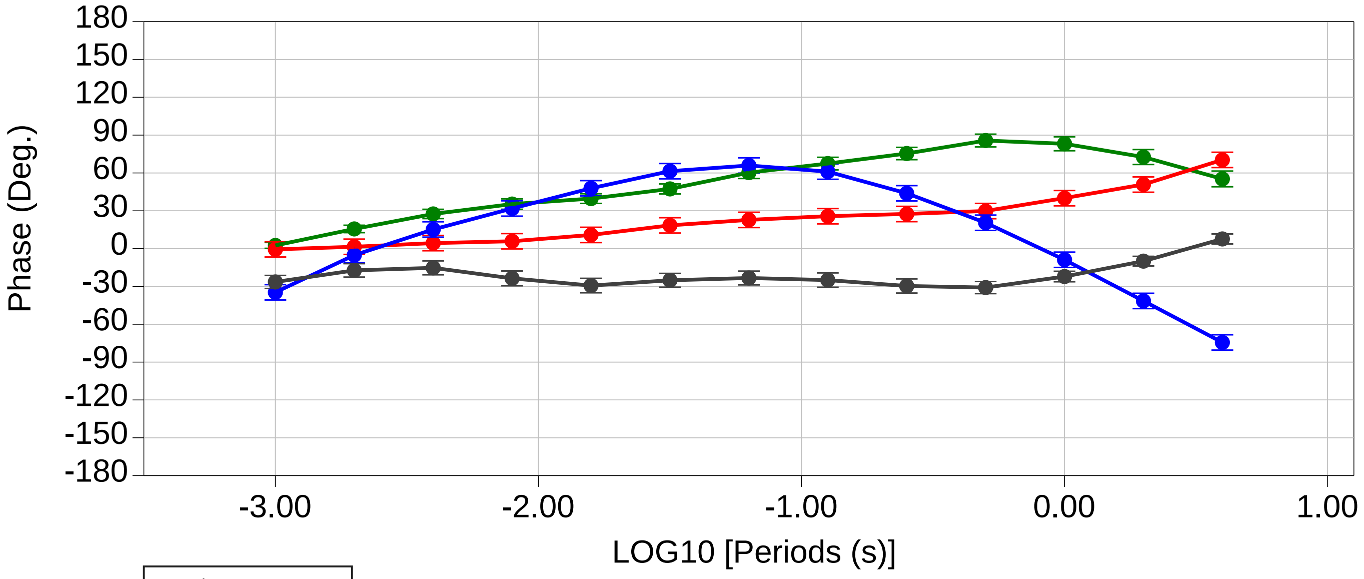

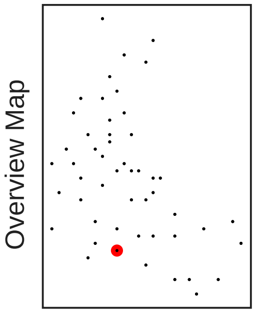

Overall RMS (Z+Tz)= 0.00
Total Z RMS = 0.00
Zxx RMS = 0.00
Zxy RMS = 0.01
Zyx RMS = 0.00
Zyy RMS = 0.00

Site: vulc41

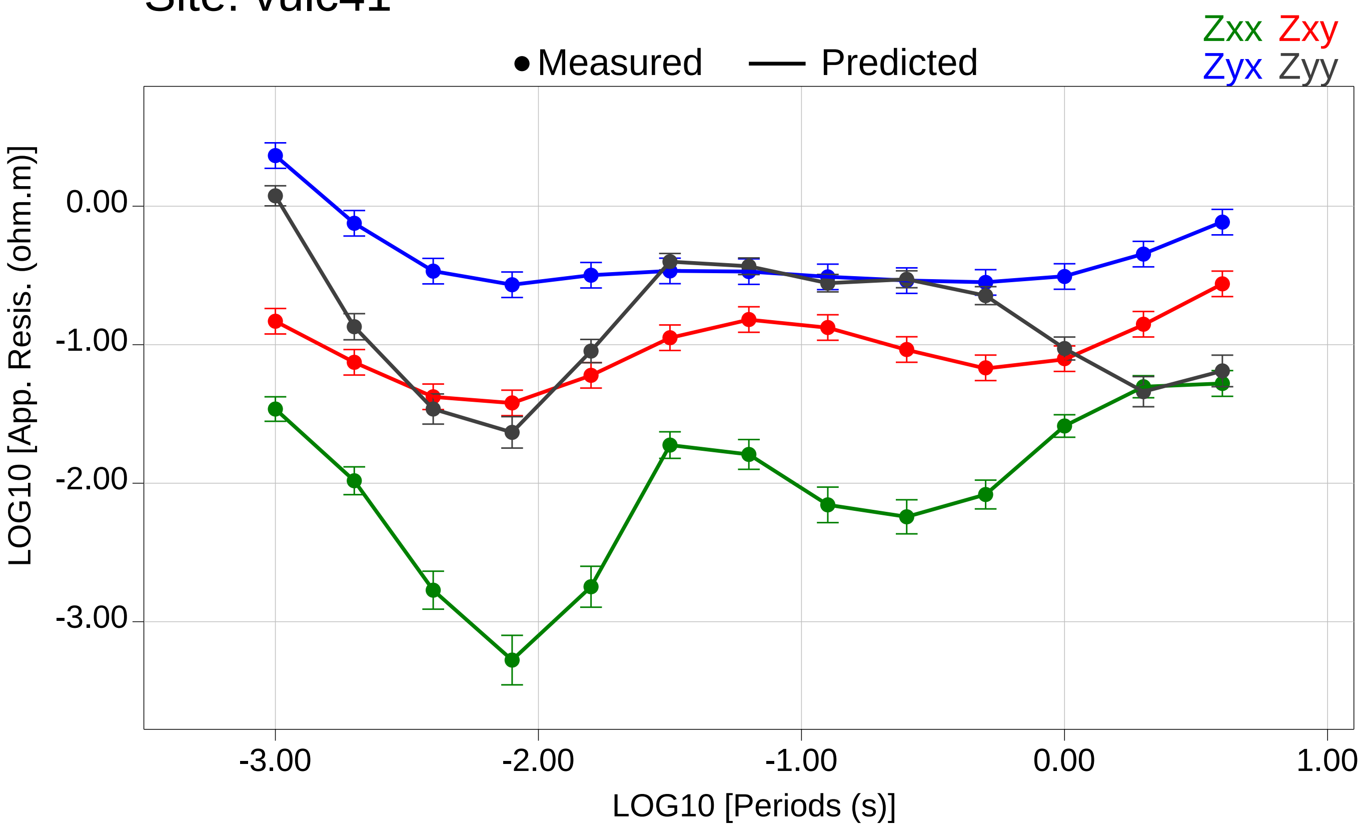

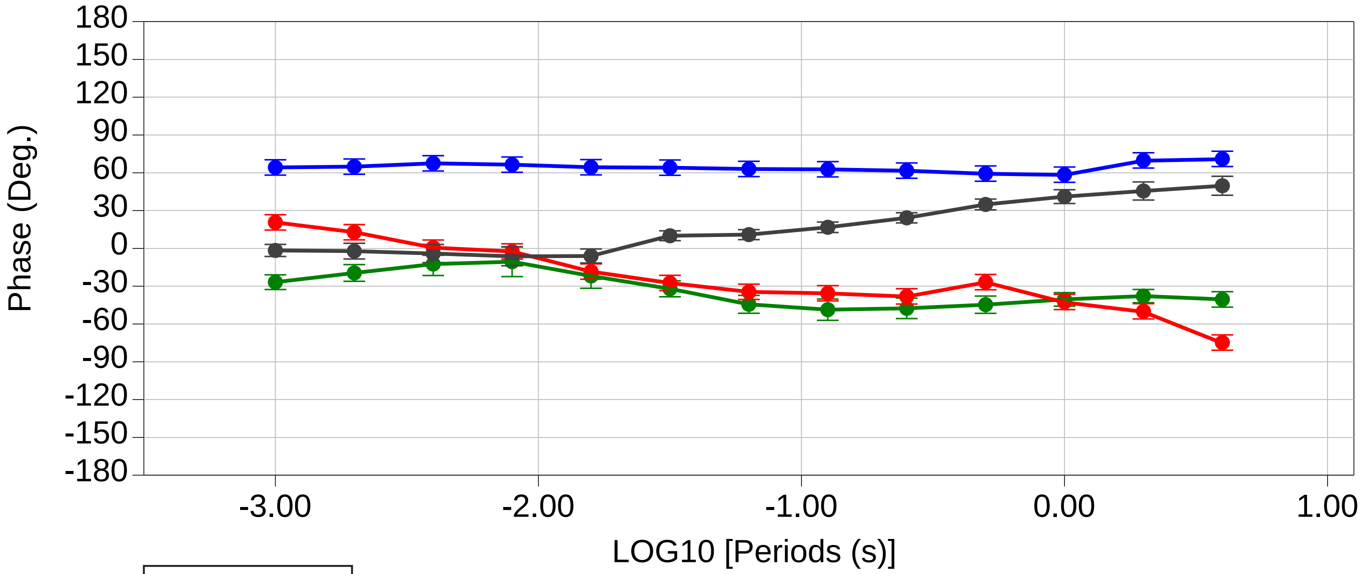

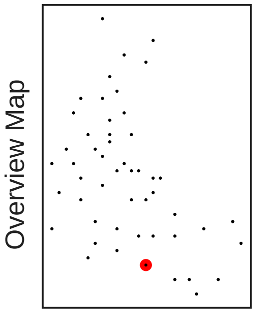

Overall RMS (Z+Tz)= 0.01
Total Z RMS = 0.01
Zxx RMS = 0.00
Zxy RMS = 0.02
Zyx RMS = 0.01
Zyy RMS = 0.00

Site: vulc42

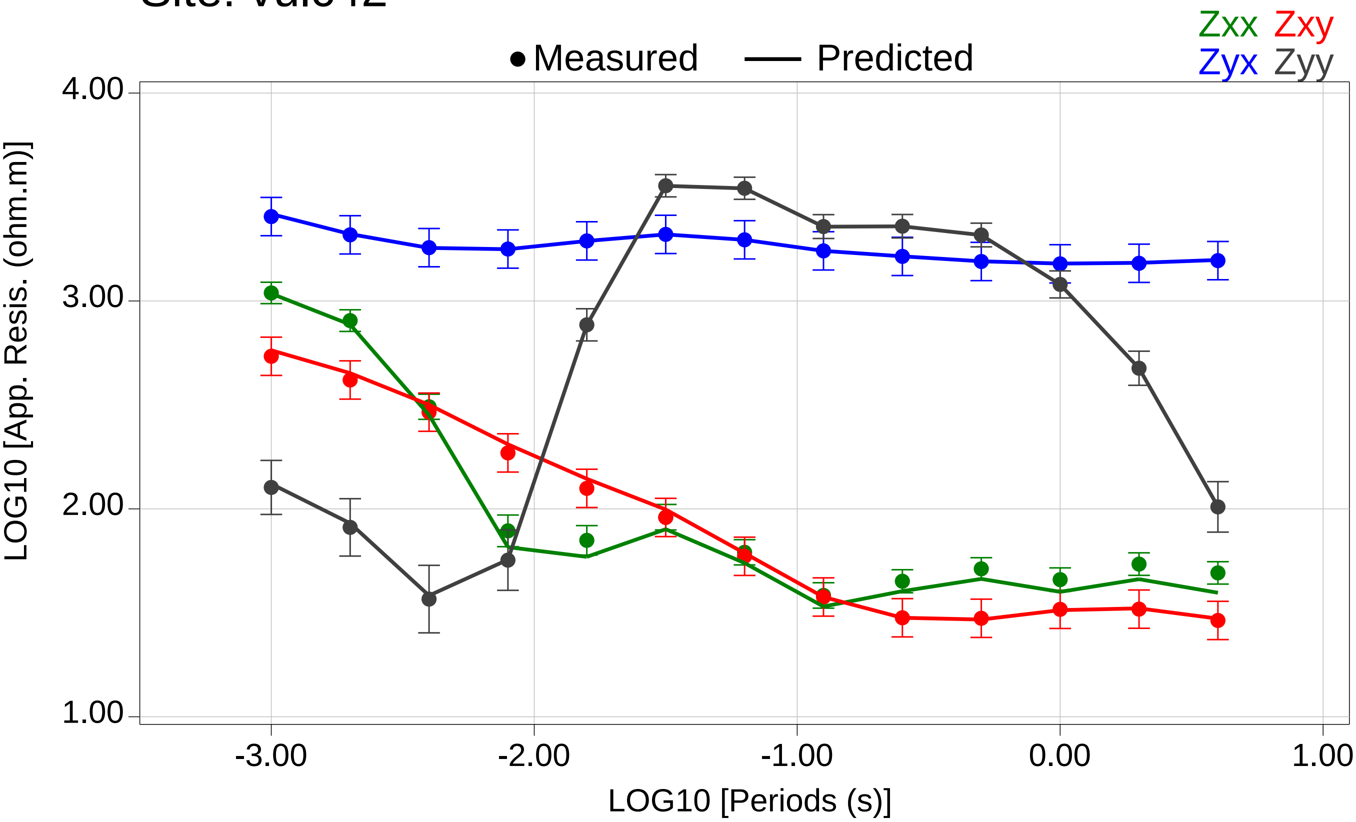

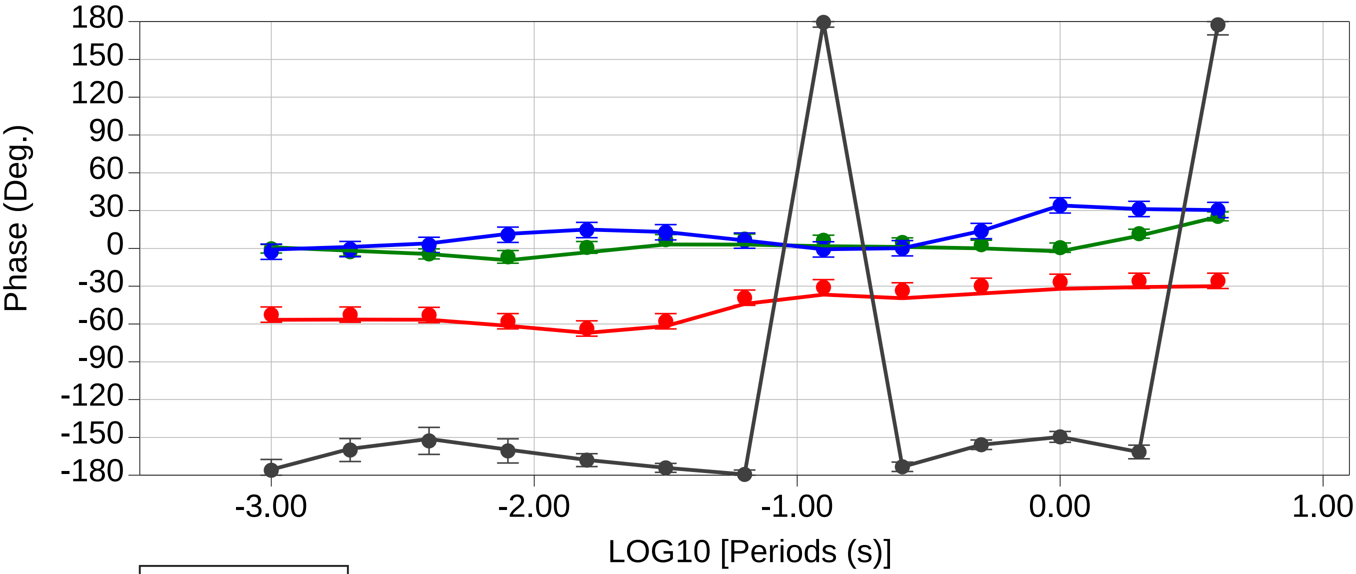

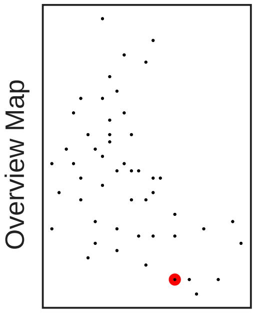

Overall RMS (Z+Tz)= 0.37
Total Z RMS = 0.37
Zxx RMS = 0.60
Zxy RMS = 0.42
Zyx RMS = 0.07
Zyy RMS = 0.04

Site: vulc43

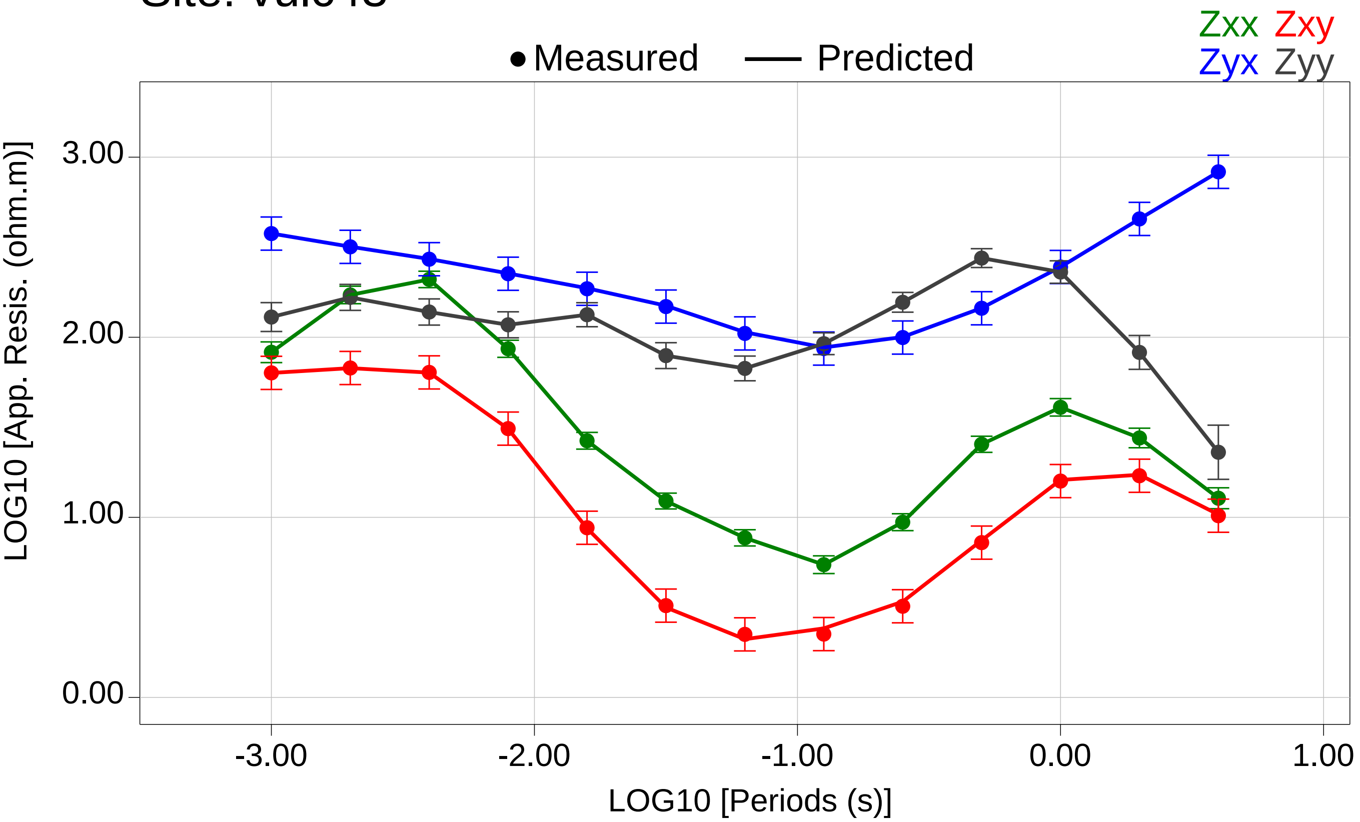

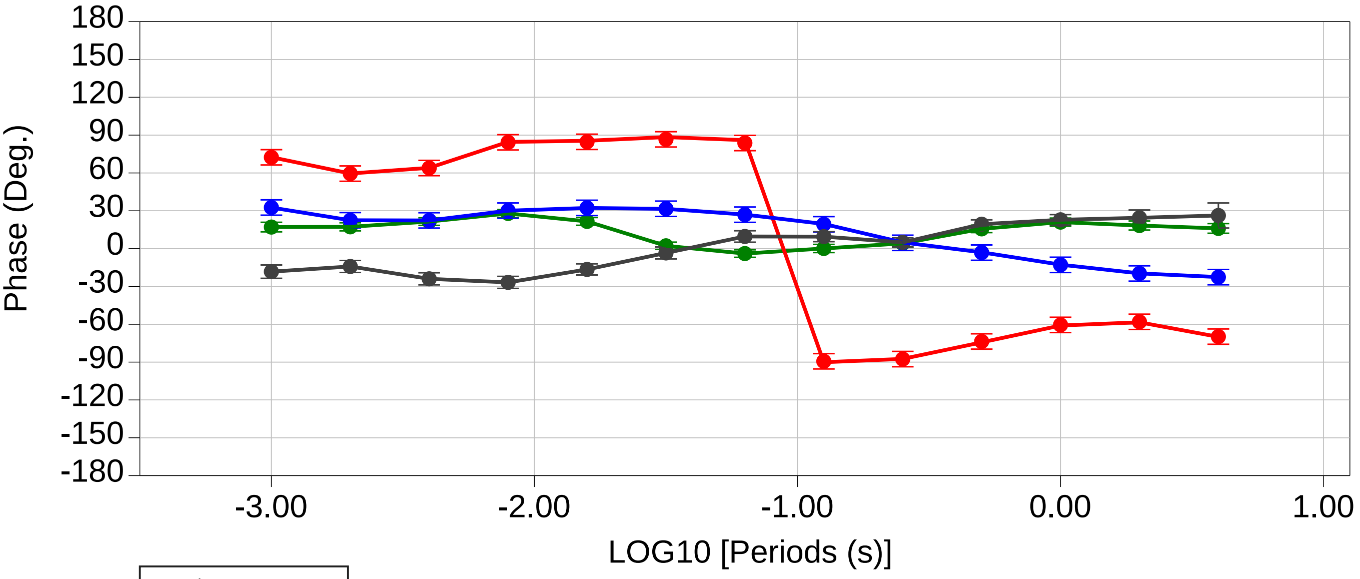

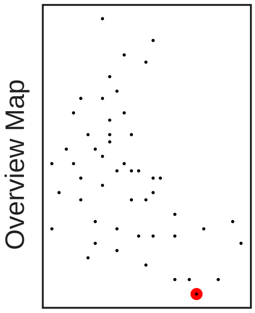

Overall RMS (Z+Tz)= 0.06
Total Z RMS = 0.06
Zxx RMS = 0.00
Zxy RMS = 0.11
Zyx RMS = 0.02
Zyy RMS = 0.00

Site: vulc44

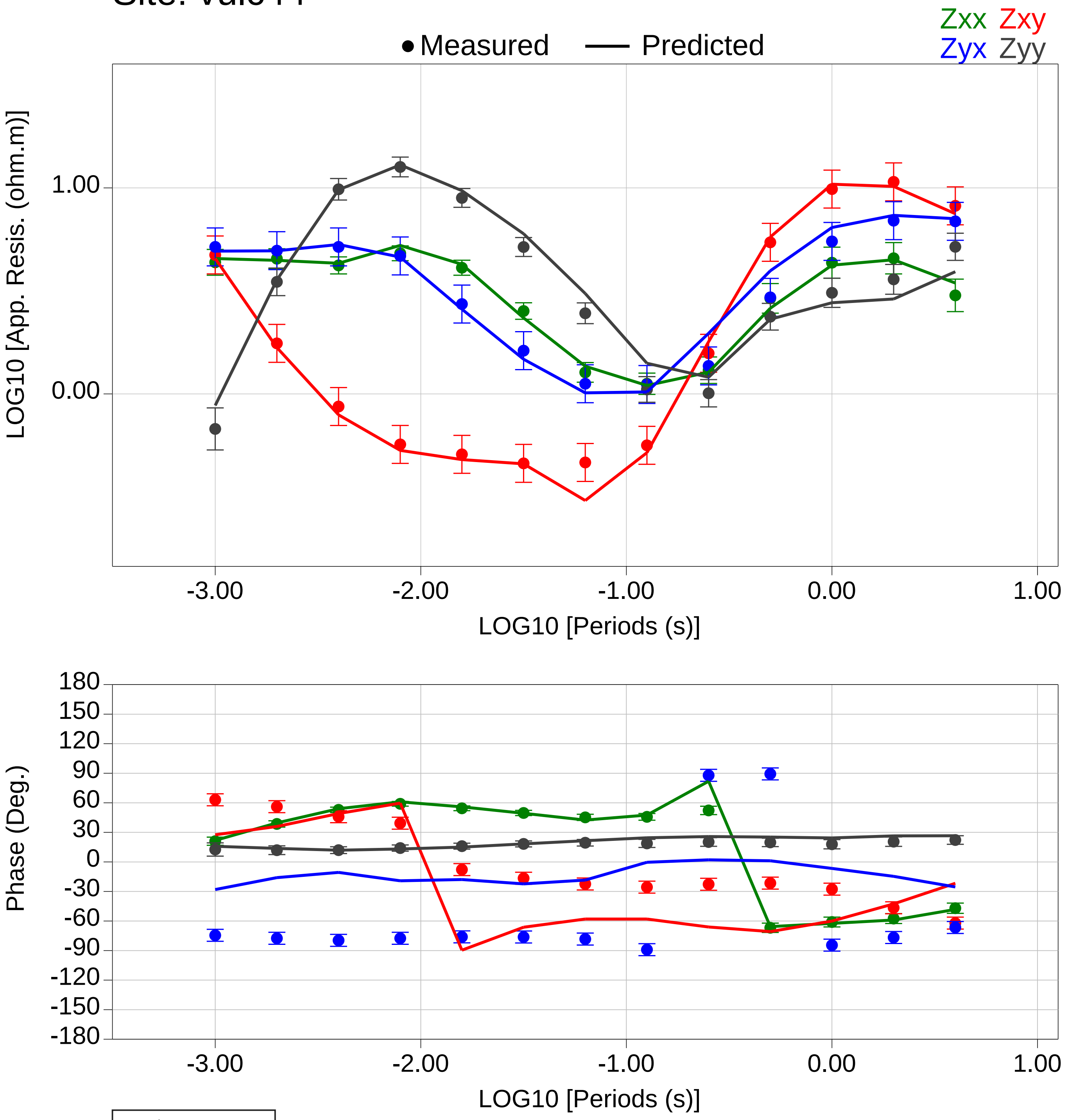

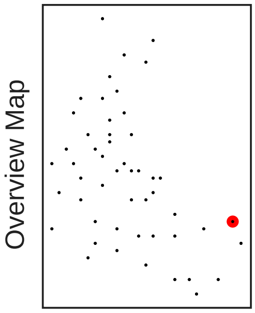

Overall RMS (Z+Tz)= 3.13
Total Z RMS = 3.13
Zxx RMS = 1.01
Zxy RMS = 3.12
Zyx RMS = 5.28
Zyy RMS = 0.76

Site: vulc45

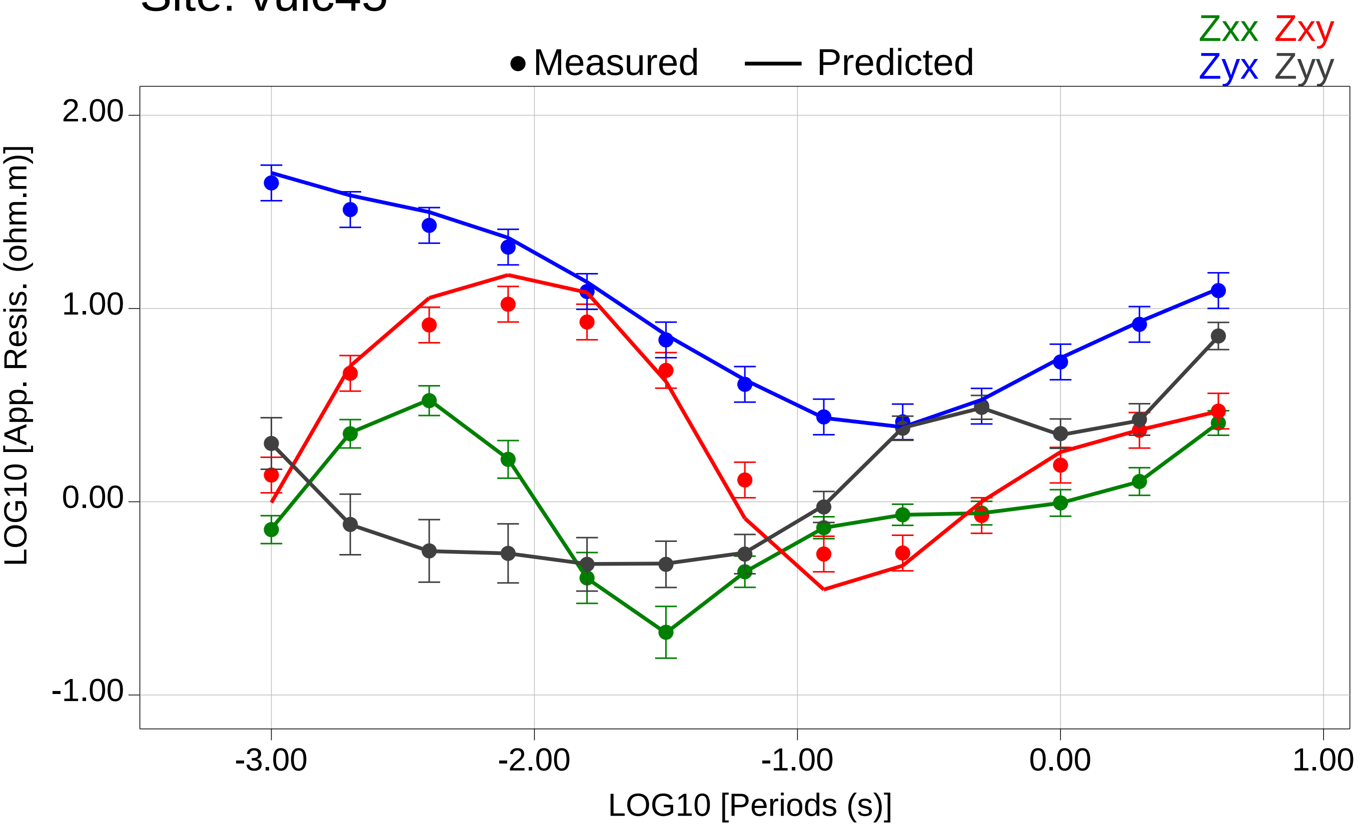

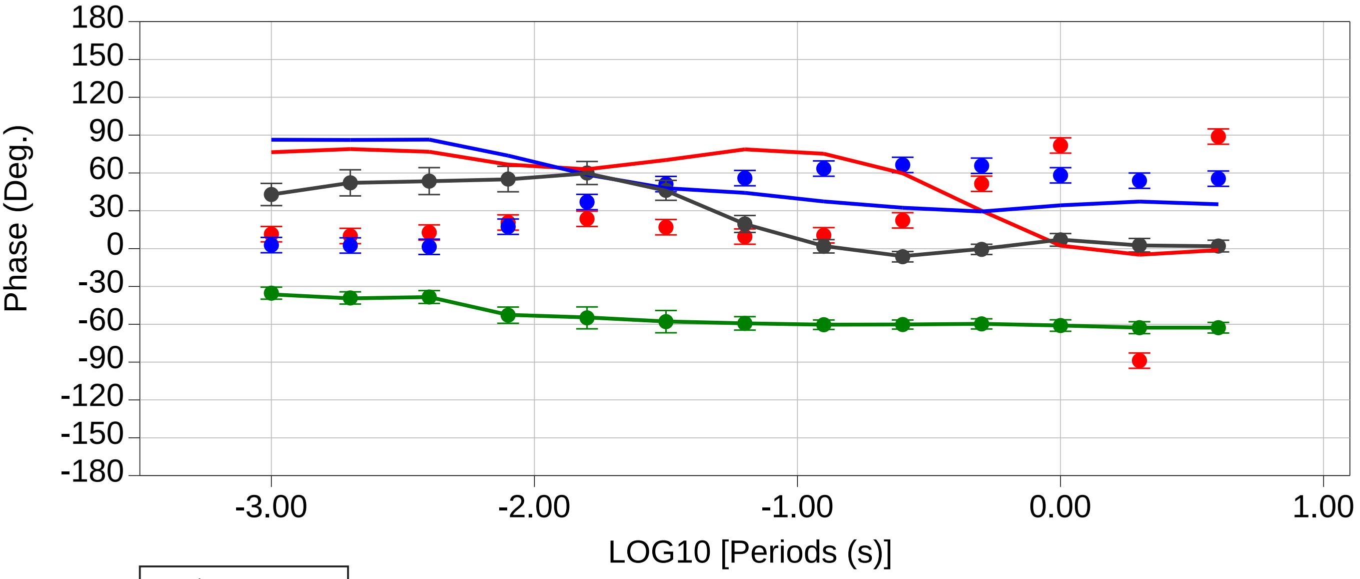

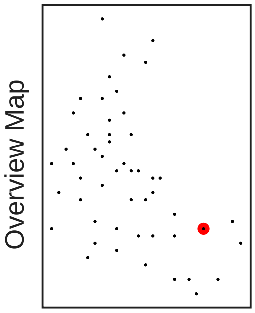

Overall RMS (Z+Tz)= 3.08
Total Z RMS = 3.08
Zxx RMS = 0.04
Zxy RMS = 4.88
Zyx RMS = 3.76
Zyy RMS = 0.03

Site: vulc46

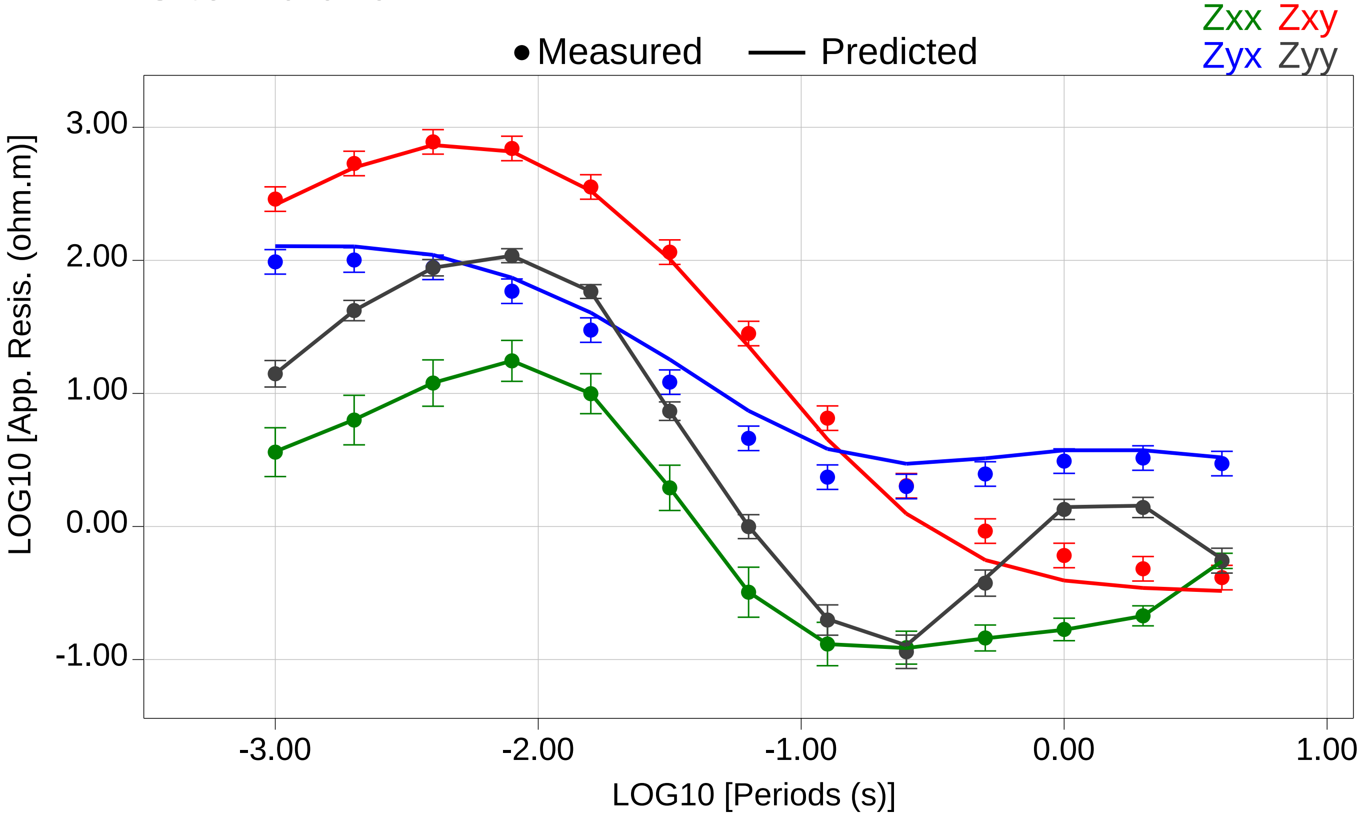

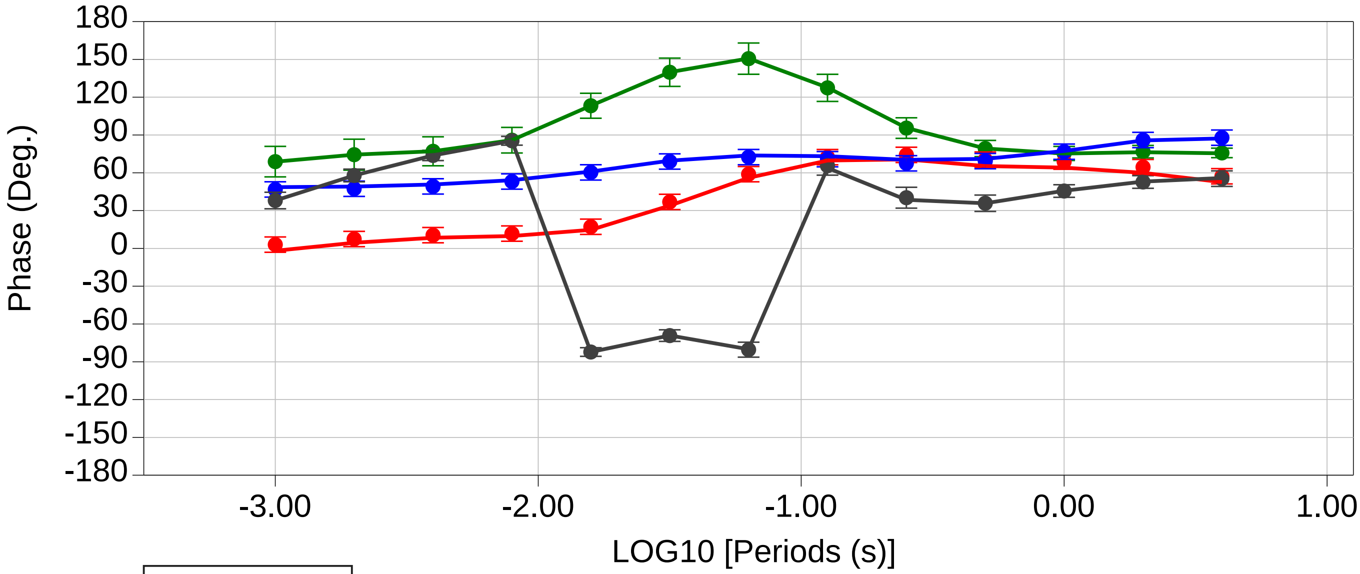

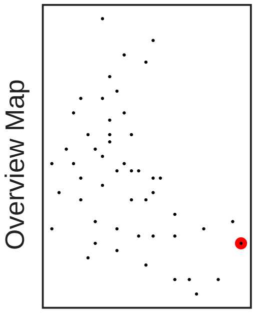

Overall RMS (Z+Tz)= 0.53
Total Z RMS = 0.53
Zxx RMS = 0.02
Zxy RMS = 0.67
Zyx RMS = 0.81
Zyy RMS = 0.10

Site: vulc47

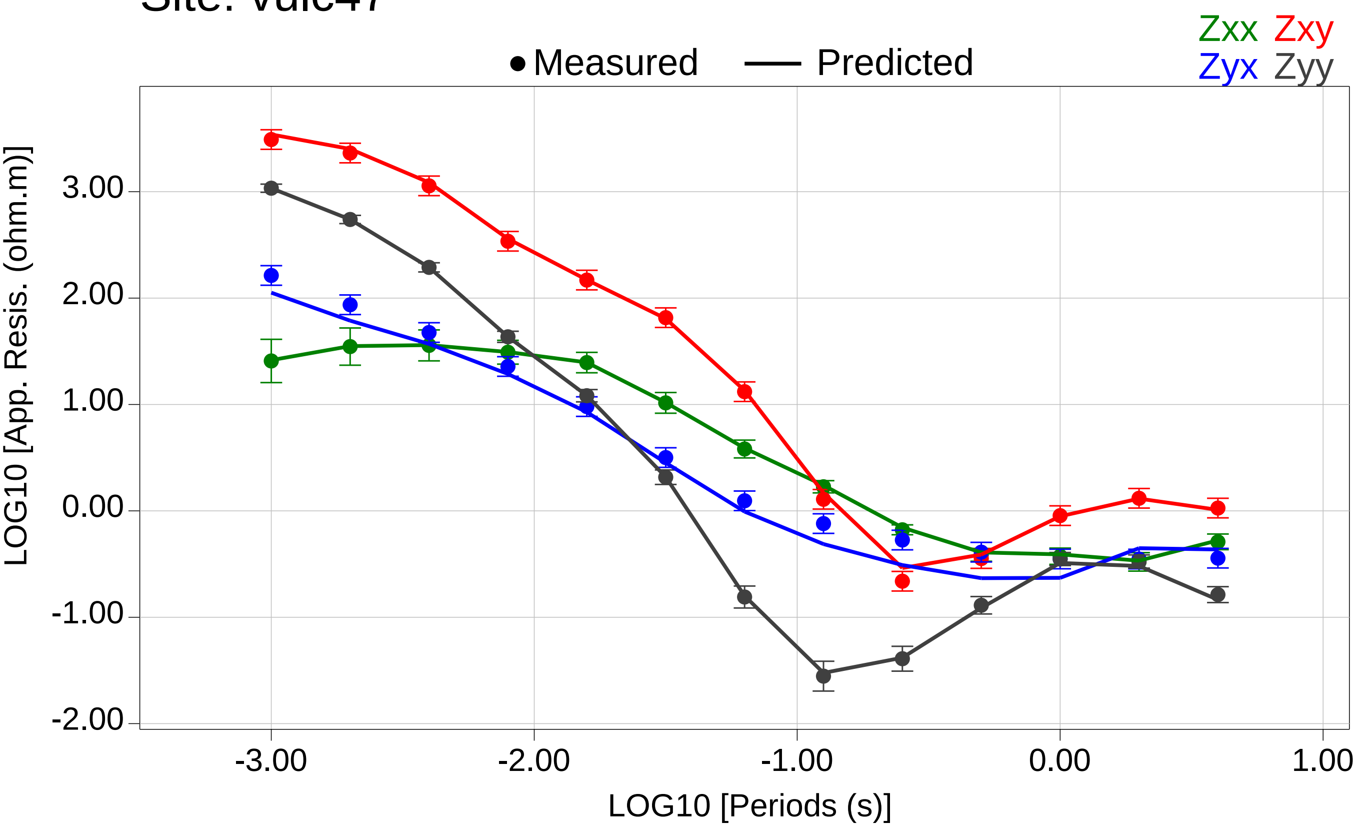

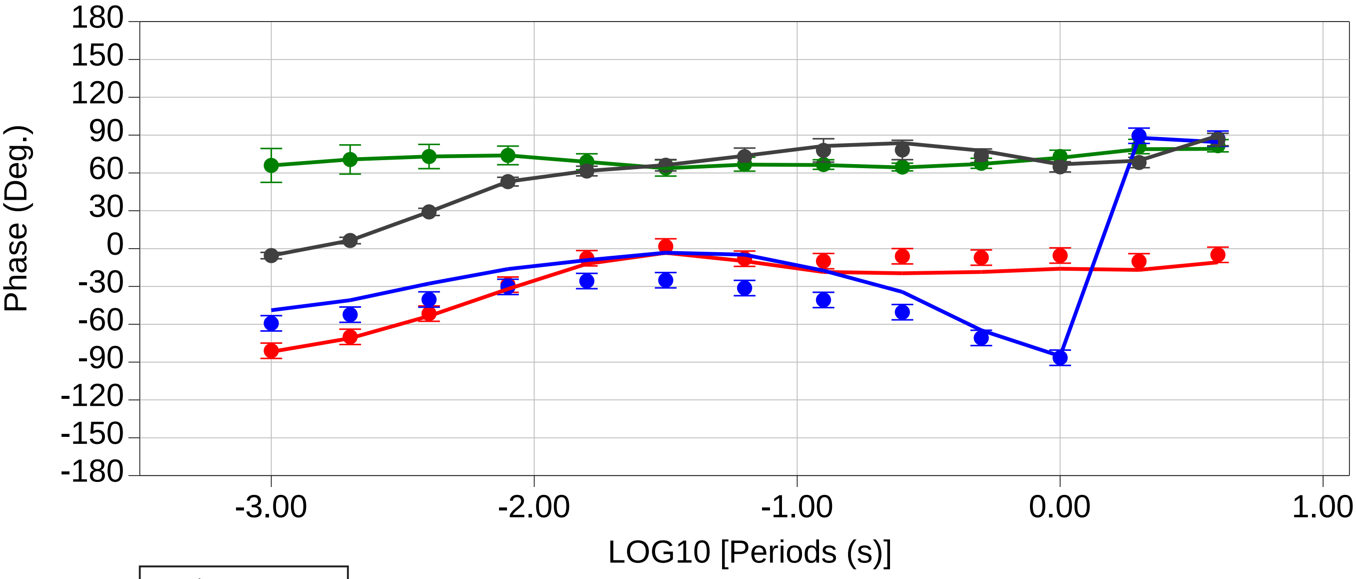

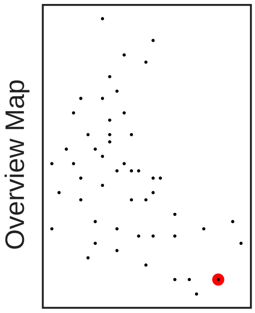

Overall RMS (Z+Tz)= 0.76
Total Z RMS = 0.76
Zxx RMS = 0.14
Zxy RMS = 0.65
Zyx RMS = 1.35
Zyy RMS = 0.25

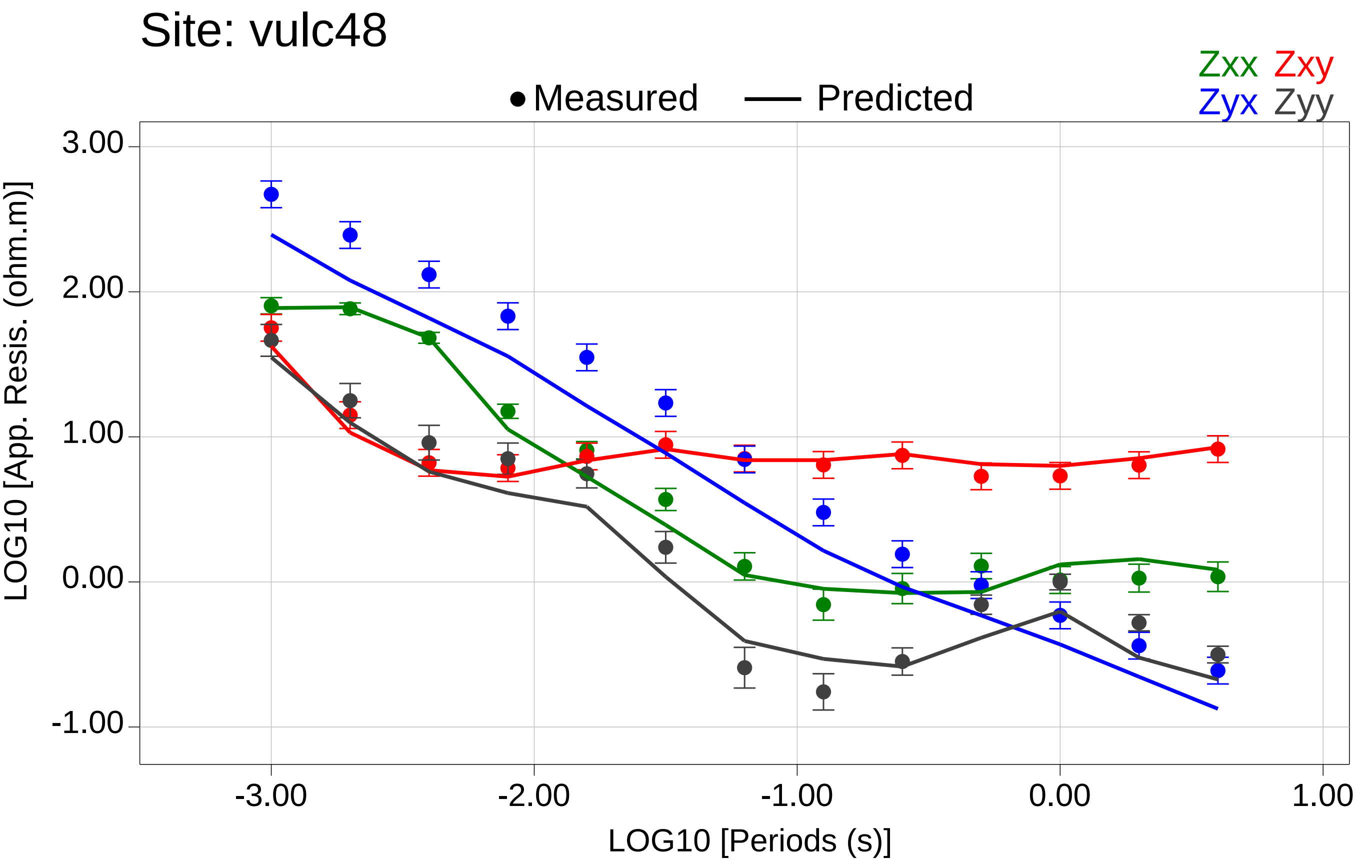

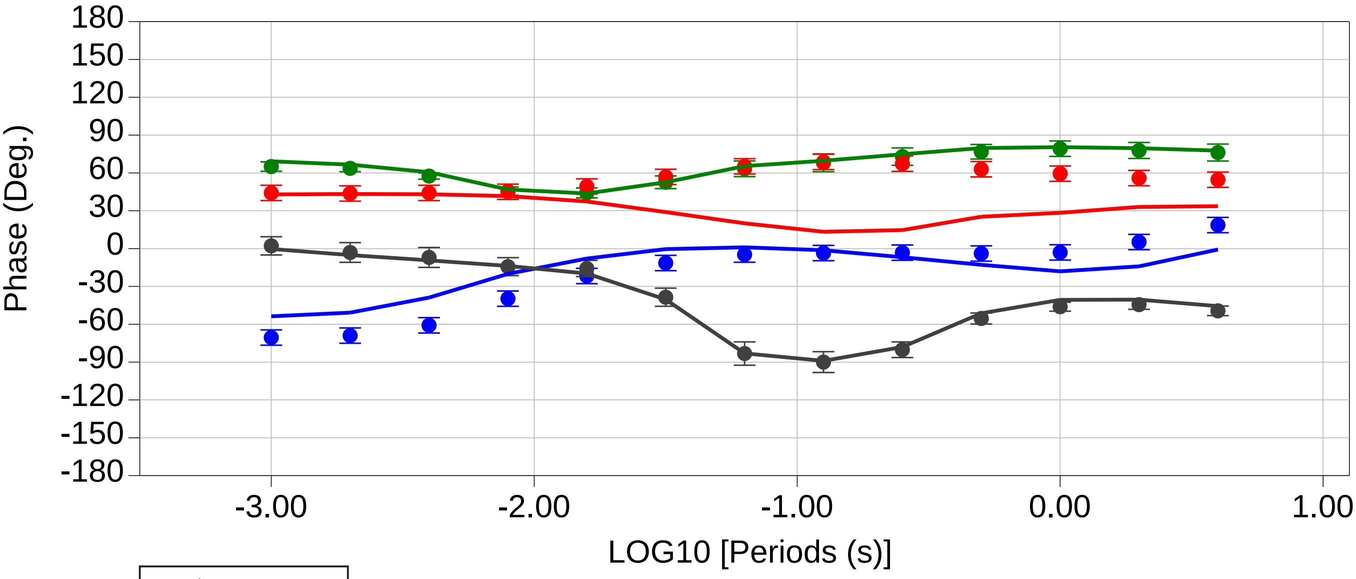

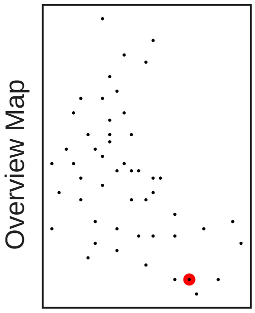

Overall RMS (Z+Tz)= 1.65
Total Z RMS = 1.65
Zxx RMS = 0.78
Zxy RMS = 2.51
Zyx RMS = 1.65
Zyy RMS = 1.15

Site: vulc49

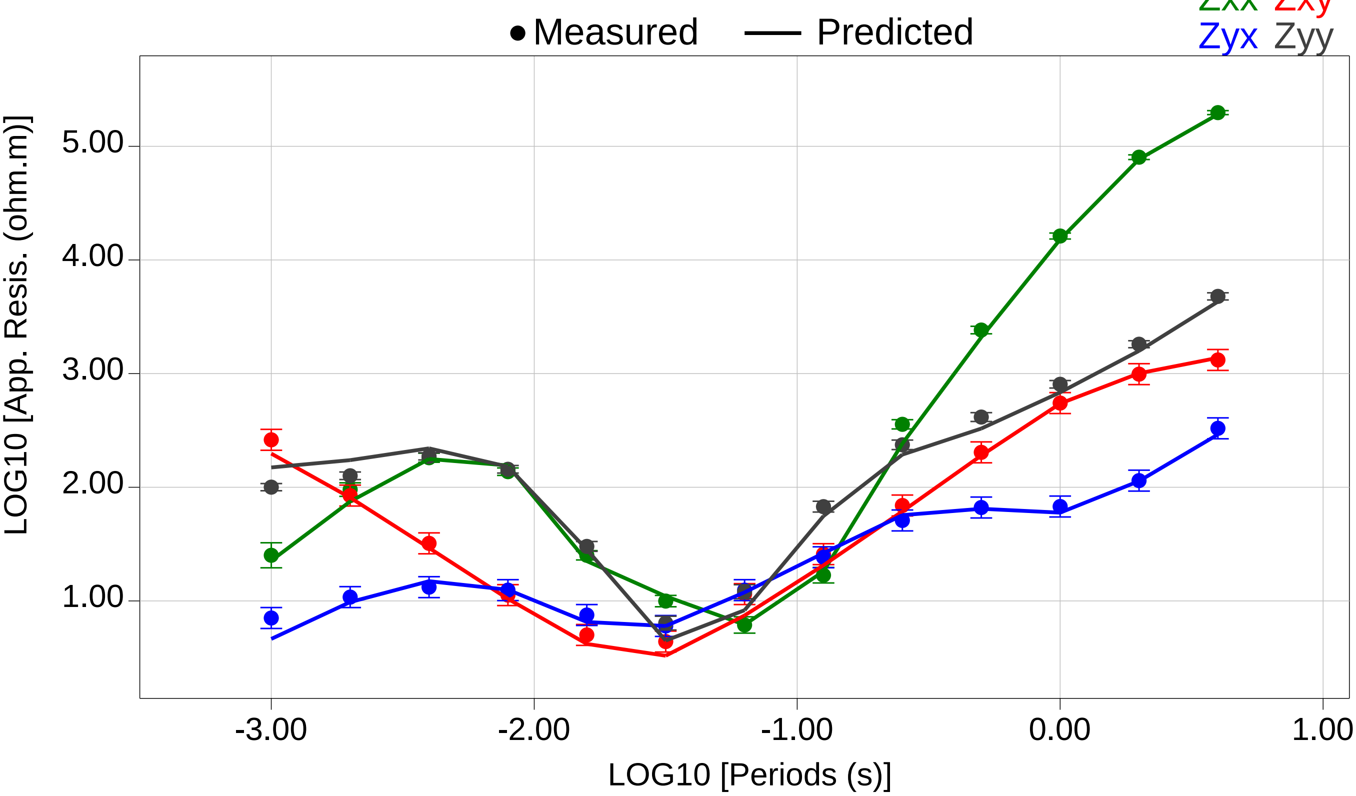

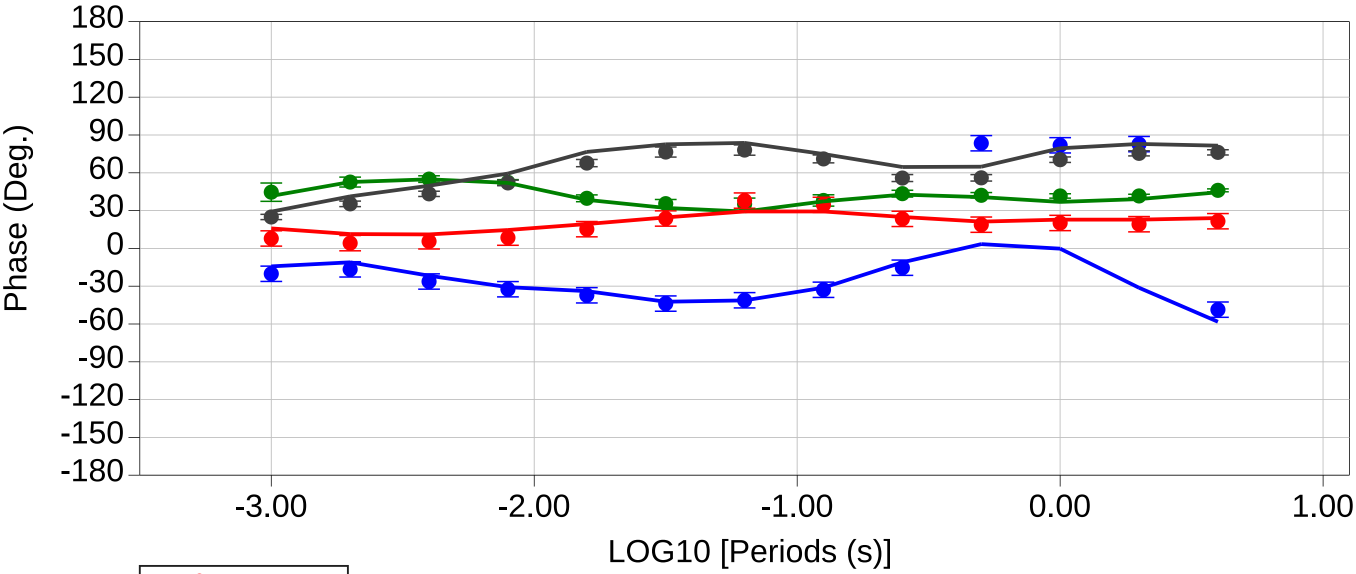

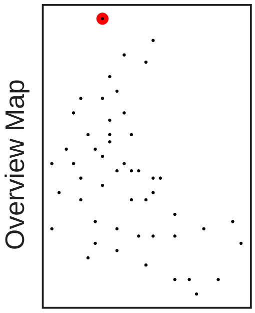

Overall RMS (Z+Tz)= 1.98
Total Z RMS = 1.98
Zxx RMS = 0.93
Zxy RMS = 0.57
Zyx RMS = 3.25
Zyy RMS = 1.97

Site: vulc50

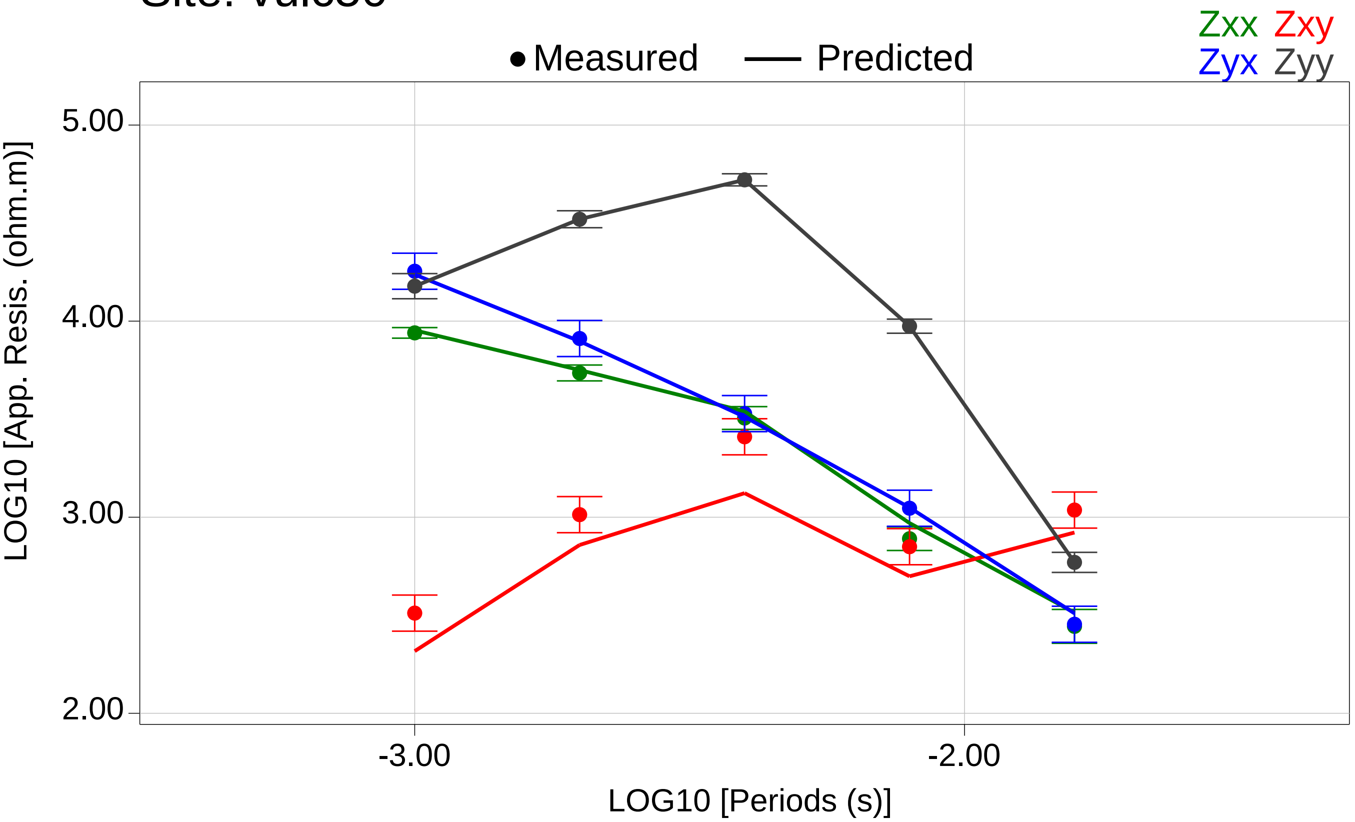

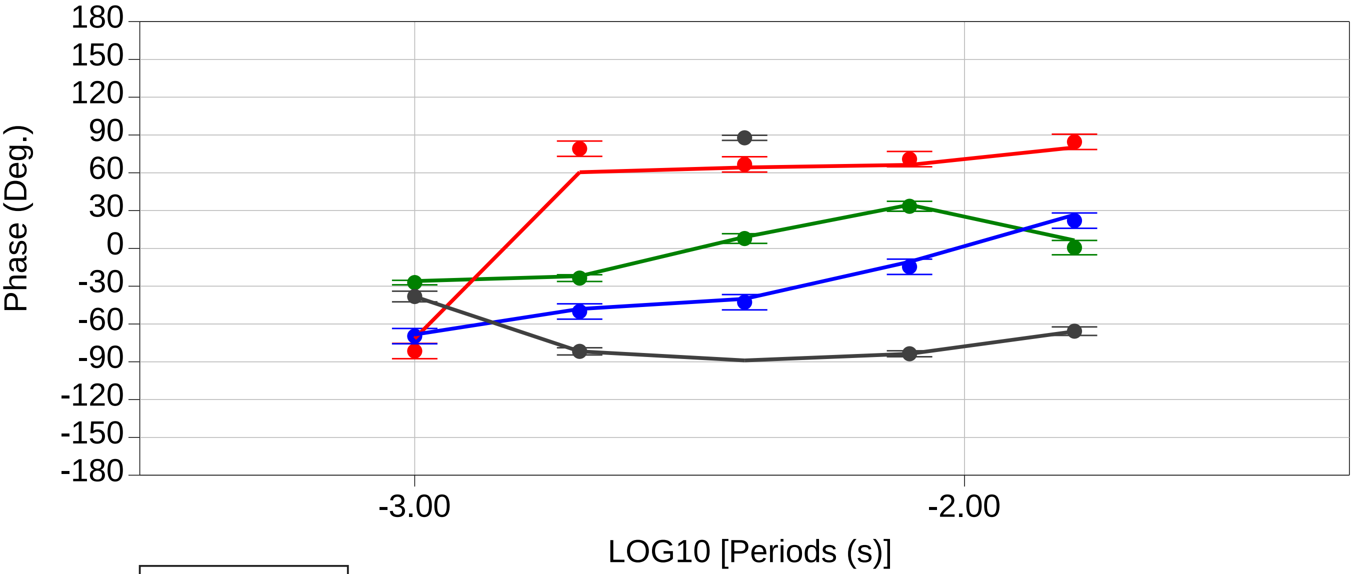

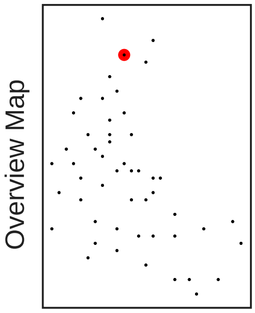

Overall RMS (Z+Tz)= 6.31
Total Z RMS = 6.31
Zxx RMS = 0.52
Zxy RMS = 1.18
Zyx RMS = 0.30
Zyy RMS = 12.56

Site: vulc51

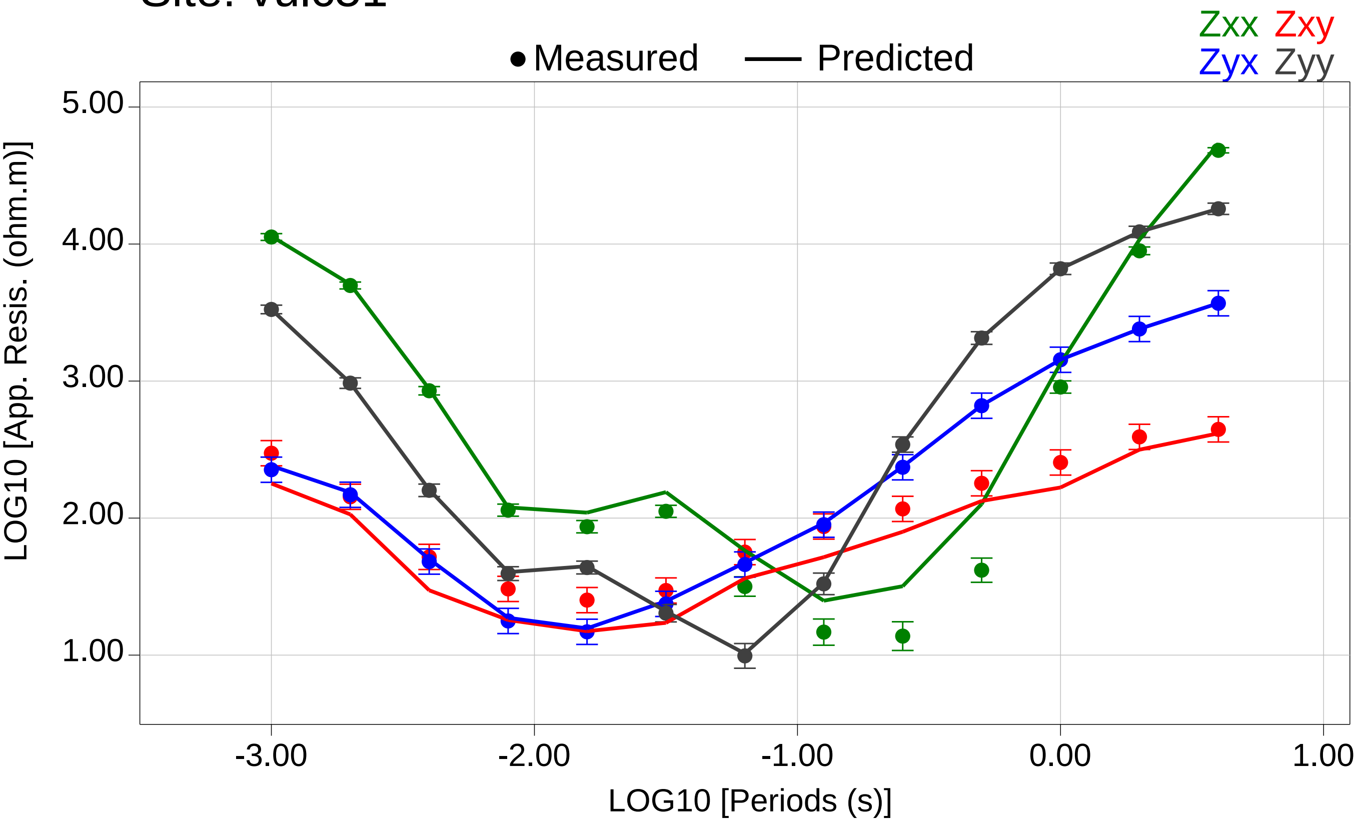

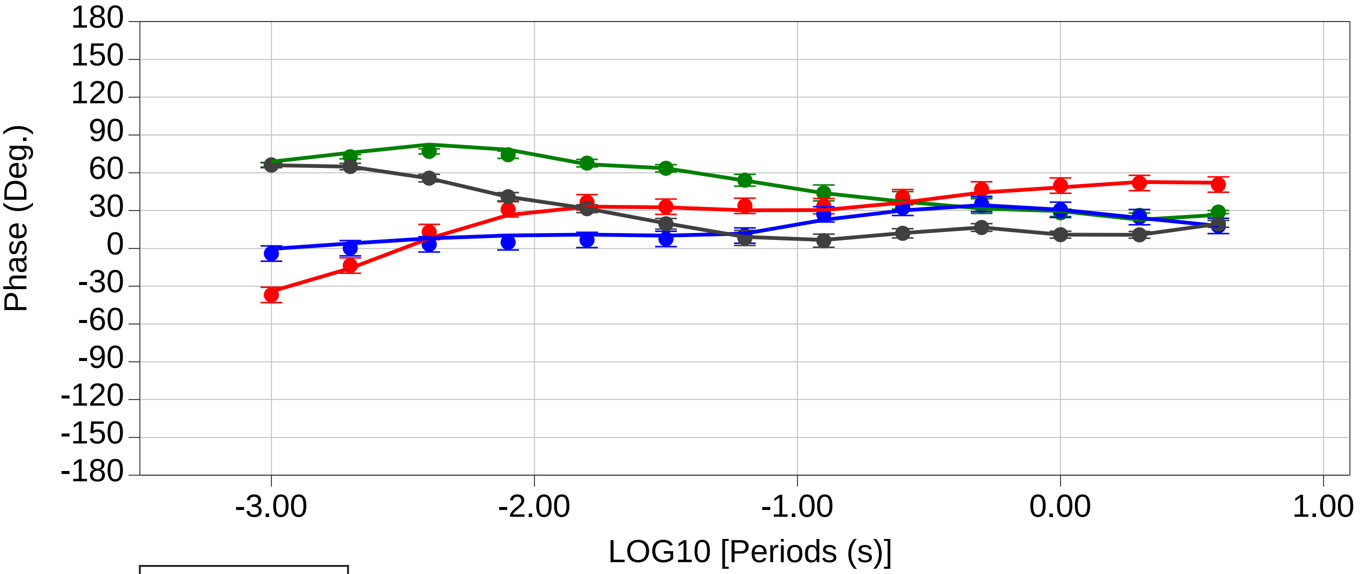

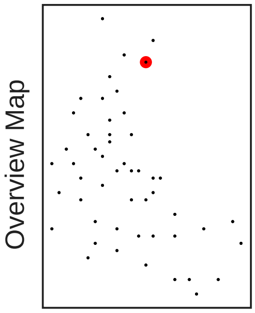

Overall RMS (Z+Tz)= 1.04

Total Z RMS = 1.04

Zxx RMS = 1.83

Zxy RMS = 0.93

Zyx RMS = 0.28

Zyy RMS = 0.07
